# Supplementary material for: Reversible phosphorylation of cyclin T1 promotes assembly and stability of P-TEFb
Source: eLife. 2021 Nov 25;10:e68473. doi: 10.7554/eLife.68473 (PMC8648303; doi:10.7554/eLife.68473)

Reversible phosphorylation of cyclin T1 promotes assembly and stability of P-TEFb

Fig. 1. Critical residues in CycT1 (Thr143 and Thr149) are required for its binding to CDK9

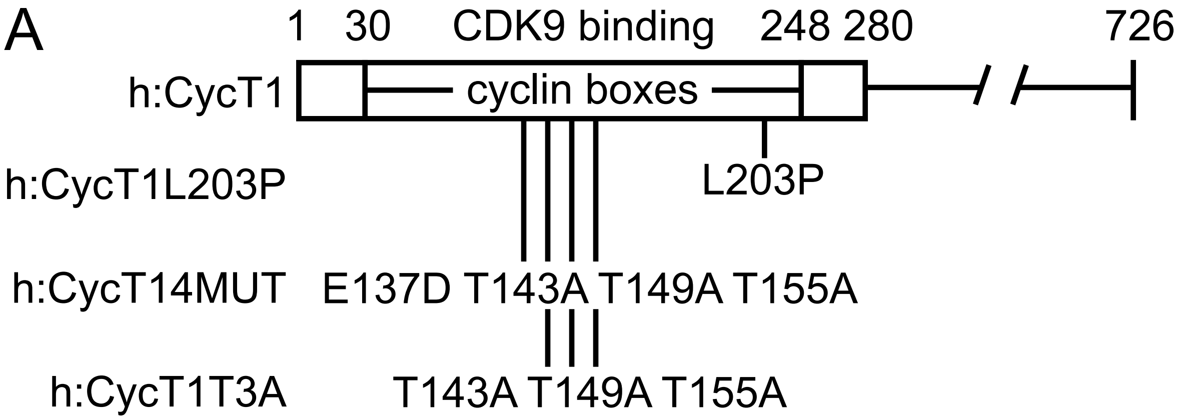

Source blot for Figure 1B

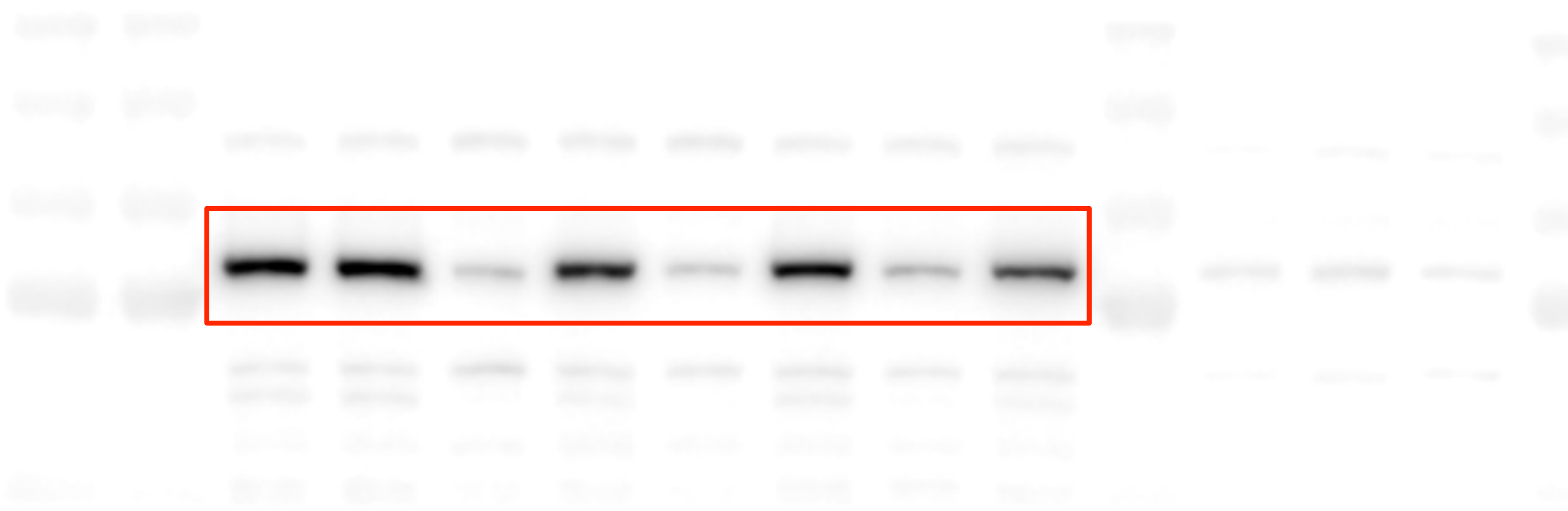

Red marked area was used for the figure.

Membrane was cut after transfer, and before probing by Ab

Source blot for Figure 1B

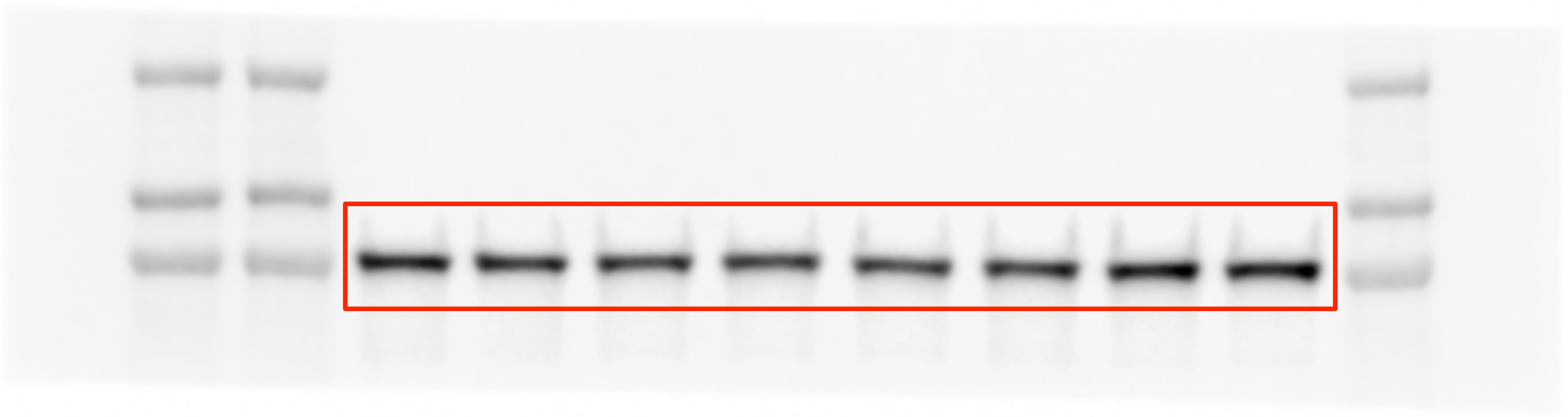

Red marked area was used for the figure.

Membrane was cut after transfer, and before probing by Ab

Source blot for Figure 1B

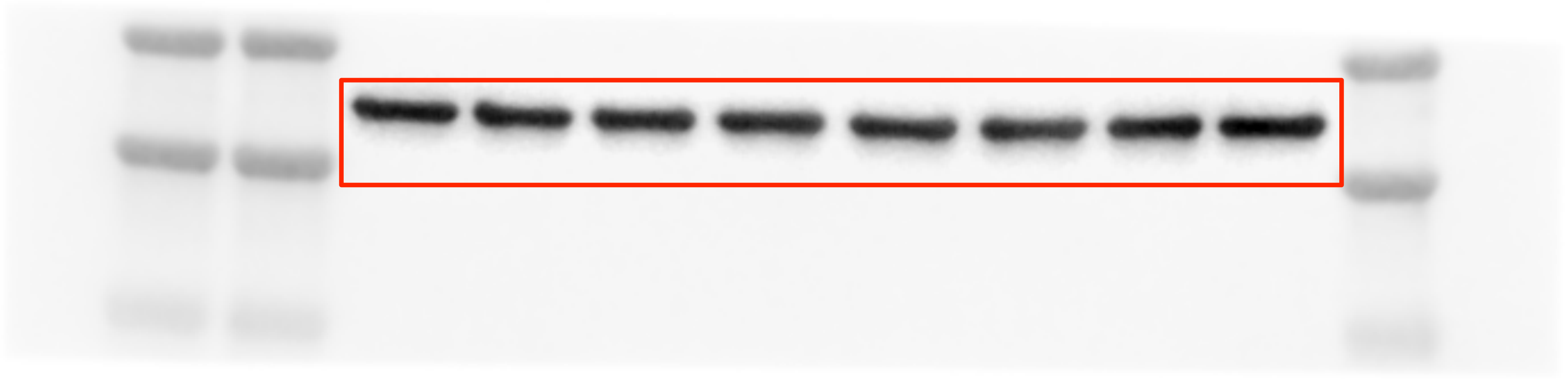

Red marked area was used for the figure.
Membrane was cut after transfer, and before probing by Ab

Fig. 1. Critical residues in CycT1 (Thr143 and Thr149) are required for its binding to CDK9

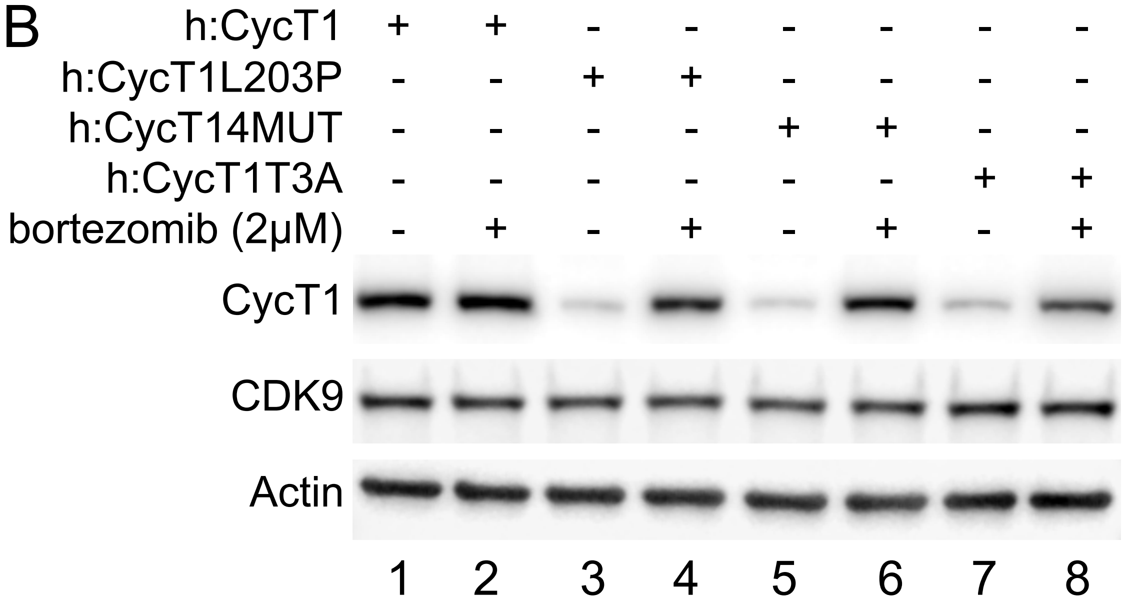

Source blot for Figure 1C

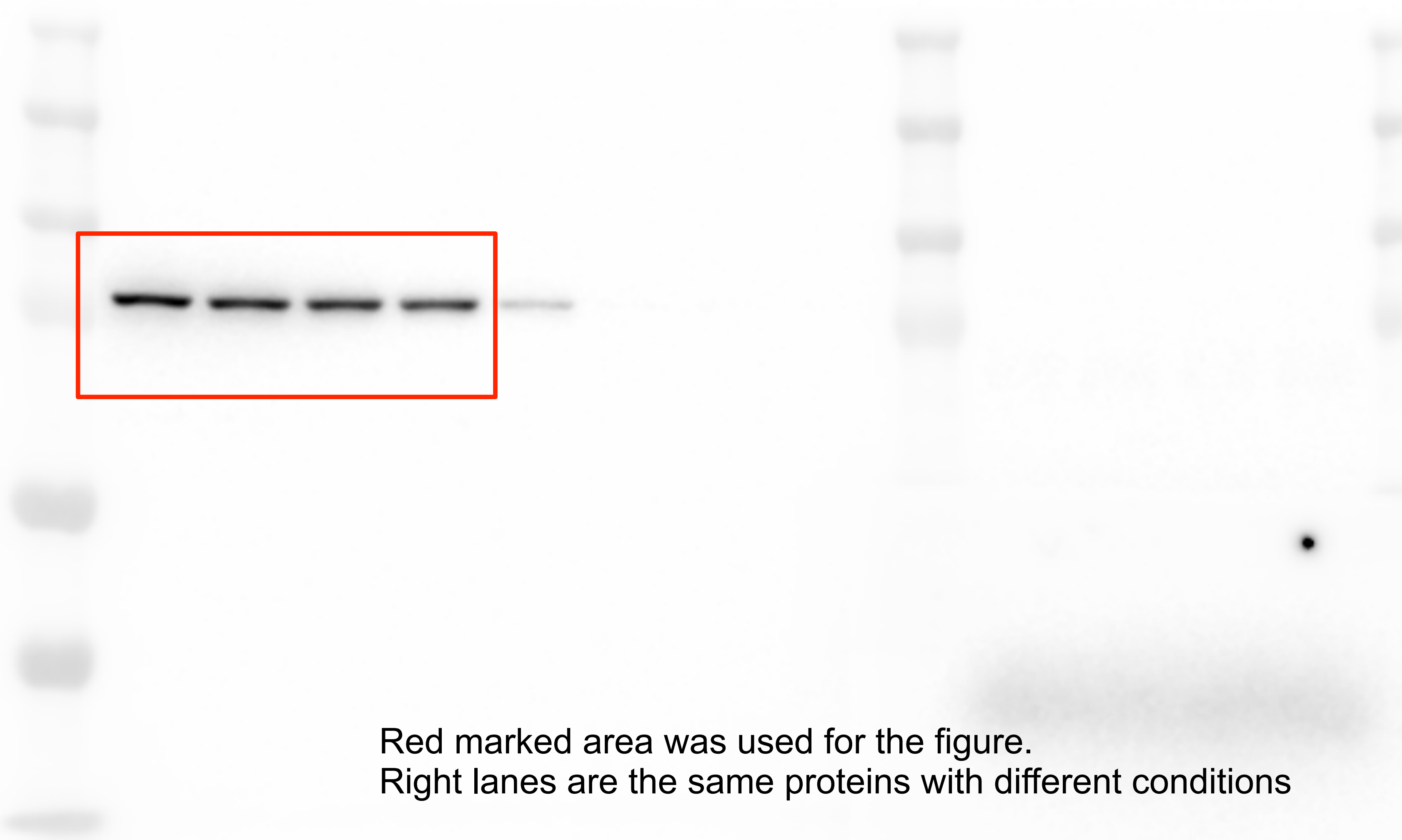

Red marked area was used for the figure.
Right lanes are the same proteins with different conditions

Source blot for Figure 1C

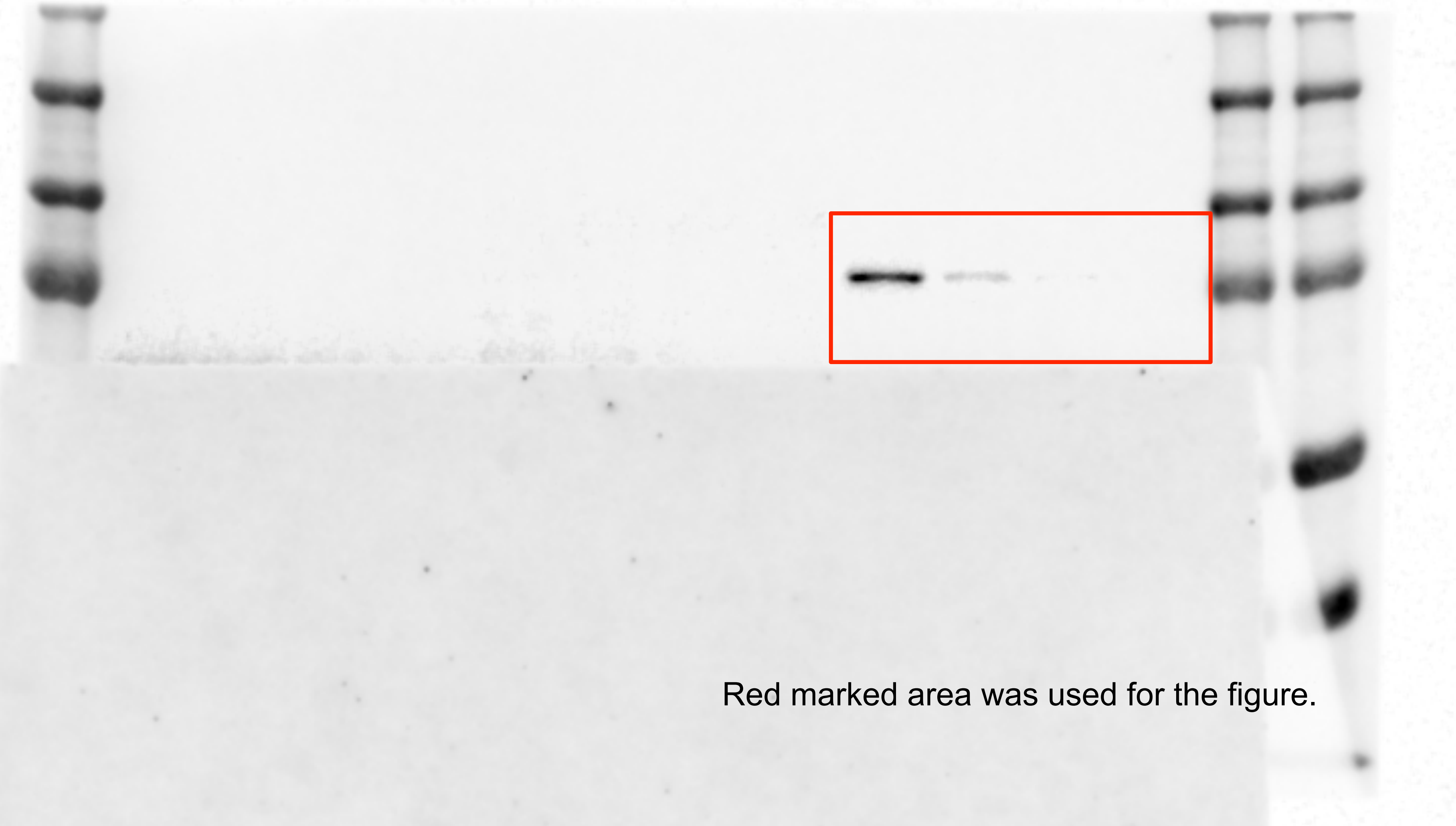

Red marked area was used for the figure.

Source blot for Figure 1C

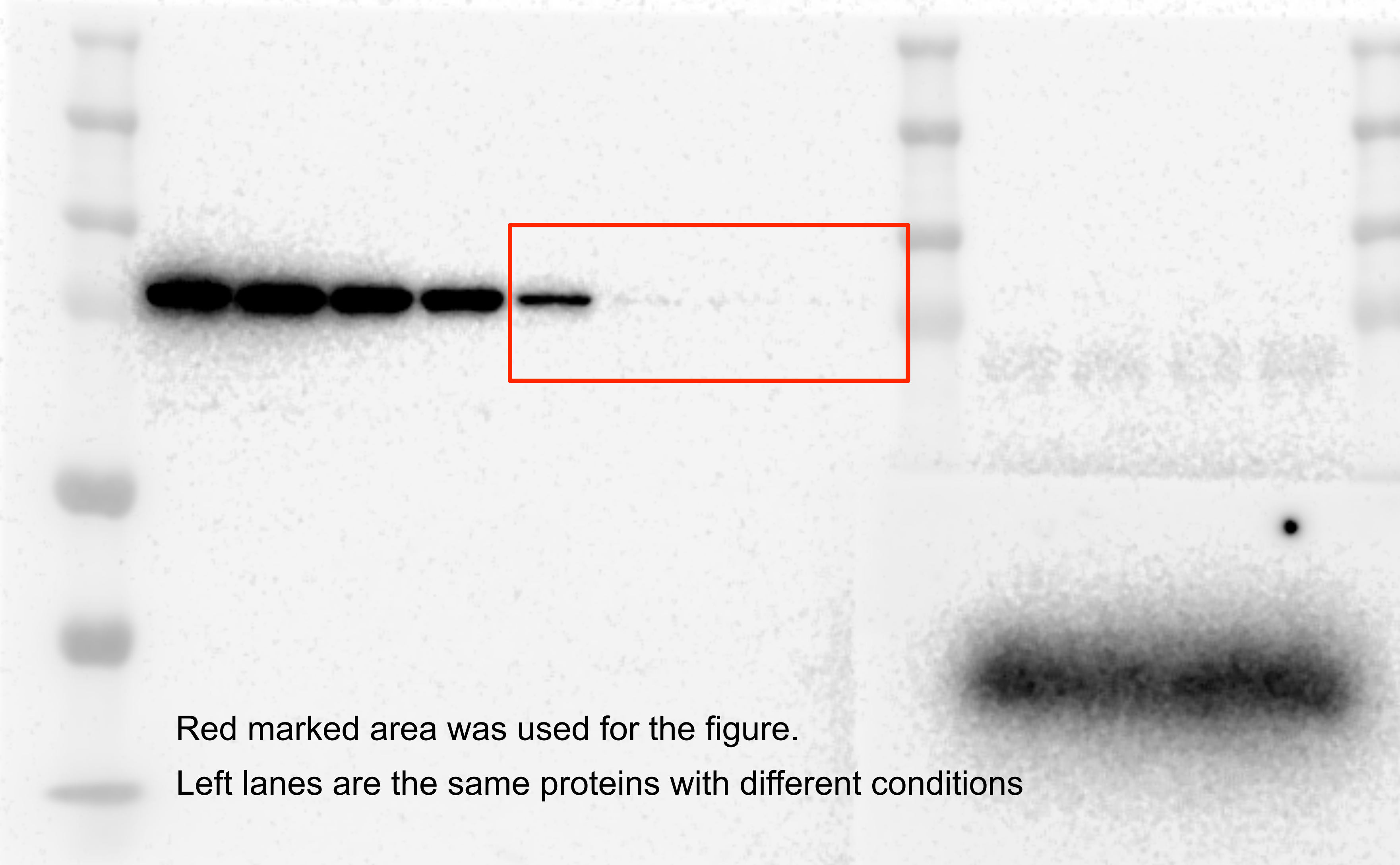

Red marked area was used for the figure.

Left lanes are the same proteins with different conditions

Source blot for Figure 1C

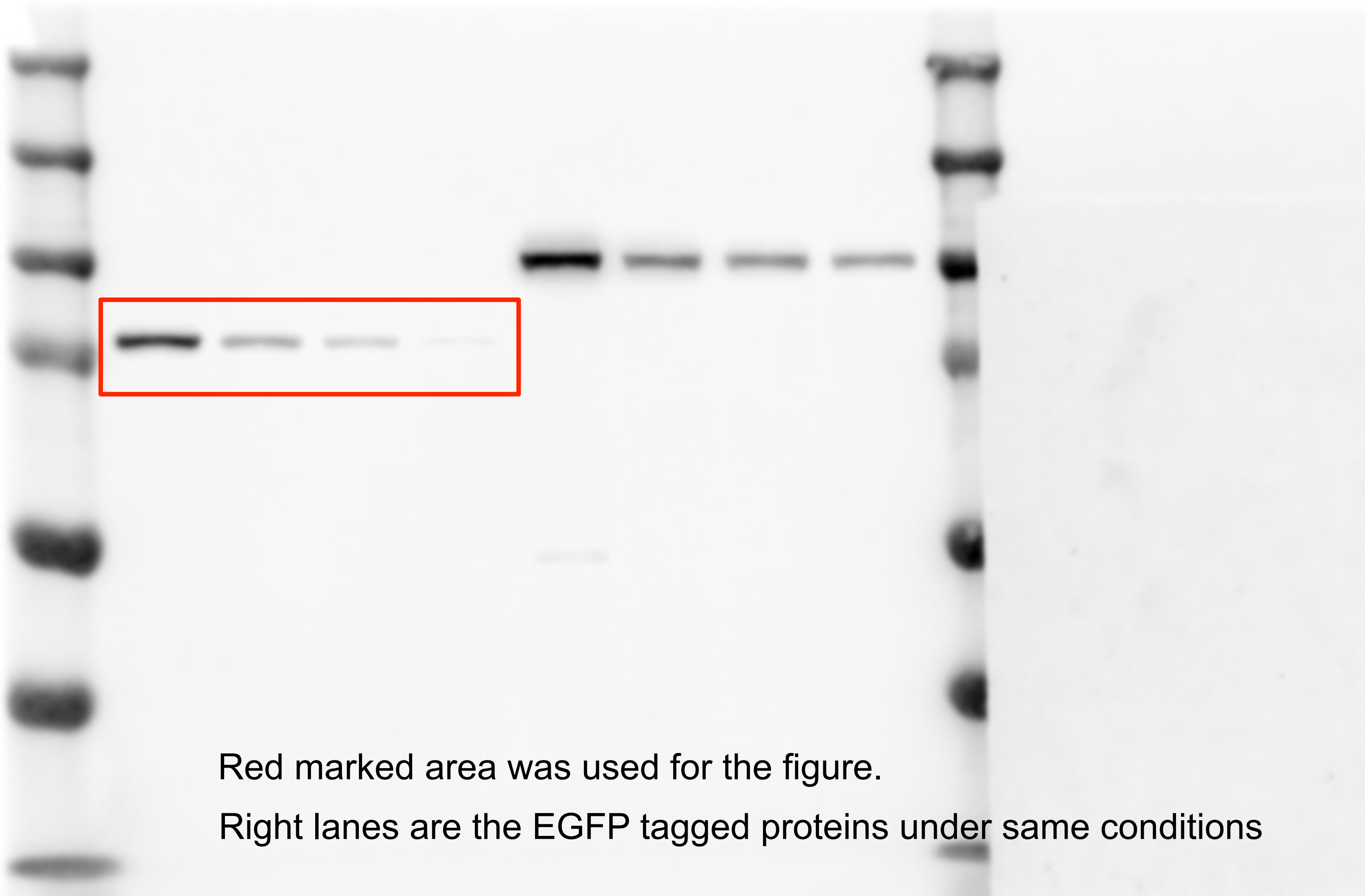

Red marked area was used for the figure.

Right lanes are the EGFP tagged proteins under same conditions

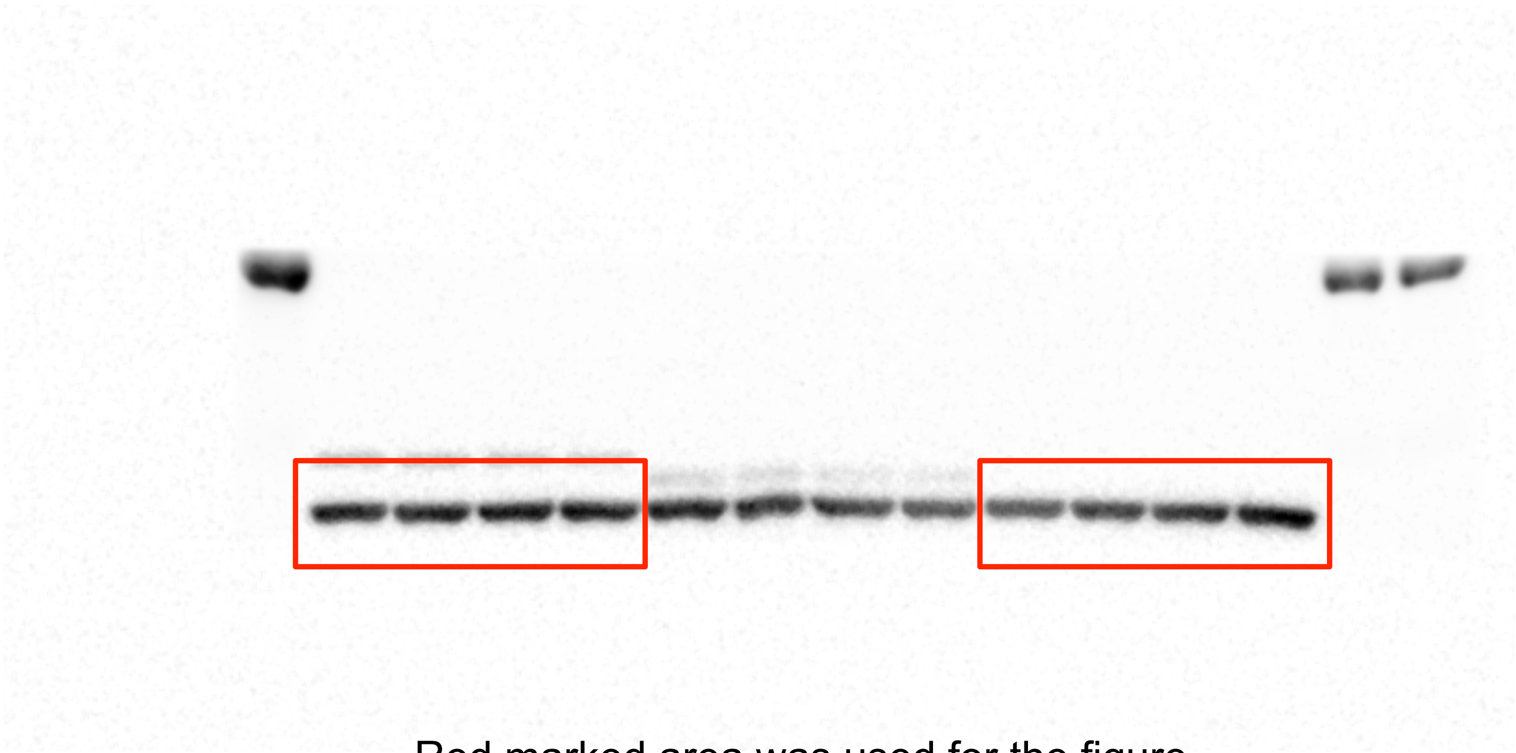

Red marked area was used for the figure.

Middle lanes are the same proteins with different conditions

Membrane was cut after transfer, and before probing by Ab

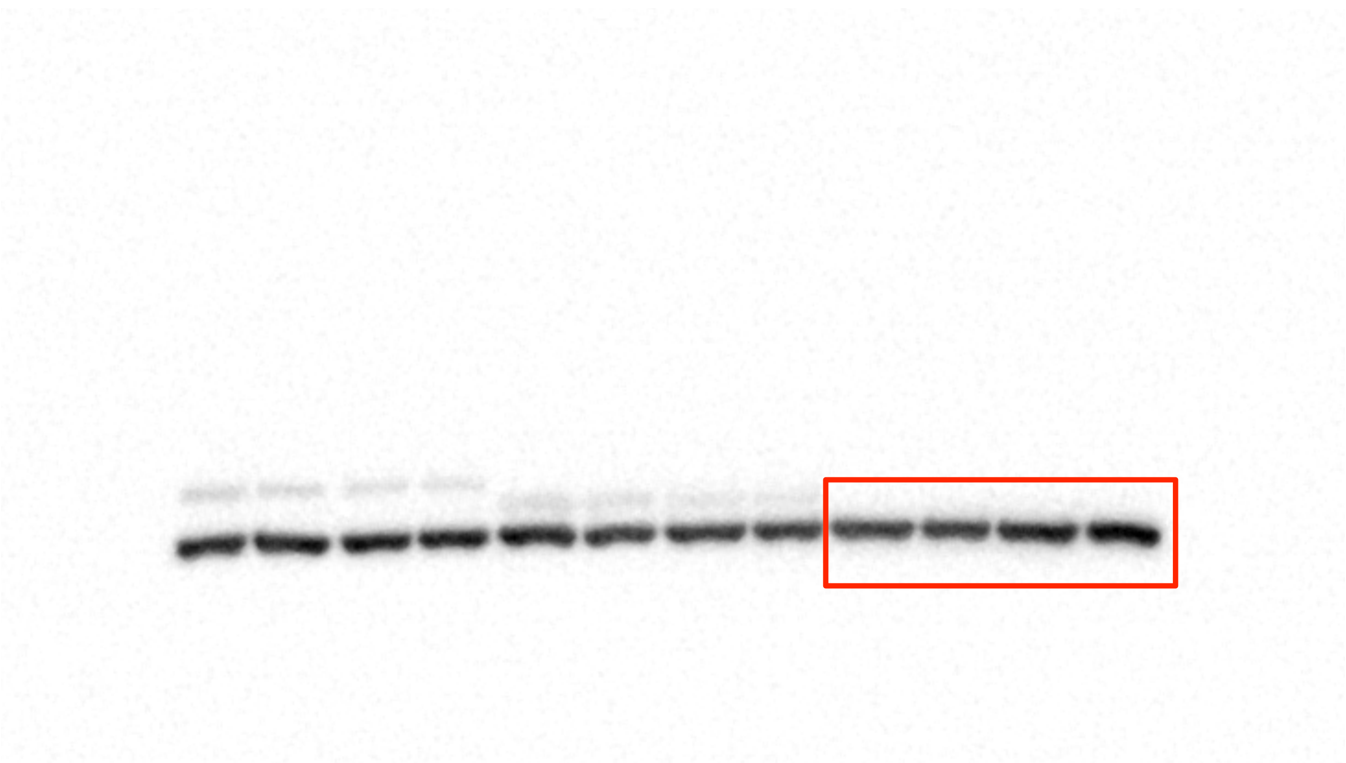

Red marked area was used for the figure.

Left lanes are the same proteins with different conditions

Membrane was cut after transfer, and before probing by Ab

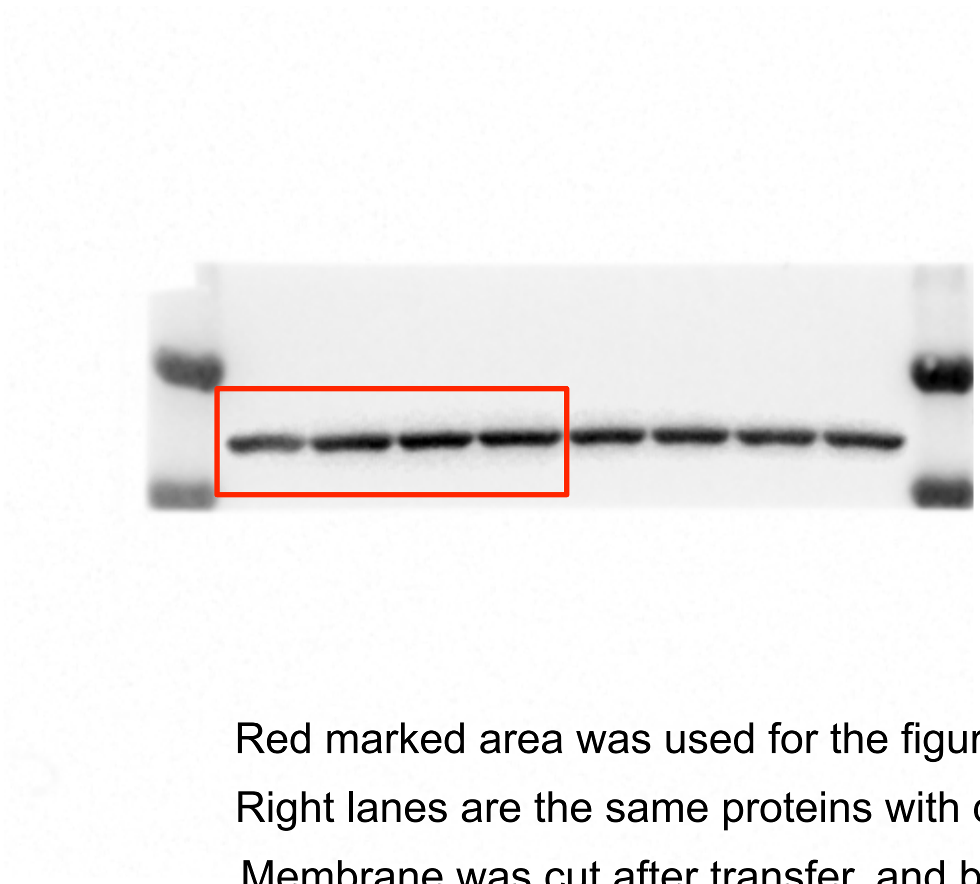

Red marked area was used for the figure.

Right lanes are the same proteins with different conditions

Membrane was cut after transfer, and before probing by Ab

Fig. 1. Critical residues in CycT1 (Thr143 and Thr149) are required for its binding to CDK9

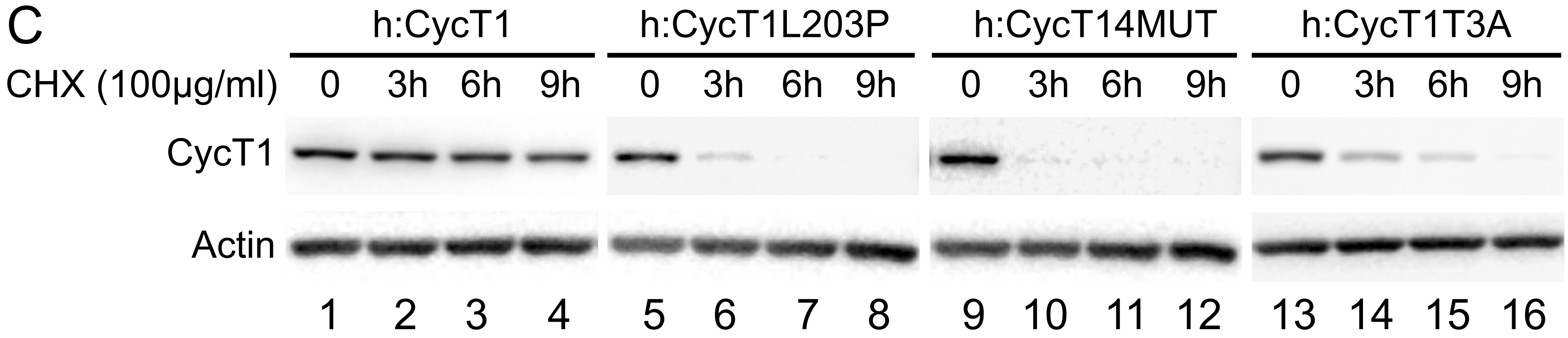

Source blot for Figure 1D

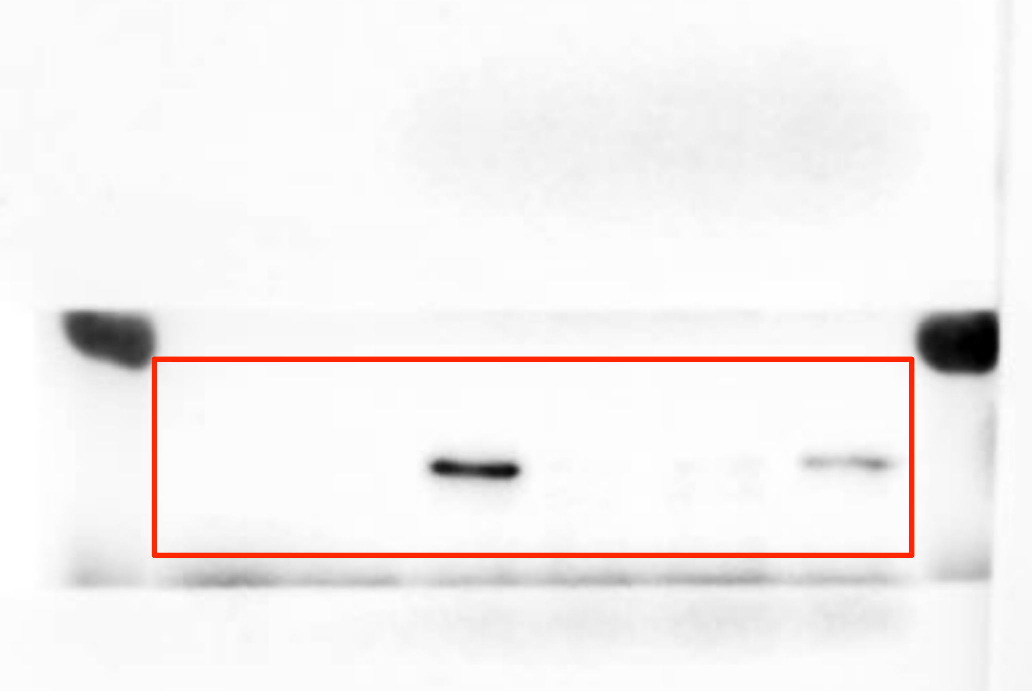

Red marked area was used for the figure.

Source blot for Figure 1D

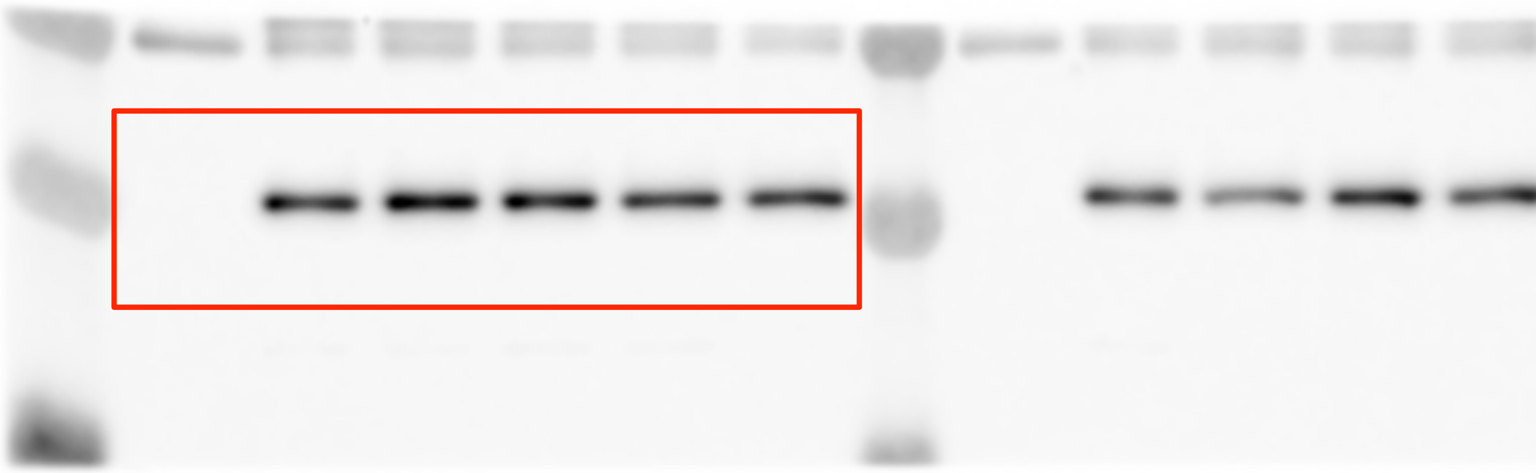

Red marked area was used for the figure.

Membrane was cut after transfer, and before probing by Ab
Right lanes are the same proteins under different conditions

Source blot for Figure 1D

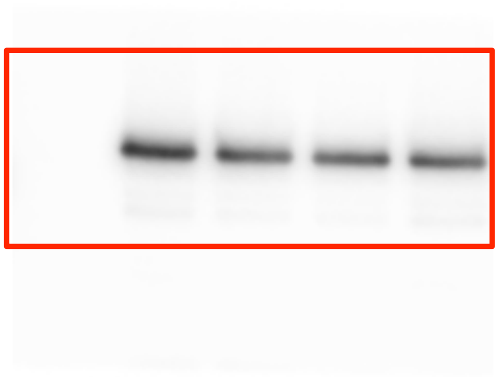

Red marked area was used for the figure.

Membrane was cut after transfer, and before probing by Ab

Source blot for Figure 1D

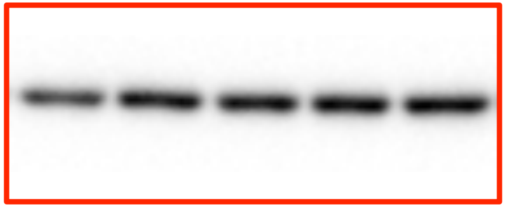

Red marked area was used for the figure.

Membrane was cut after transfer, and before probing by Ab

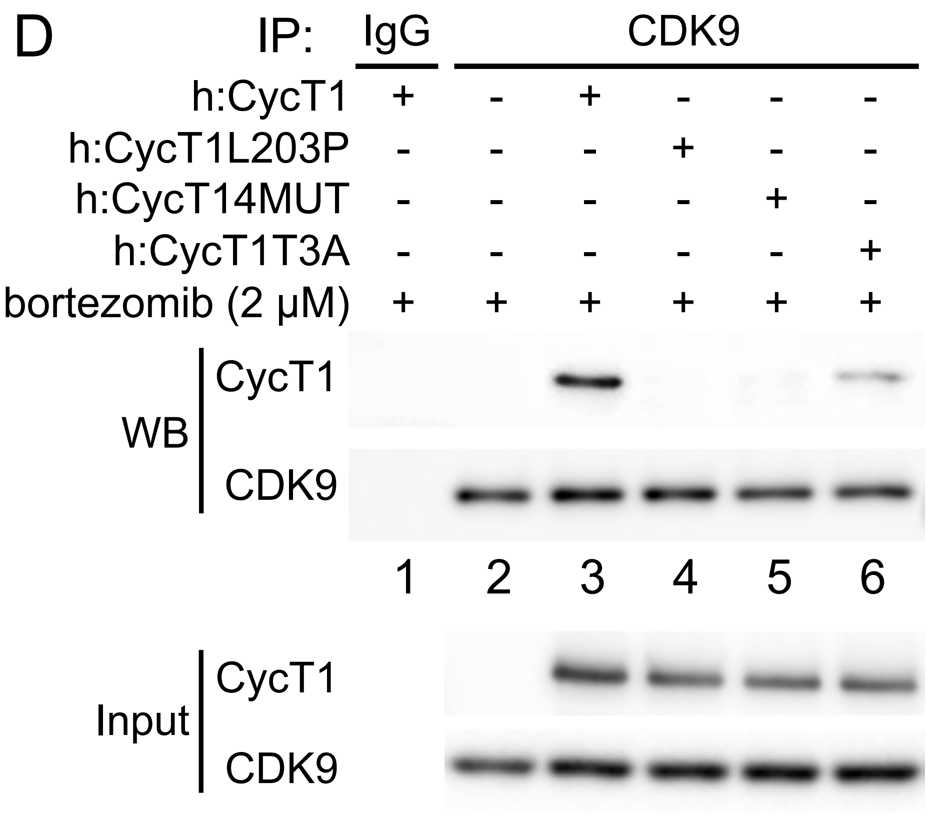
D
IP: IgG CDK9
h:CycT1 + - + - - -
h:CycT1L203P - - - + - -
h:CycT14MUT - - - - + -
h:CycT1T3A - - - - - +
bortezomib (2 μM) + + + + + +
WB
CycT1
CDK9
1 2 3 4 5 6
Input
CycT1
CDK9

Source blot for Figure 1E

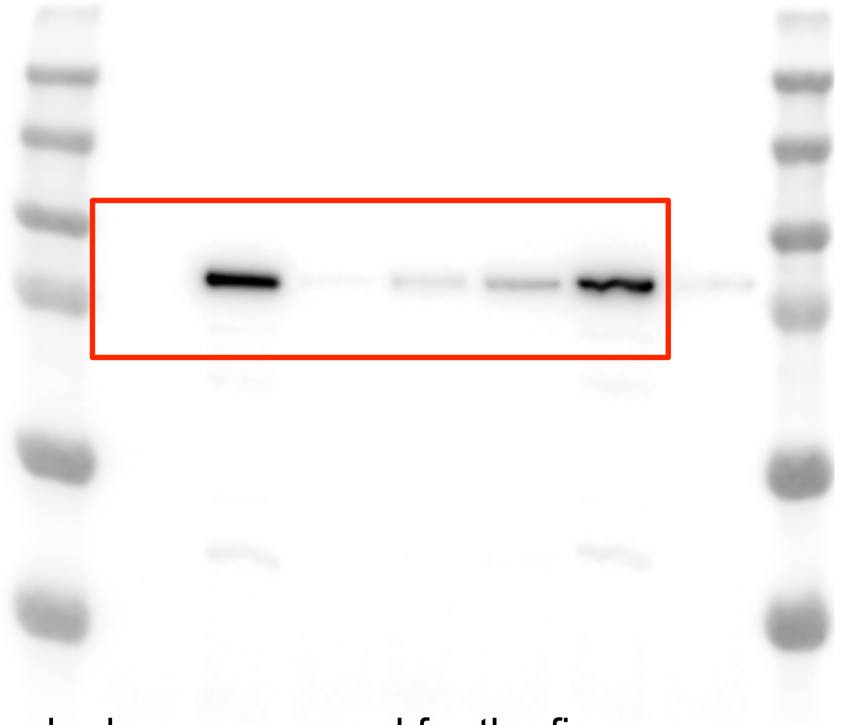

Red marked area was used for the figure.

Right lane are the same protein for the control, CycT1TT143149AA

Source blot for Figure 1E

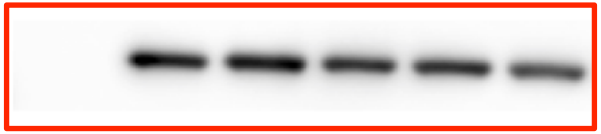

Red marked area was used for the figure.

Membrane was cut after transfer, and before probing by Ab

Source blot for Figure 1E

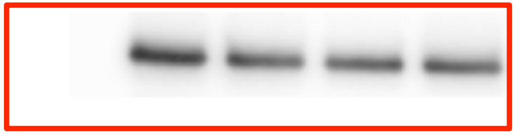

Red marked area was used for the figure.

Membrane was cut after transfer, and before probing by Ab

Source blot for Figure 1E

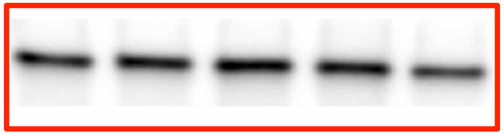

Red marked area was used for the figure.
Membrane was cut after transfer, and before probing by Ab

Fig. 1. Critical residues in CycT1 (Thr143 and Thr149) are required for its binding to CDK9

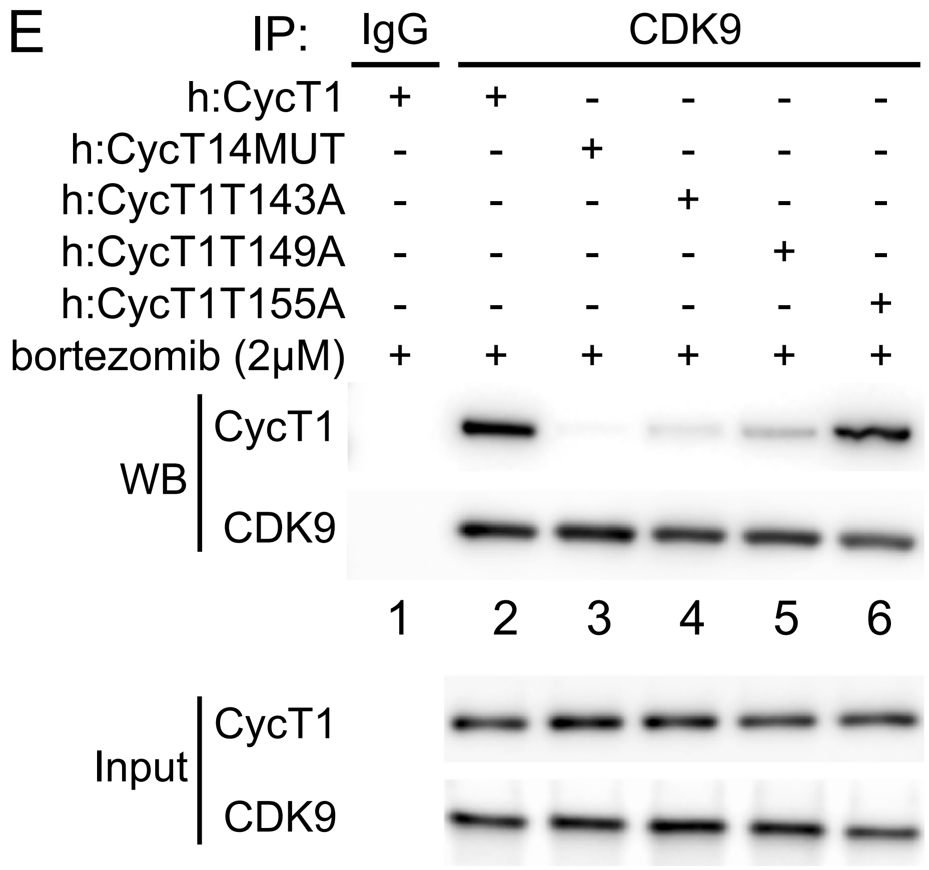

Source blot for Figure 1F

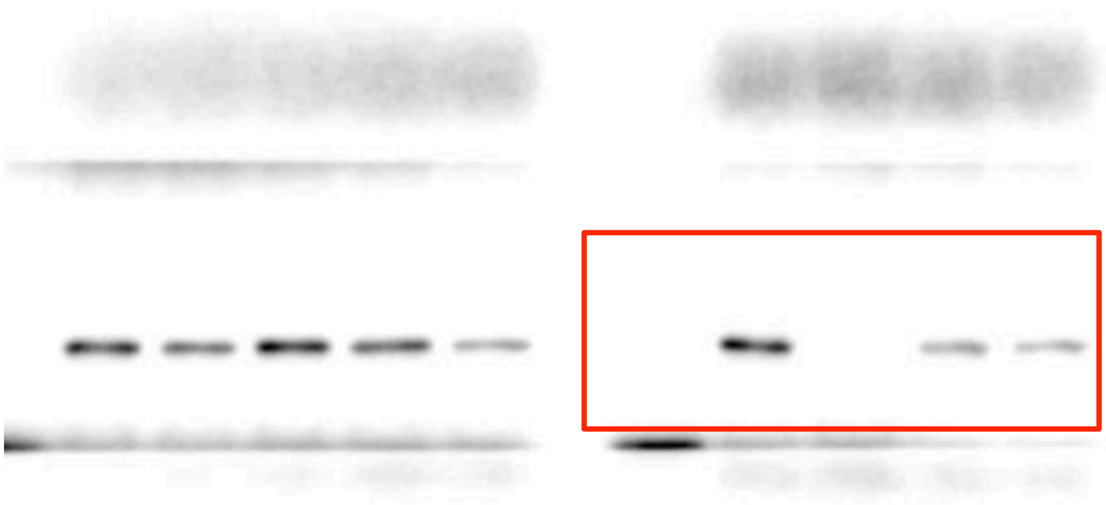

Red marked area was used for the figure.

Left lanes are same proteins for other conditions

Source blot for Figure 1F

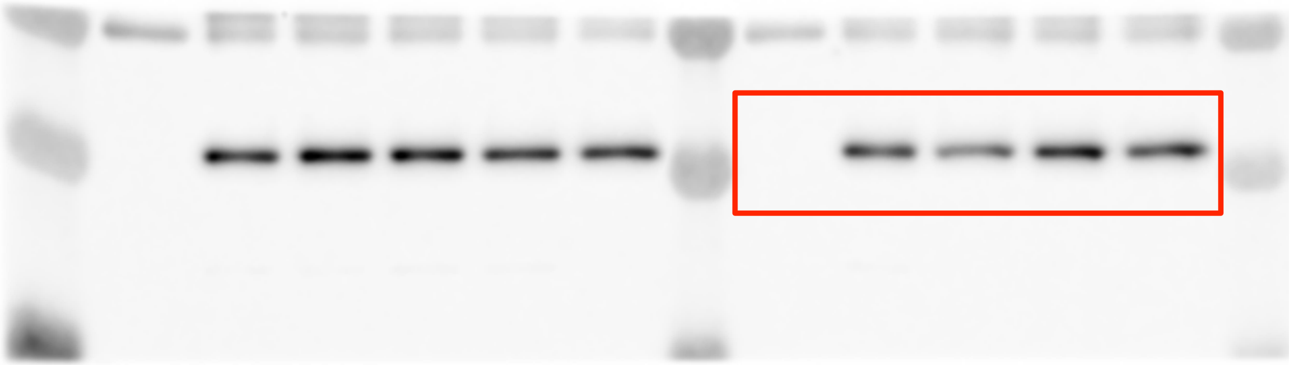

Red marked area was used for the figure.

Membrane was cut after transfer, and before probing by Ab

Left lanes are same proteins for other conditions

Source blot for Figure 1F

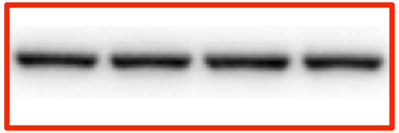

Red marked area was used for the figure.

Membrane was cut after transfer, and before probing by Ab

Source blot for Figure 1F

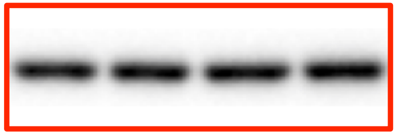

Red marked area was used for the figure.

Membrane was cut after transfer, and before probing by Ab

Fig. 1. Critical residues in CycT1 (Thr143 and Thr149) are required for its binding to CDK9

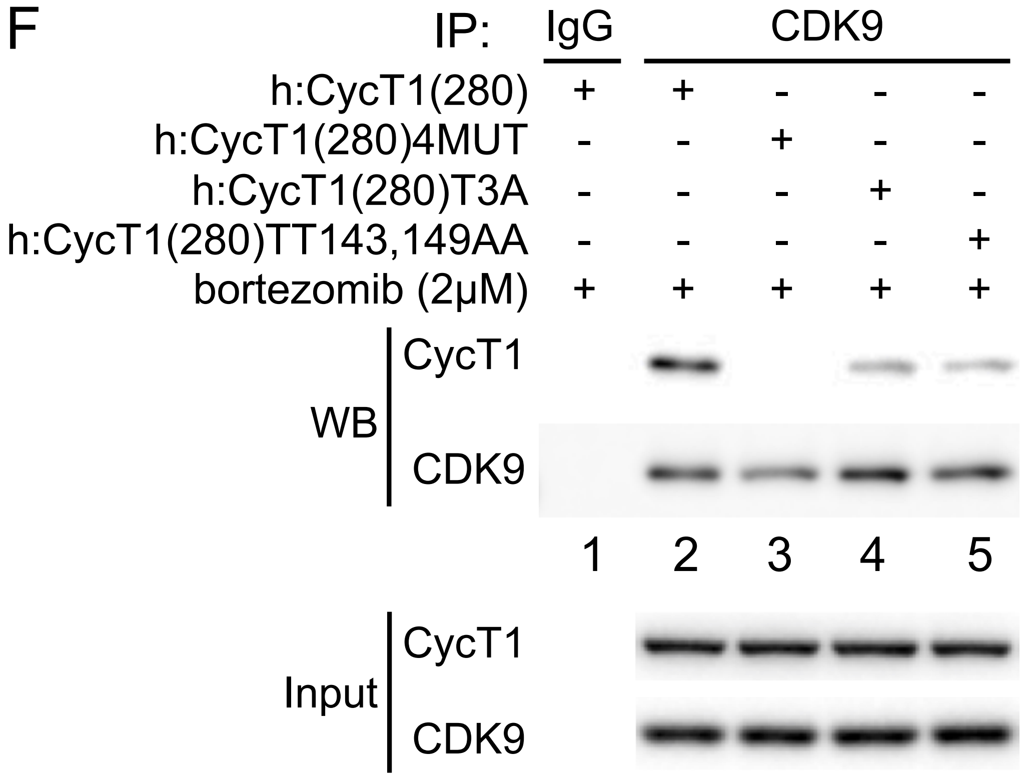

Source blot for Figure 2A

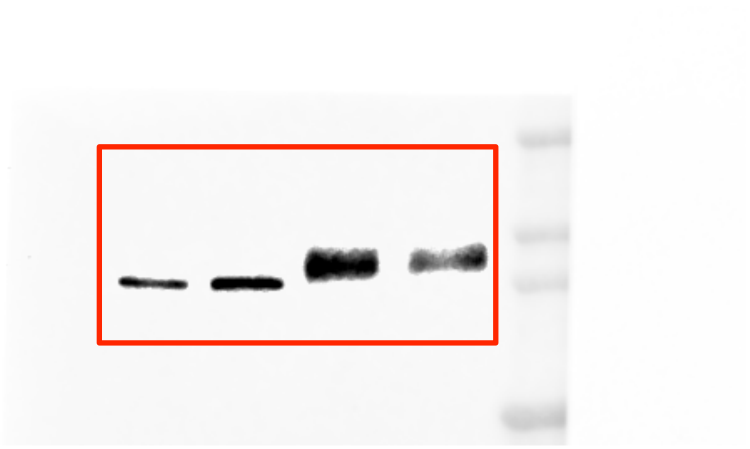

Red marked area was used for the figure.

Membrane was cut after transfer, and before probing by Ab

Source blot for Figure 2A

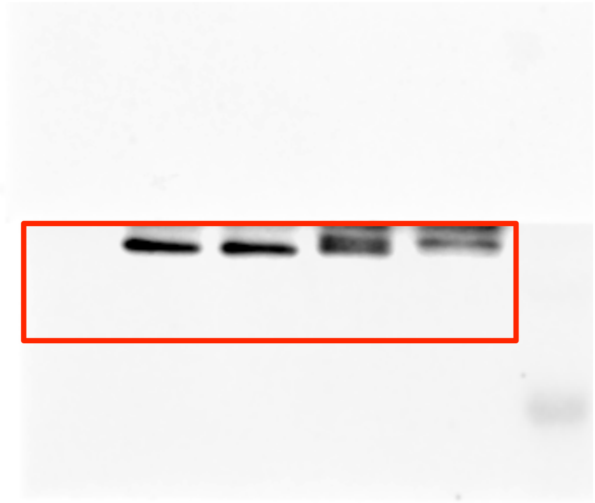

Red marked area was used for the figure.

Membrane was cut after transfer, and before probing by Ab

Source blot for Figure 2A

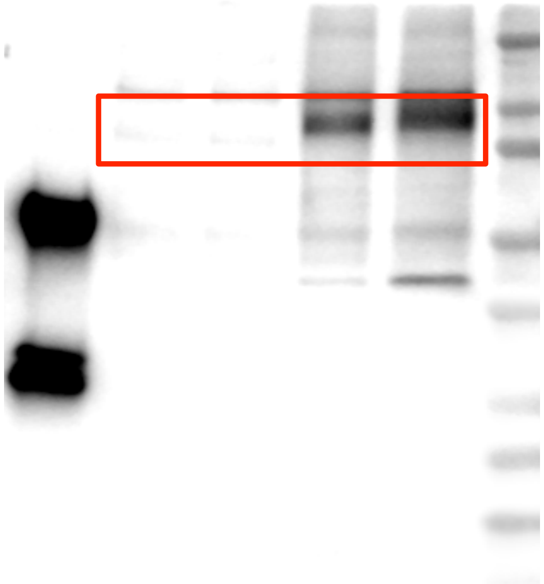

Red marked area was used for the figure.
Membrane was cut after transfer, and before probing by Ab

Source blot for Figure 2A

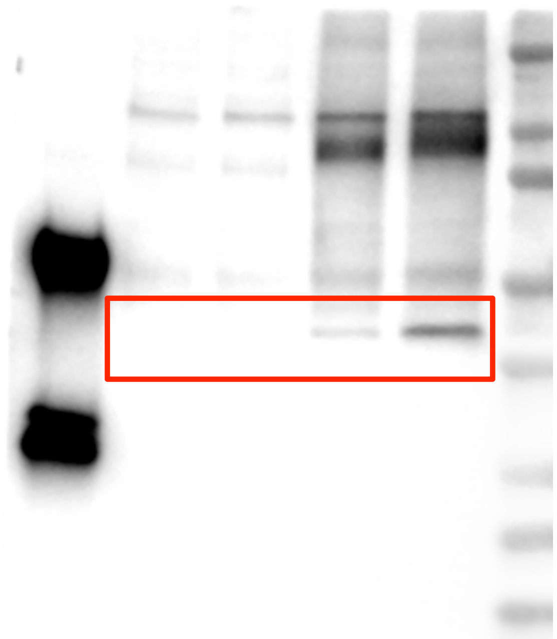

Red marked area was used for the figure.

Membrane was cut after transfer, and before probing by Ab

Source blot for Figure 2A

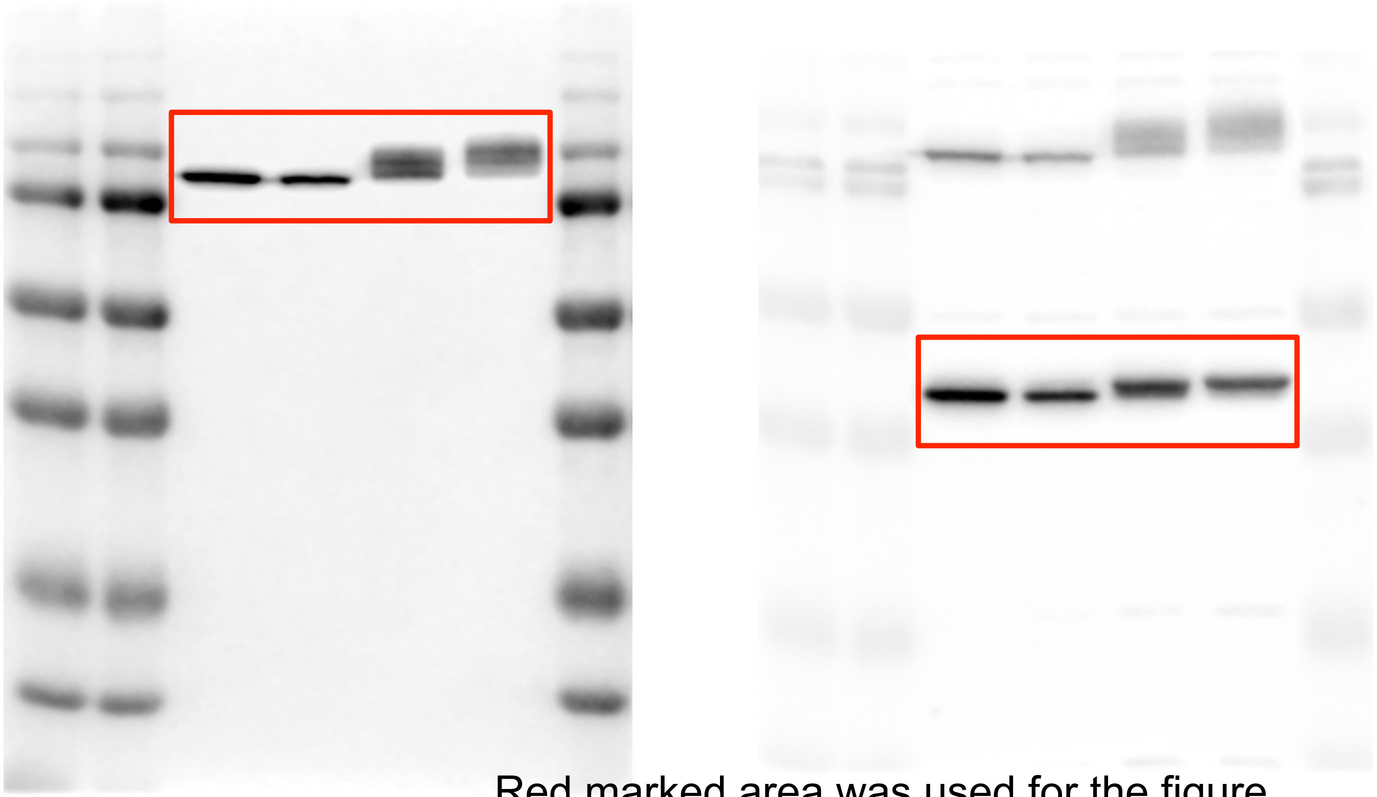

Red marked area was used for the figure.

Membrane was cut after transfer, and before probing by Ab

Two input proteins blots are in the same slide

Fig. 2. Phosphorylation of Thr143 and Thr149 in CycT1 contributes to its binding to CDK9

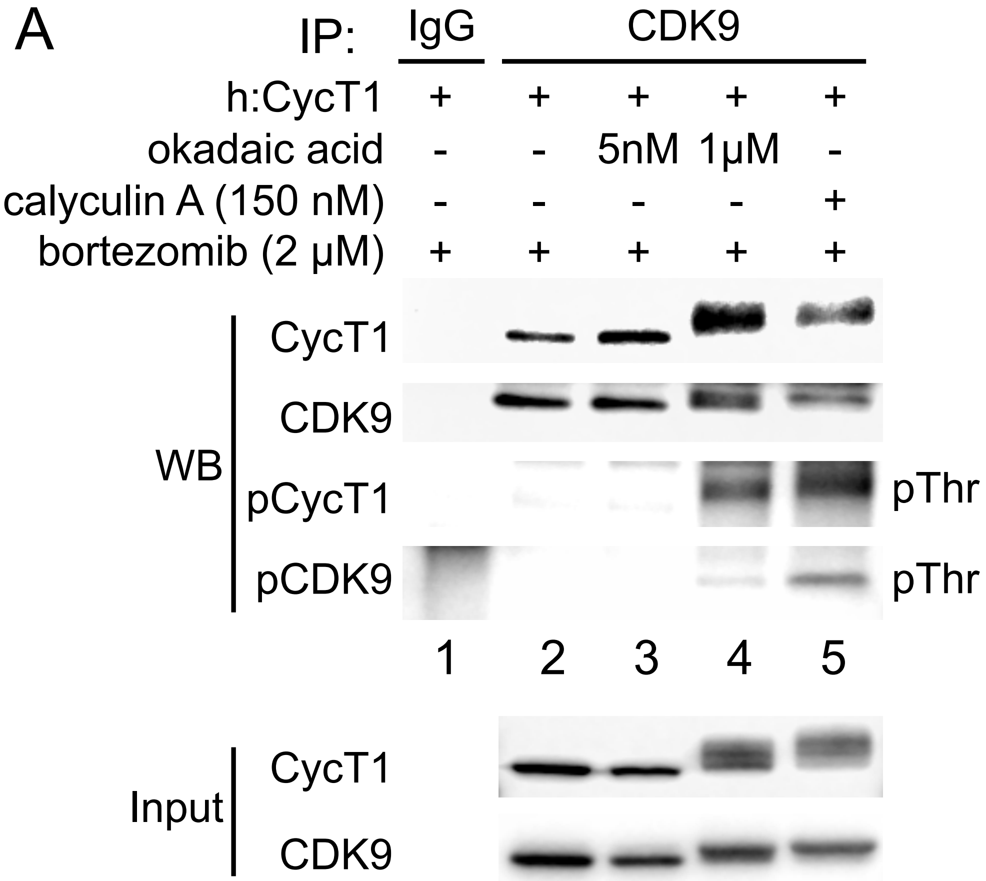

Source blot for Figure 2B

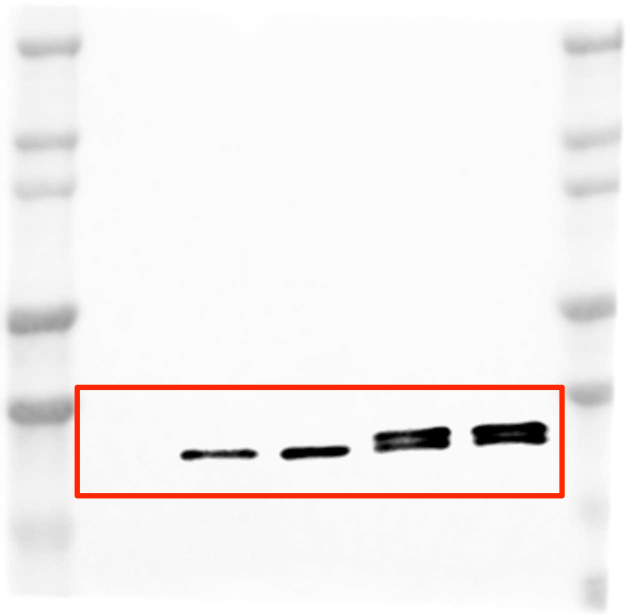

Red marked area was used for the figure.

Membrane was cut after transfer, and before probing by Ab

Source blot for Figure 2B

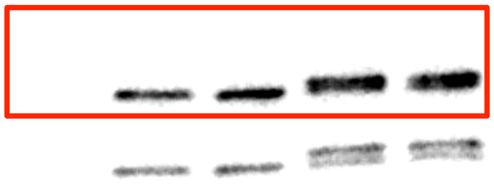

Red marked area was used for the figure.

Membrane was cut after transfer, and before probing by Ab

Source blot for Figure 2B

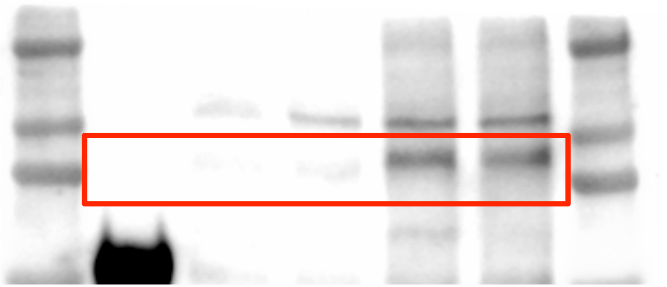

Red marked area was used for the figure.

Membrane was cut after transfer, and before probing by Ab

Source blot for Figure 2B

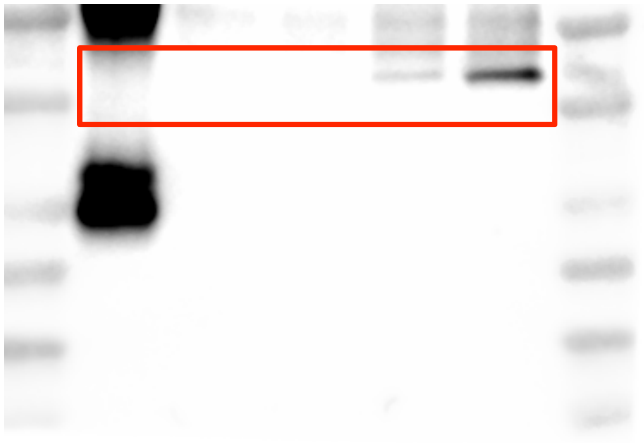

Red marked area was used for the figure.

Membrane was cut after transfer, and before probing by Ab

Source blot for Figure 2B

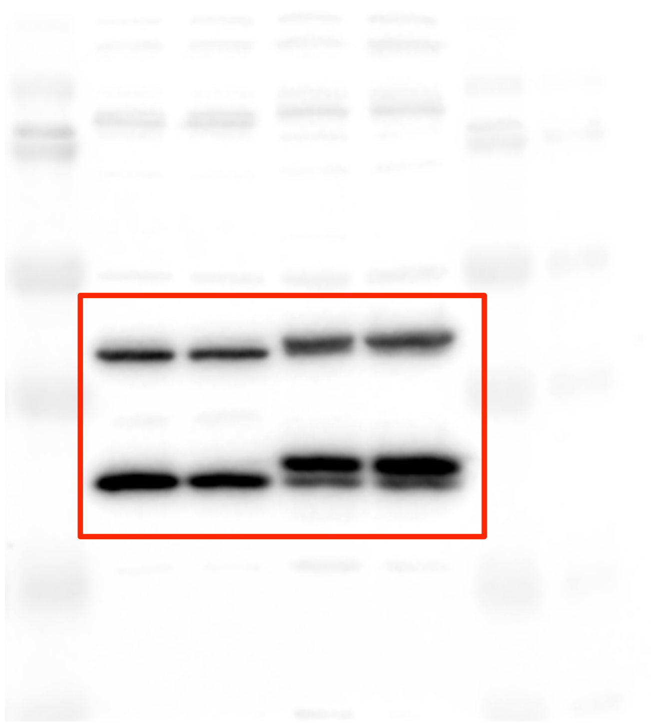

Red marked area was used for the figure.

Membrane was cut after transfer, and before probing by Ab
Two antibodies are used in the same time for input proteins

Fig. 2. Phosphorylation of Thr143 and Thr149 in CycT1 contributes to its binding to CDK9

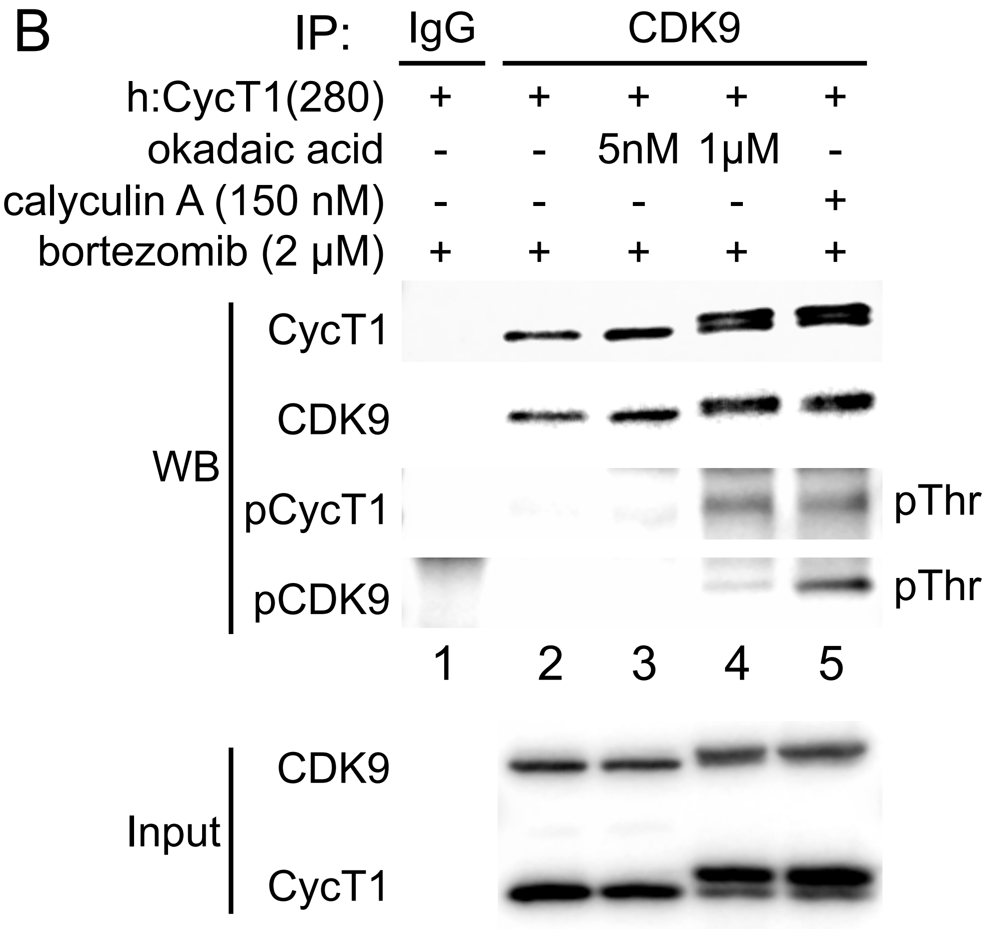

Source blot for Figure 2C

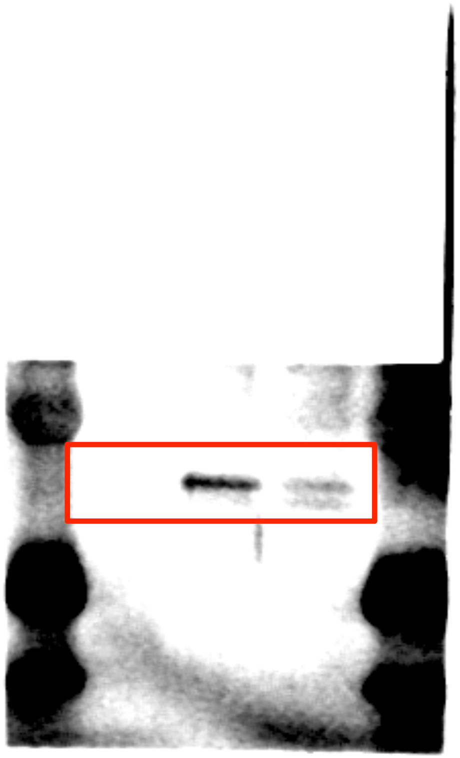

Red marked area was used for the figure.

Source blot for Figure 2C

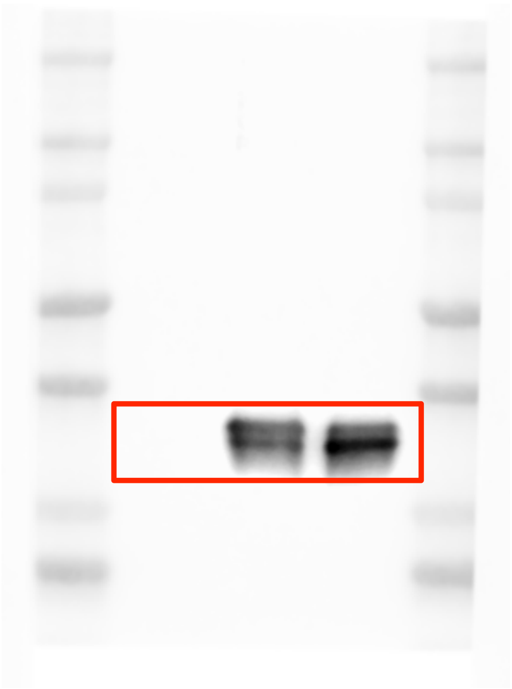

Red marked area was used for the figure.

Source blot for Figure 2C

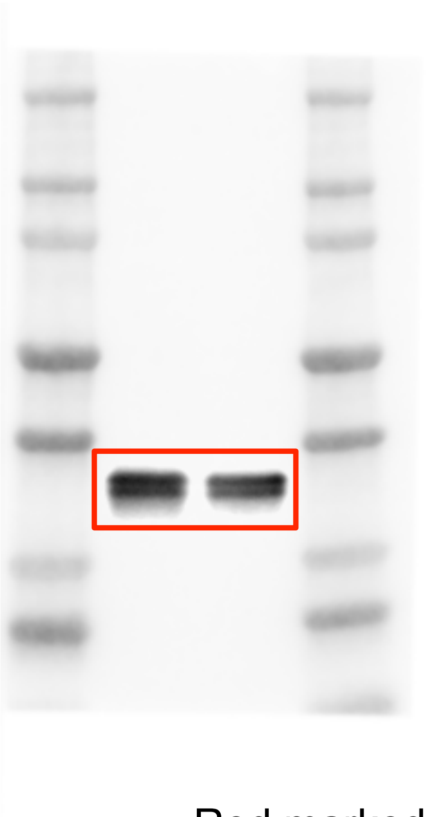

Red marked area was used for the figure.

Fig. 2. Phosphorylation of Thr143 and Thr149 in CycT1 contributes to its binding to CDK9

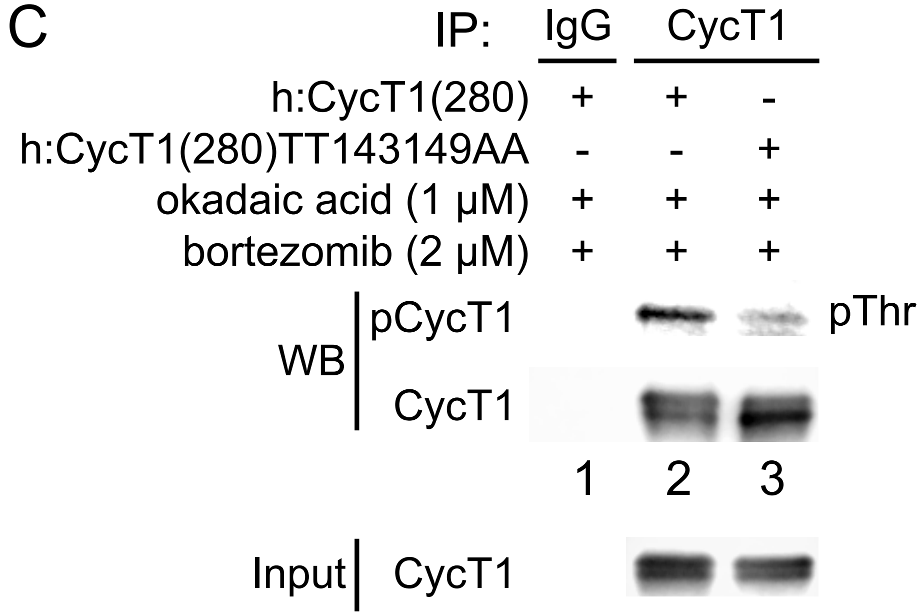

Source gel-staining for Figure 2D

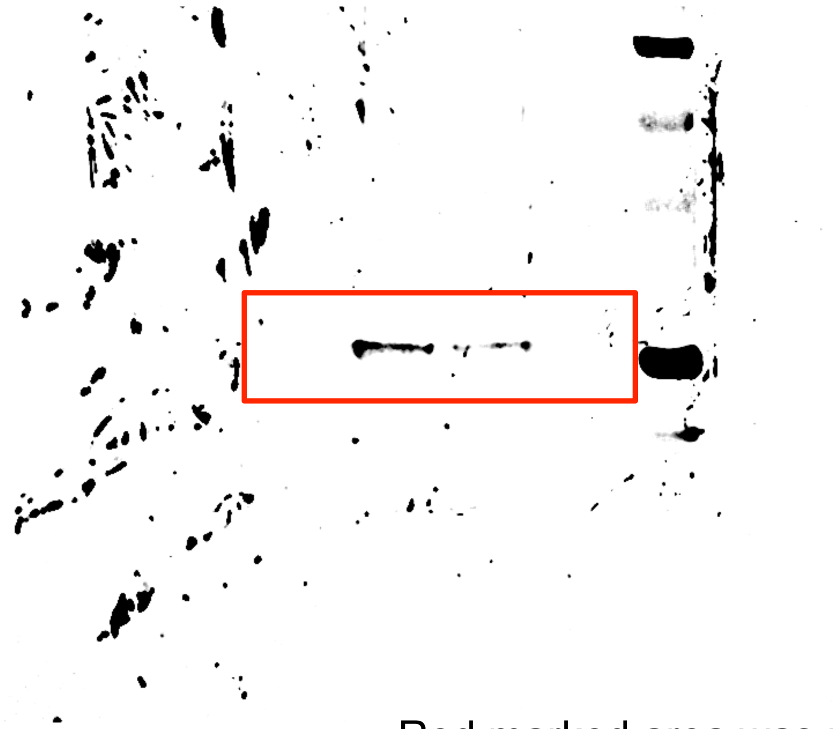

Red marked area was used for the figure.

Fig. 2. Phosphorylation of Thr143 and Thr149 in CycT1 contributes to its binding to CDK9

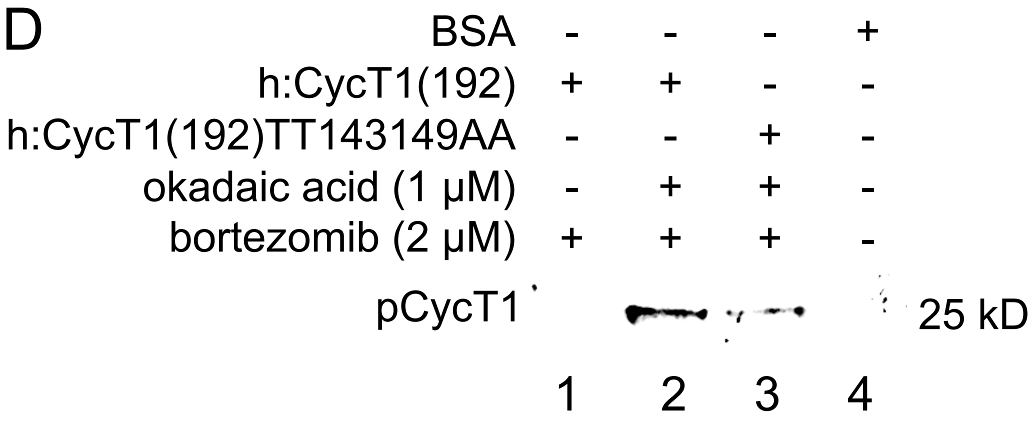

Source gel staining for Figure 2E

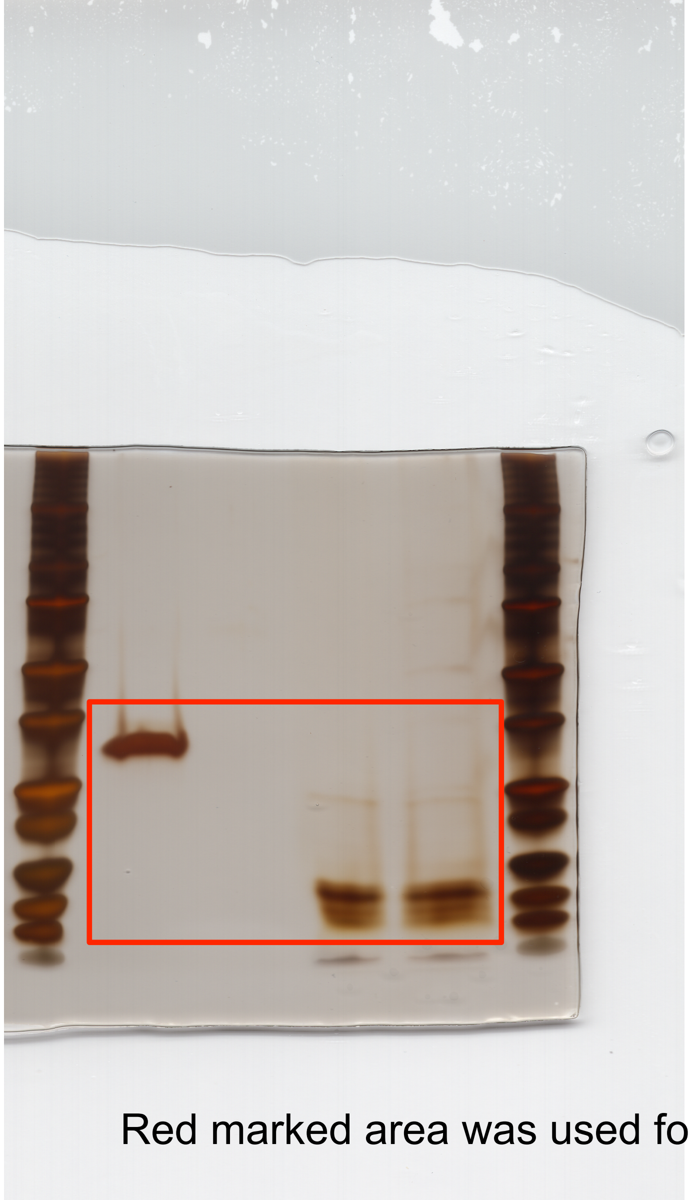

Red marked area was used for the figure.

Fig. 2. Phosphorylation of Thr143 and Thr149 in CycT1 contributes to its binding to CDK9

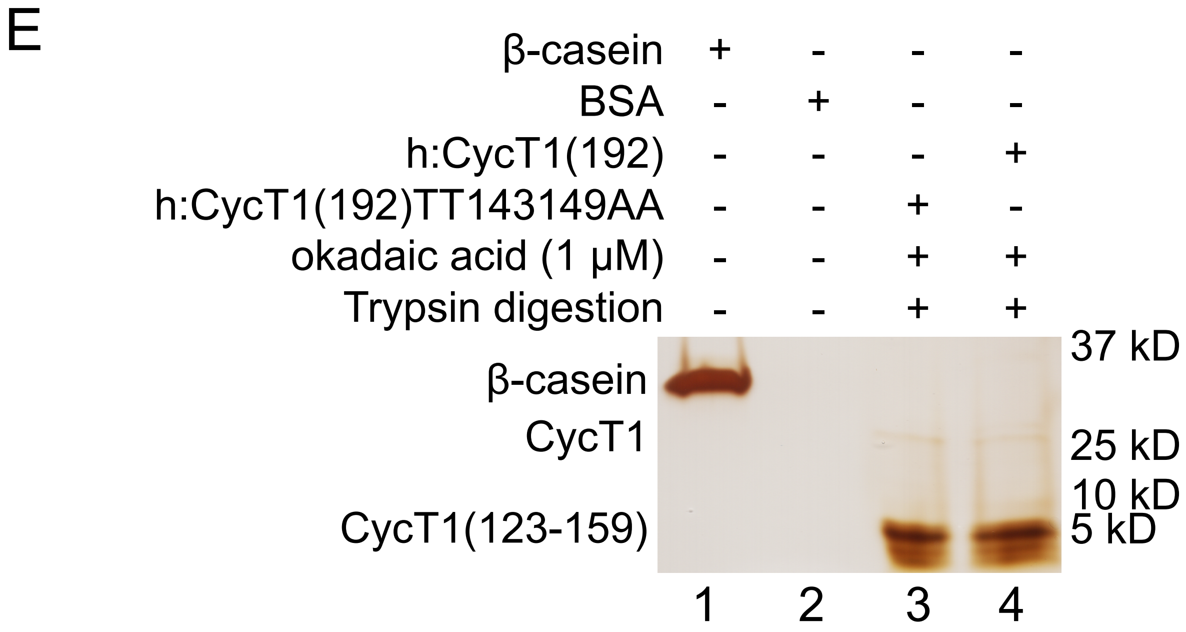

Source gel staining for Figure 2E

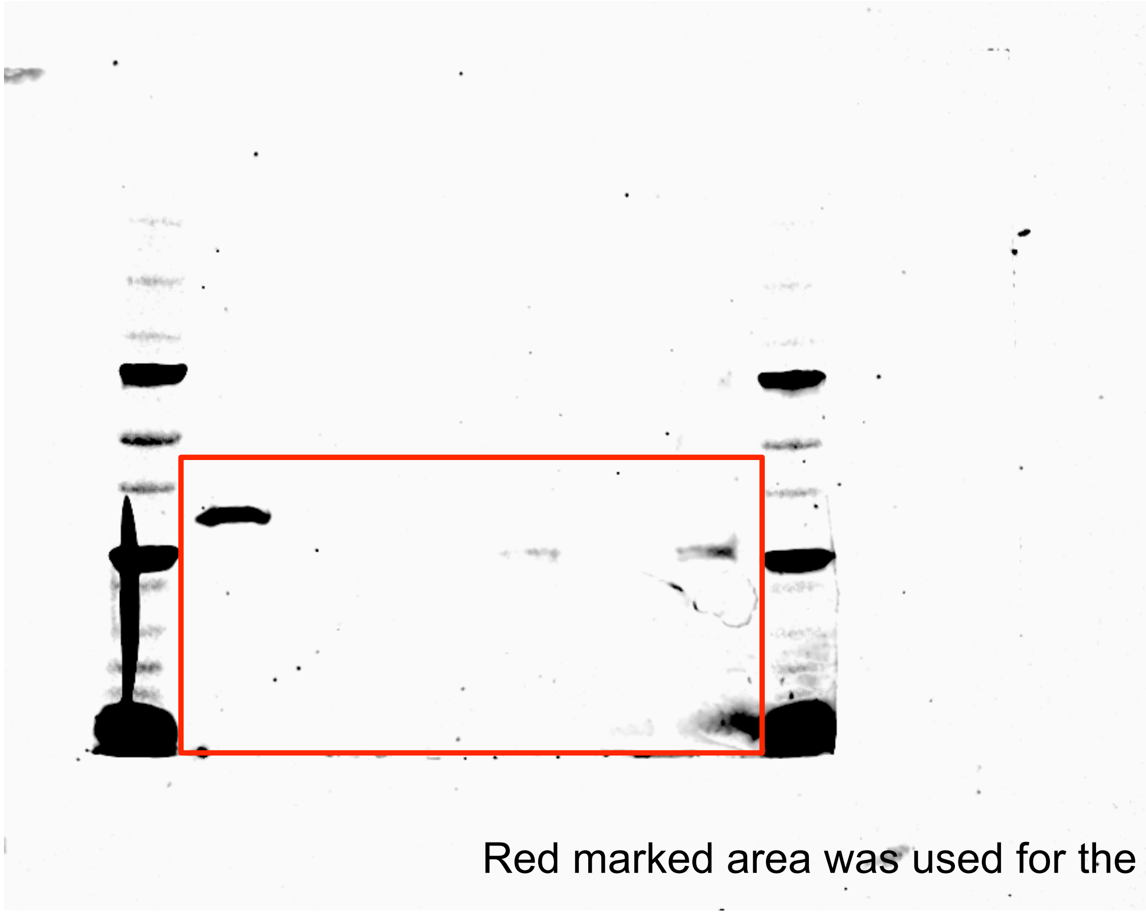

Red marked area was used for the figure.

Fig. 2. Phosphorylation of Thr143 and Thr149 in CycT1 contributes to its binding to CDK9

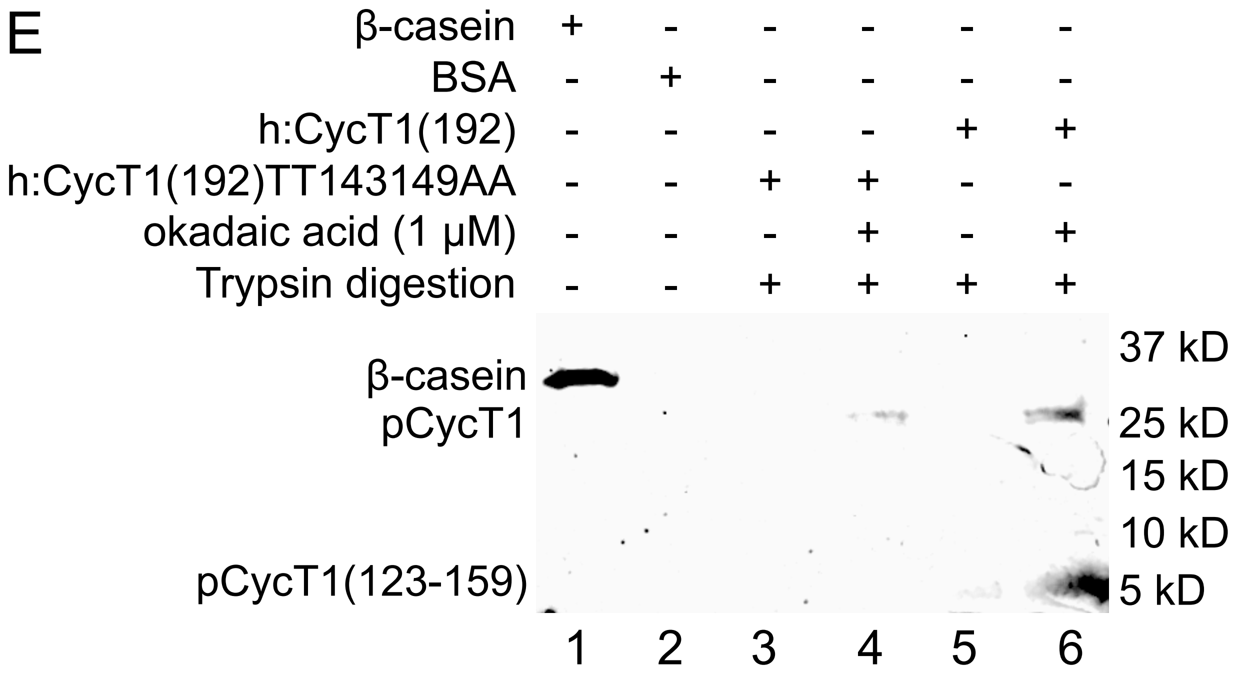

Fig.3. Phosphorylation of Thr143 and Thr149 stabilizes the interface between CycT1 and CDK9

A

Phosphorylated form

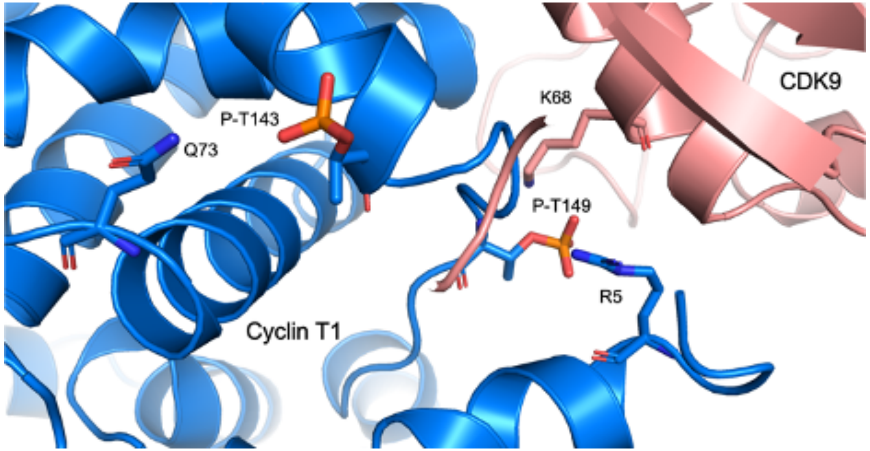

Source blot for Figure 3B

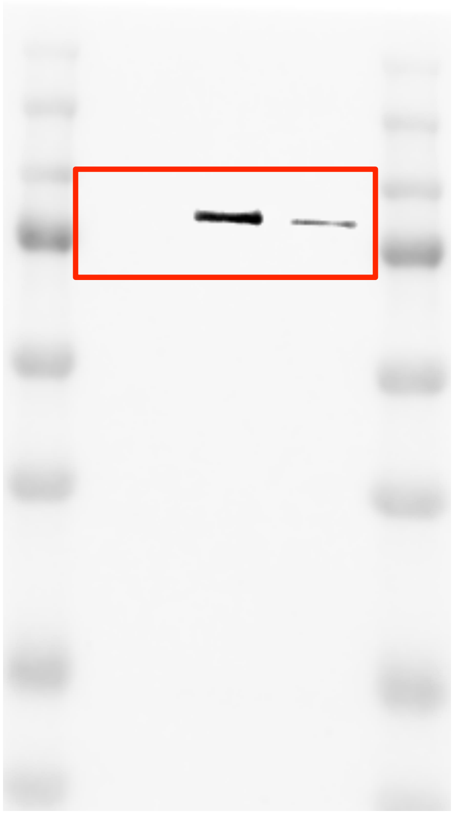

Red marked area was used for the figure.

Source blot for Figure 3B

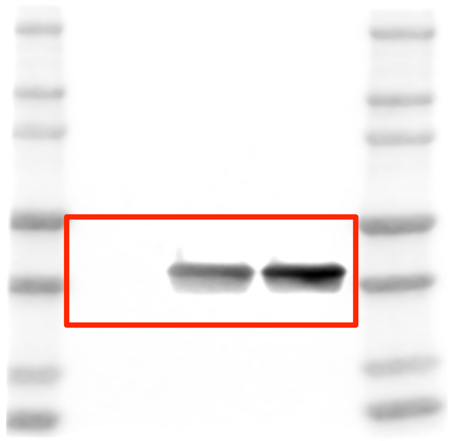

Red marked area was used for the figure.

Membrane was cut after transfer, and before probing by Ab

Source blot for Figure 3B

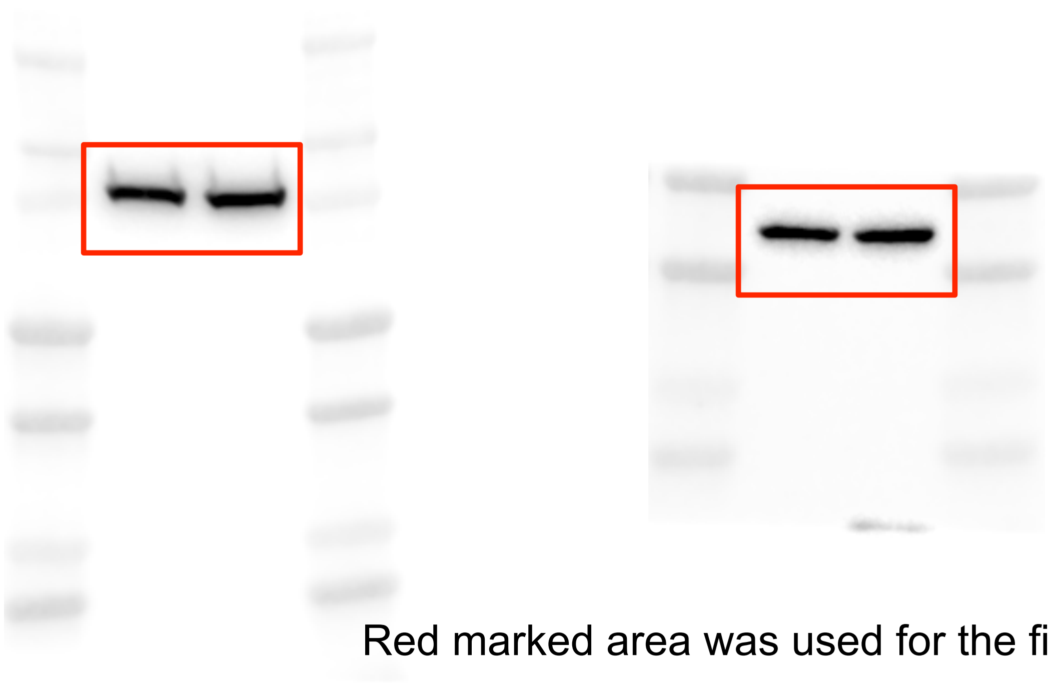

Red marked area was used for the figure.

Membrane was cut after transfer, and before probing by Ab
Two input proteins bolts are in the slide

Fig.3. Phosphorylation of Thr143 and Thr149 stabilizes the interface between CycT1 and CDK9

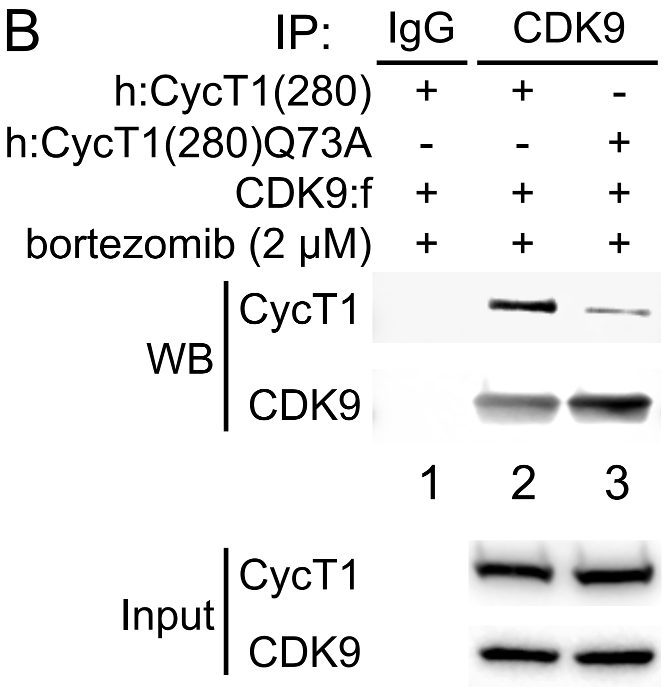

Source blot for Figure 3C

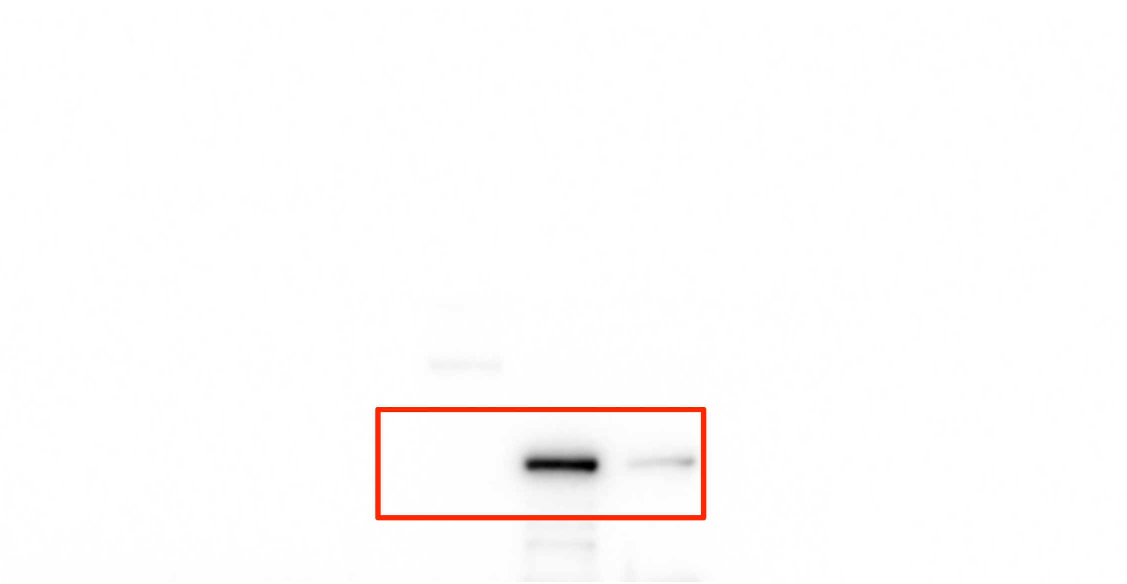

Red marked area was used for the figure.

Membrane was cut after transfer, and before probing by Ab

Source blot for Figure 3C

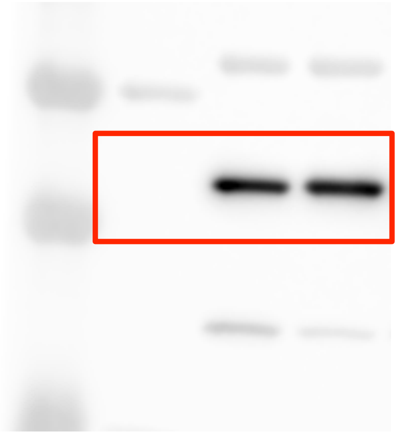

Red marked area was used for the figure.

Membrane was cut after transfer, and before probing by Ab

Source blot for Figure 3C

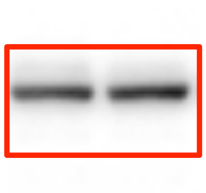

Red marked area was used for the figure.
Membrane was cut after transfer, and before probing by Ab

Source blot for Figure 3C

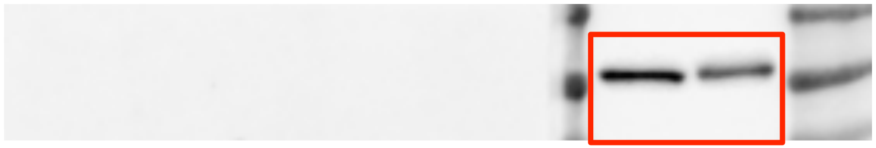

Red marked area was used for the figure.

Membrane was cut after transfer, and before probing by Ab

Fig.3. Phosphorylation of Thr143 and Thr149 stabilizes the interface between CycT1 and CDK9

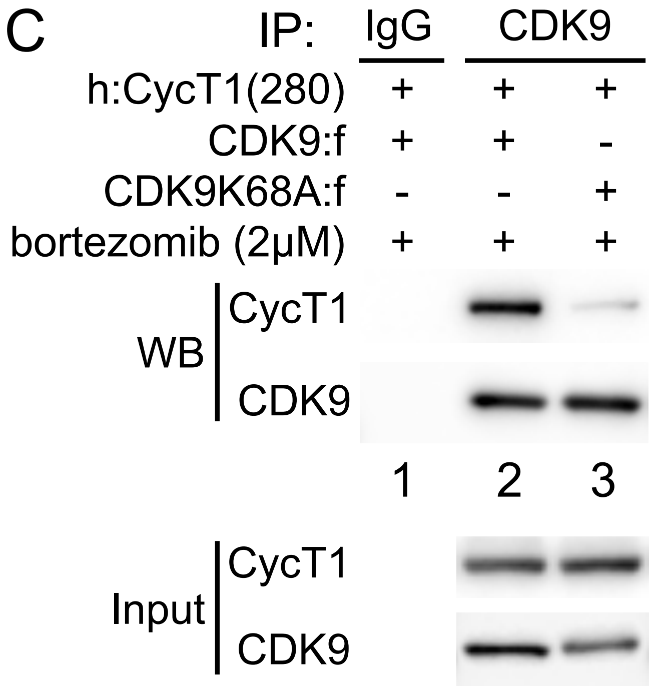

Source blot for Figure 3D

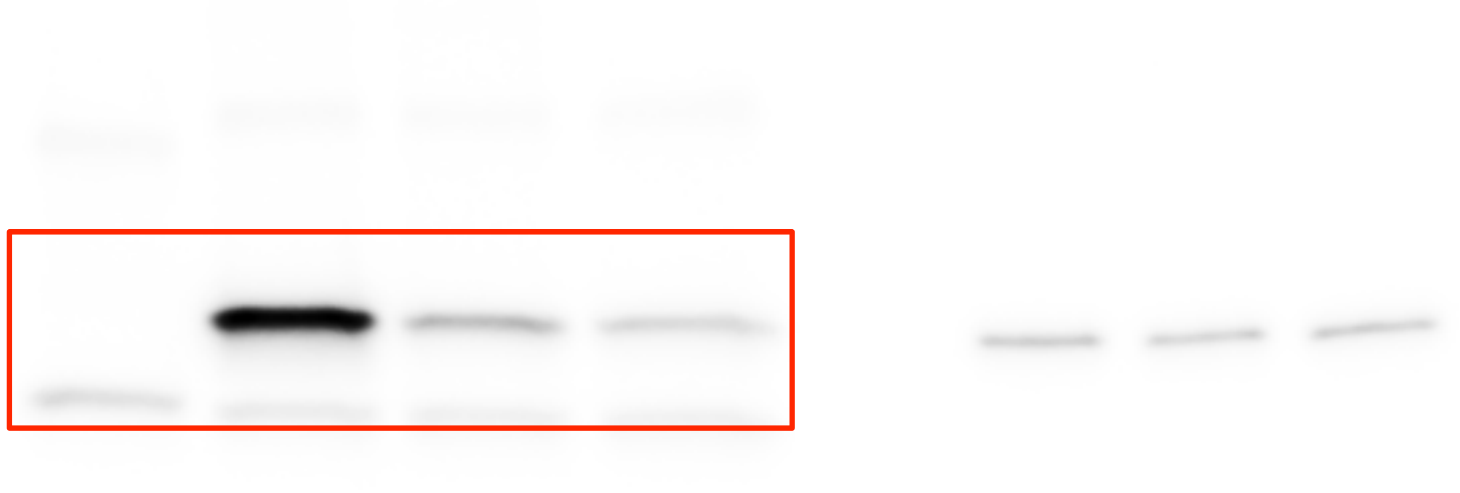

Red marked area was used for the figure.

Membrane was cut after transfer, and before probing by Ab
Right lanes are the same proteins under different conditions

Source blot for Figure 3D

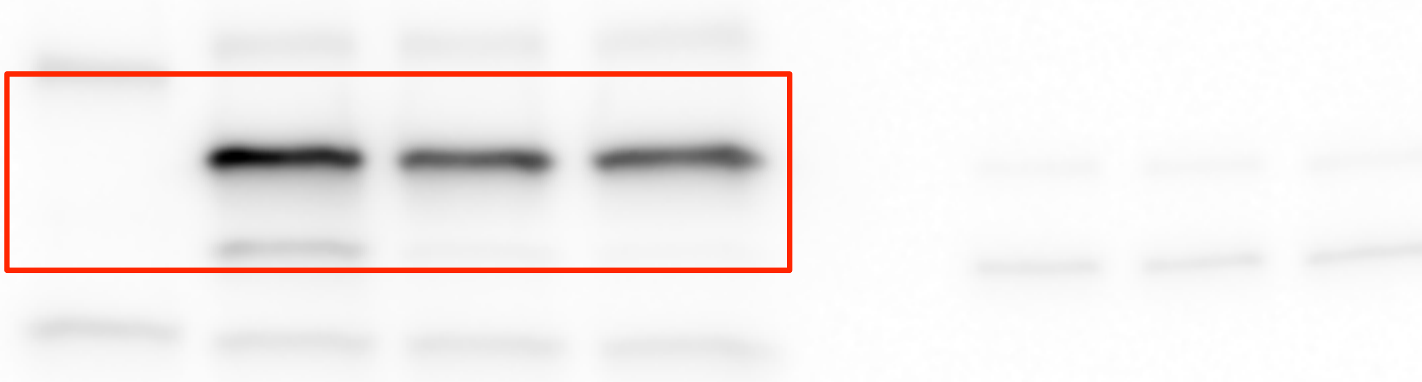

Red marked area was used for the figure.

Membrane was cut after transfer, and before probing by Ab
Right lanes are the other proteins under different conditions

Source blot for Figure 3D

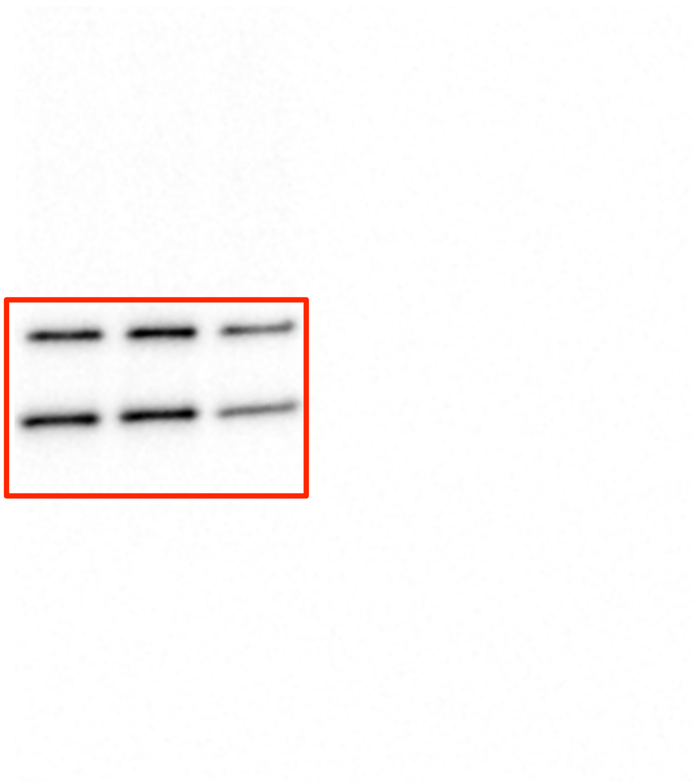

Red marked area was used for the figure.

Membrane was cut after transfer, and before probing by Ab
Two antibodies are used in the same time for input proteins

Fig.3. Phosphorylation of Thr143 and Thr149 stabilizes the interface between CycT1 and CDK9

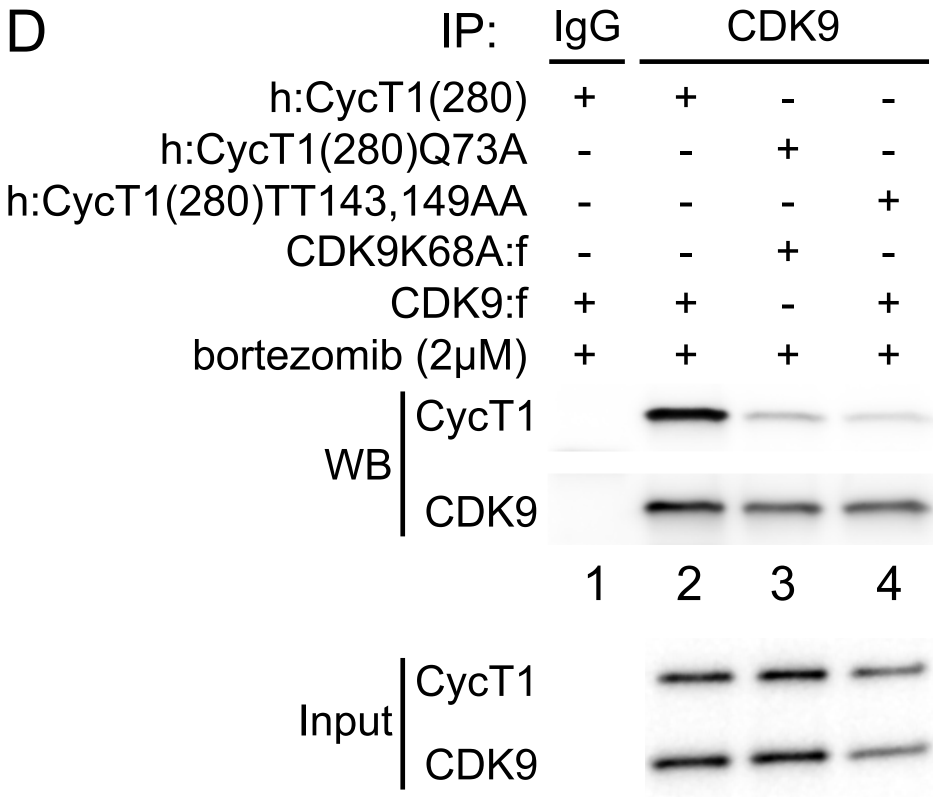

Source blot for Figure 4A

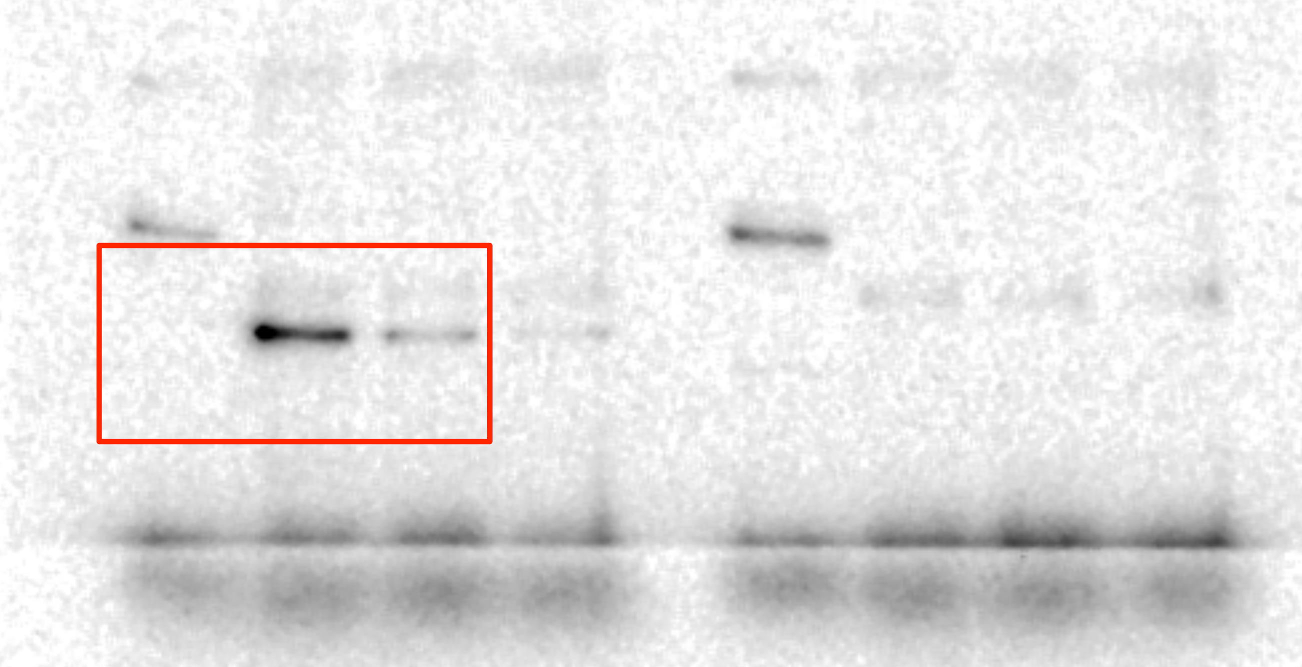

Red marked area was used for the figure.

The beside lane is the same protein with higher concentration of staurosporine

Source blot for Figure 4A

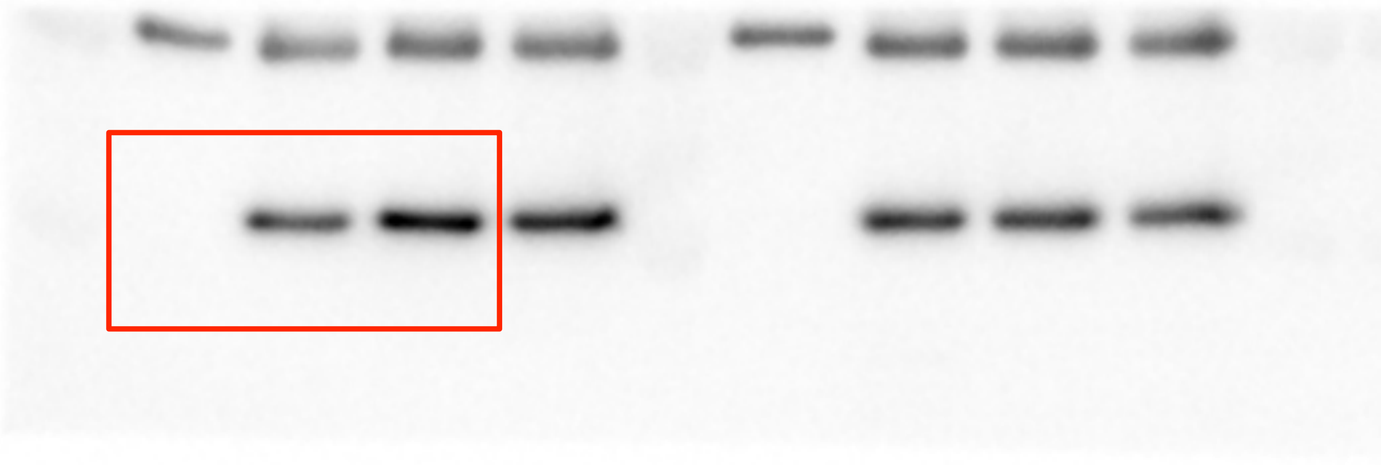

Red marked area was used for the figure.

The beside lane is the same protein with higher concentration of staurosporine
other lanes are the same protein under different conditions

Source blot for Figure 4A

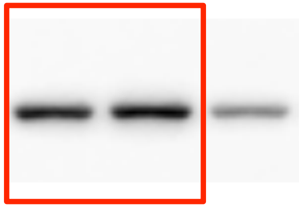

Red marked area was used for the figure.

Membrane was cut after transfer, and before probing by Ab
Right lane is the same protein with higher concentration of staurosporine

Source blot for Figure 4A

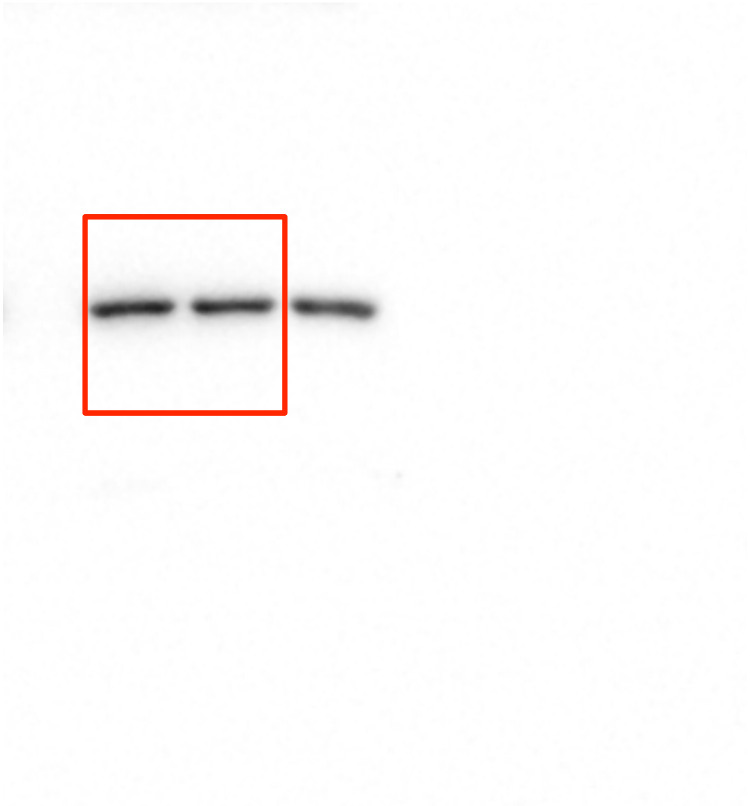

Red marked area was used for the figure.

Membrane was cut after transfer, and before probing by Ab
Right lane is the same protein with higher concentration of staurosporine

Fig. 4. PKC inhibitors impair interactions between CycT1 and CDK9, and promote CycT1 degradation

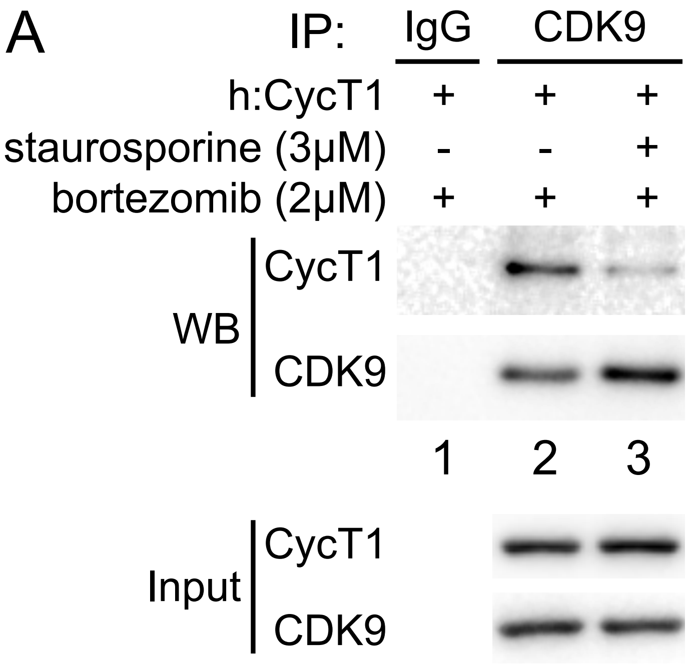

Source blot for Figure 4B

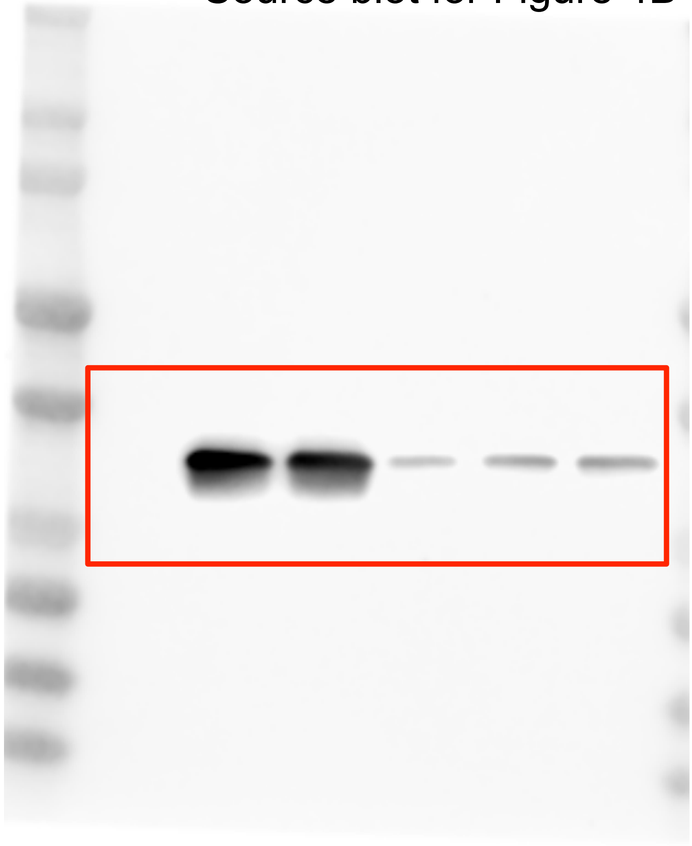

Red marked area was used for the figure.

Source blot for Figure 4B

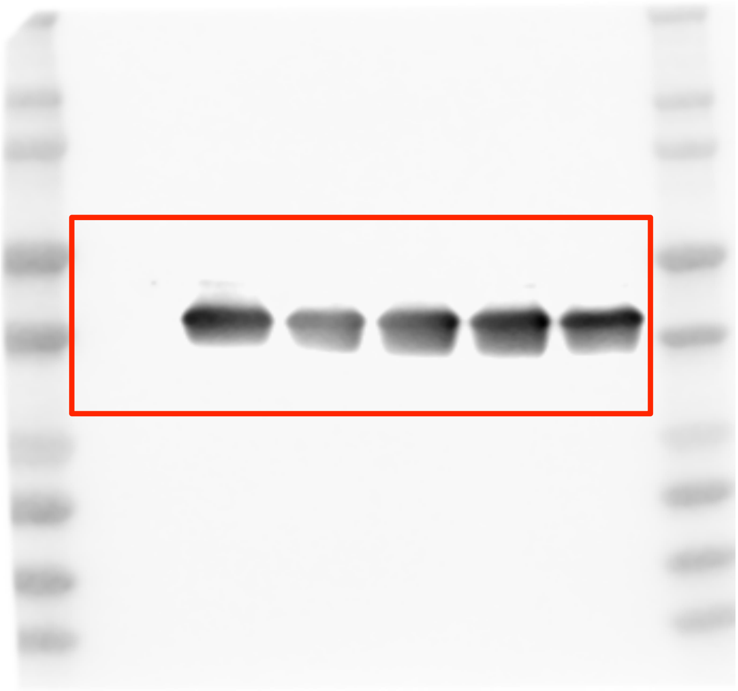

Red marked area was used for the figure.

Source blot for Figure 4B

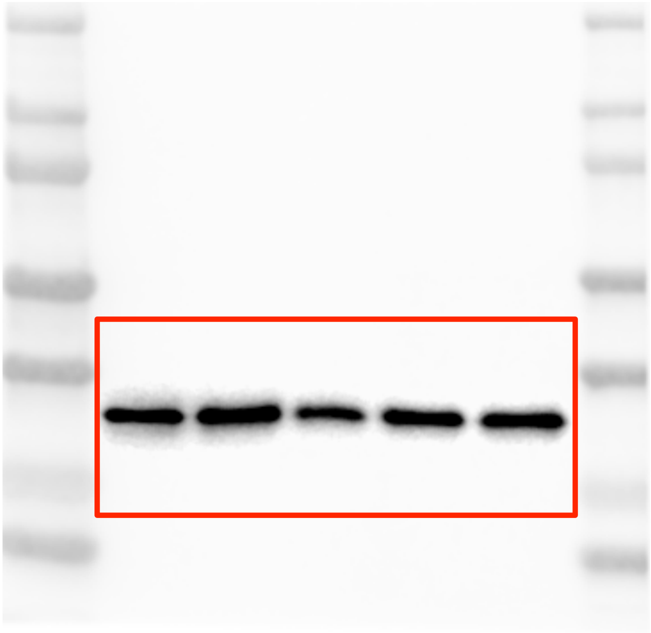

Red marked area was used for the figure.

Source blot for Figure 4B

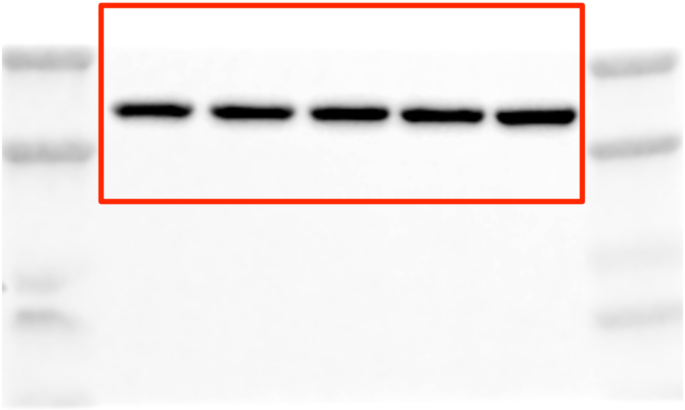

Red marked area was used for the figure.
Membrane was cut after transfer, and before probing by Ab

Fig. 4. PKC inhibitors impair interactions between CycT1 and CDK9, and promote CycT1 degradation

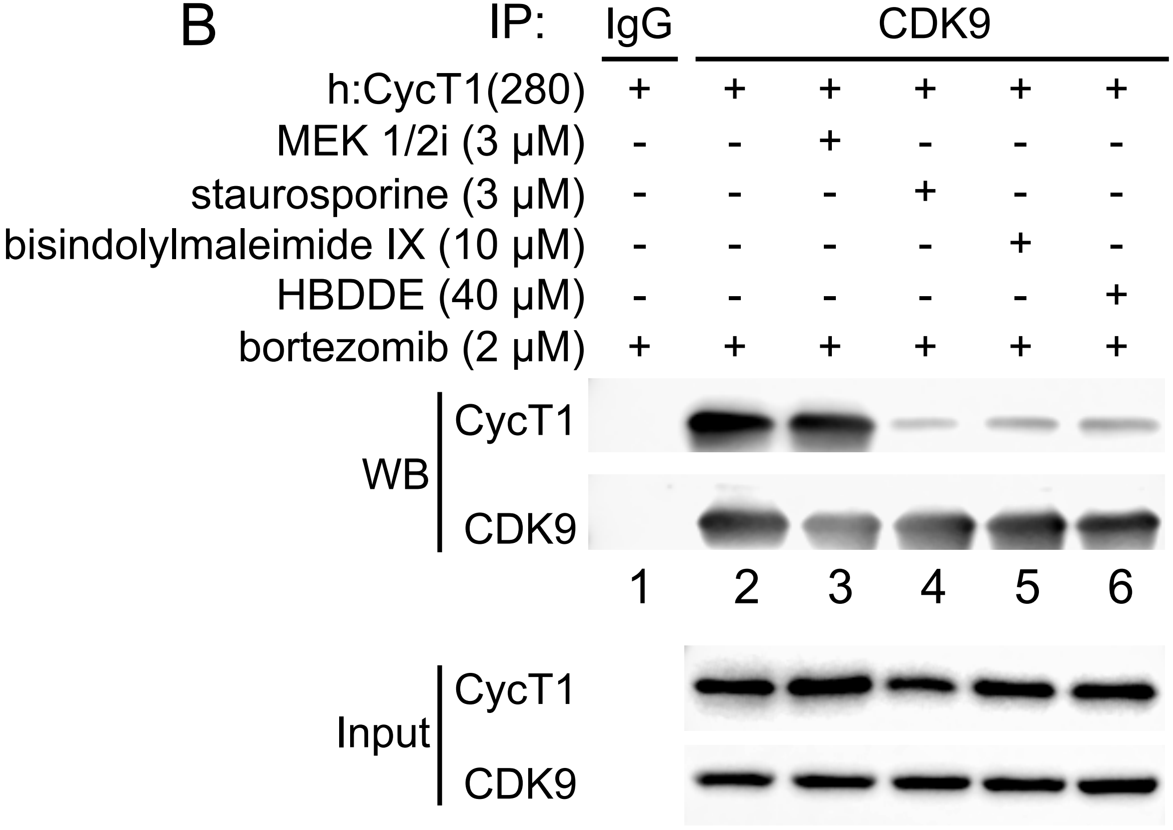

Source blot for Figure 4C

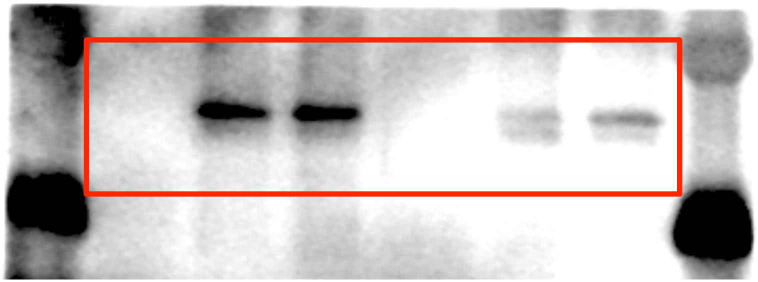

Red marked area was used for the figure.

Membrane was cut after transfer, and before probing by Ab

Source blot for Figure 4C

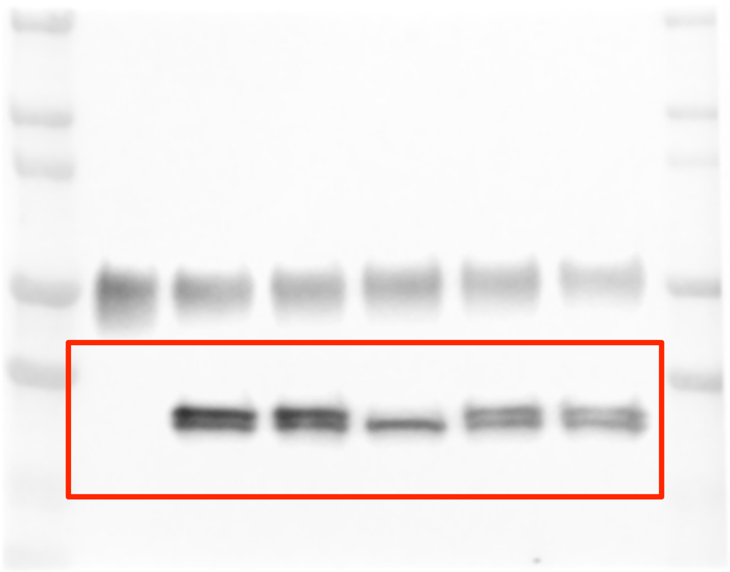

Red marked area was used for the figure.

Membrane was cut after transfer, and before probing by Ab
upper bands are heavy chain

Source blot for Figure 4C

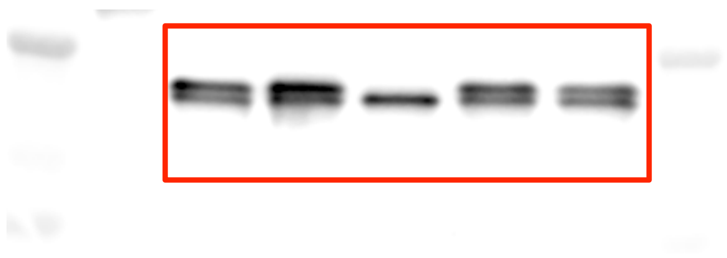

Red marked area was used for the figure.

Membrane was cut after transfer, and before probing by Ab

Fig. 4. PKC inhibitors impair interactions between CycT1 and CDK9, and promote CycT1 degradation

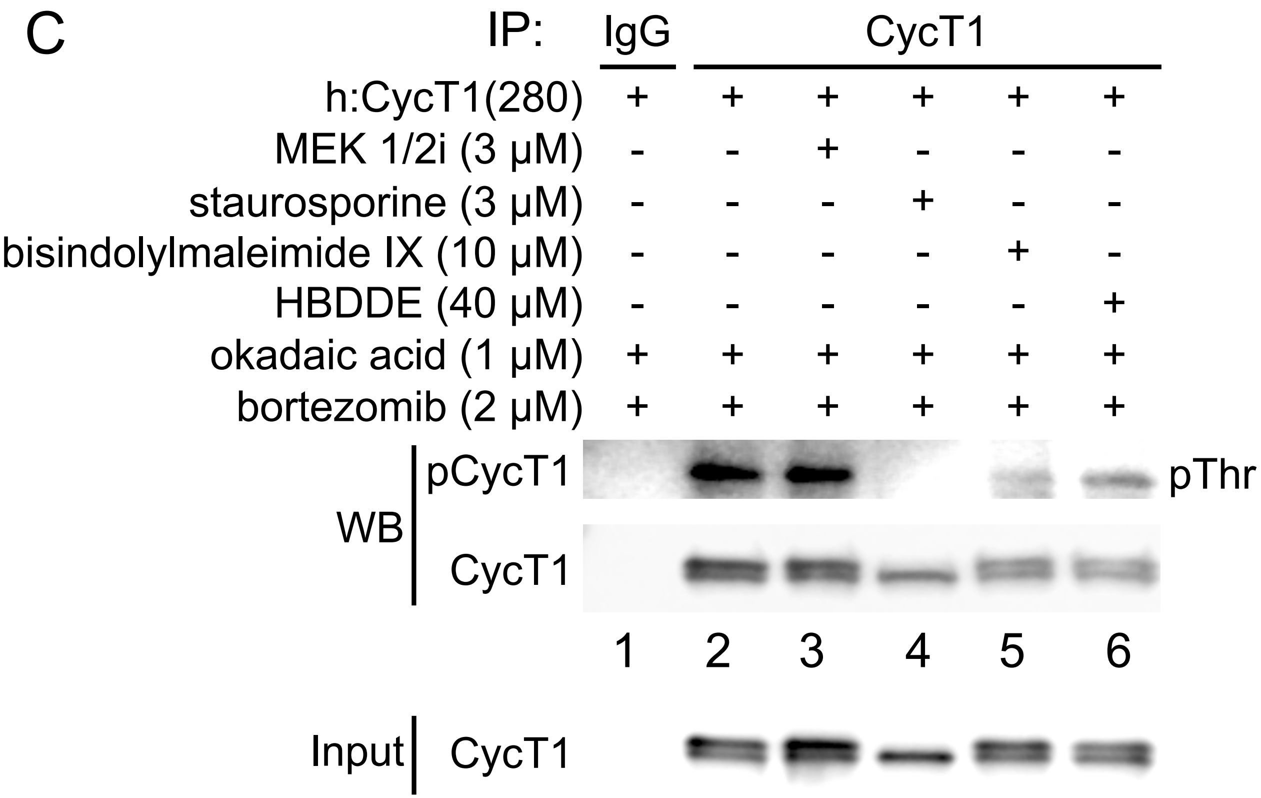

Source blot for Figure 4D

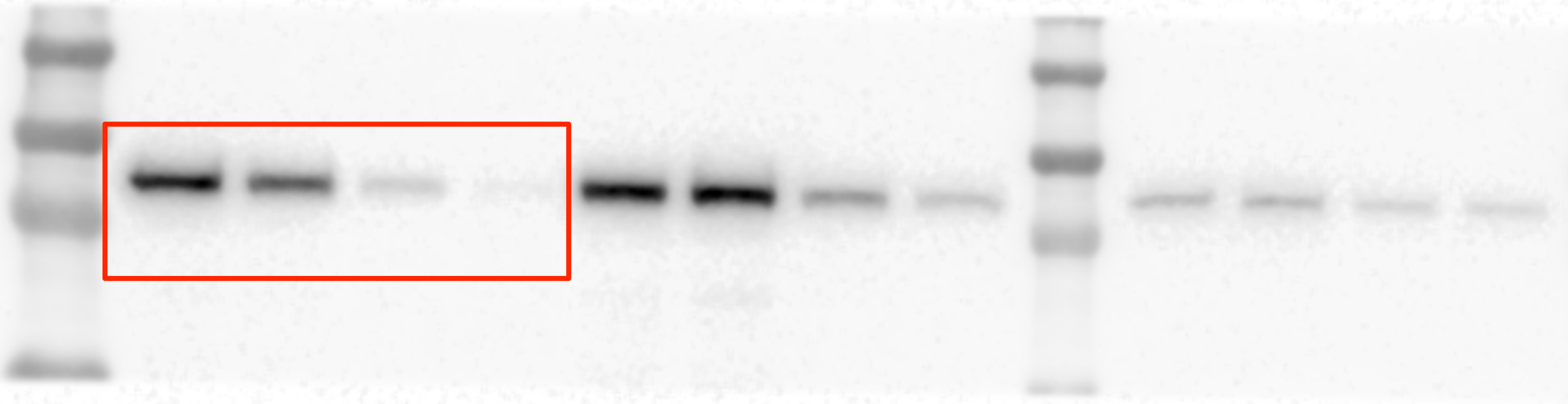

Red marked area was used for the figure.

Membrane was cut after transfer, and before probing by Ab
Right four lanes are for Figure 4E
Others are under different conditions

Source blot for Figure 4D

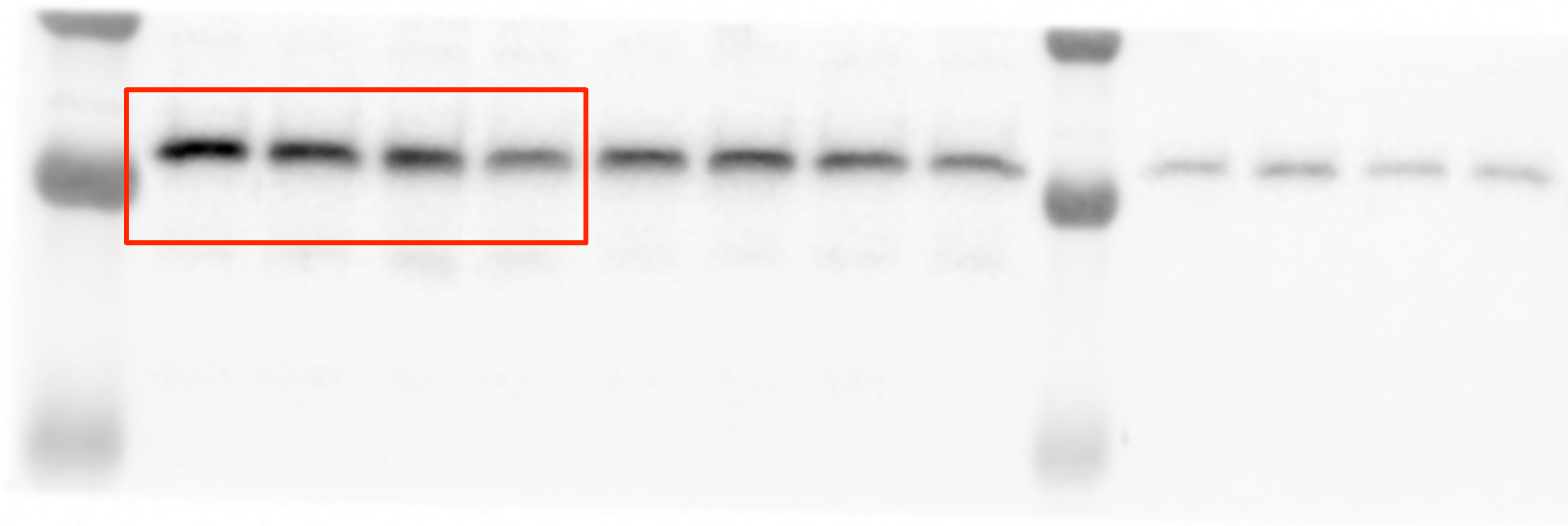

Red marked area was used for the figure.

Membrane was cut after transfer, and before probing by Ab
Right four lanes are for Figure 4E
Others are under different conditions

Source blot for Figure 4D

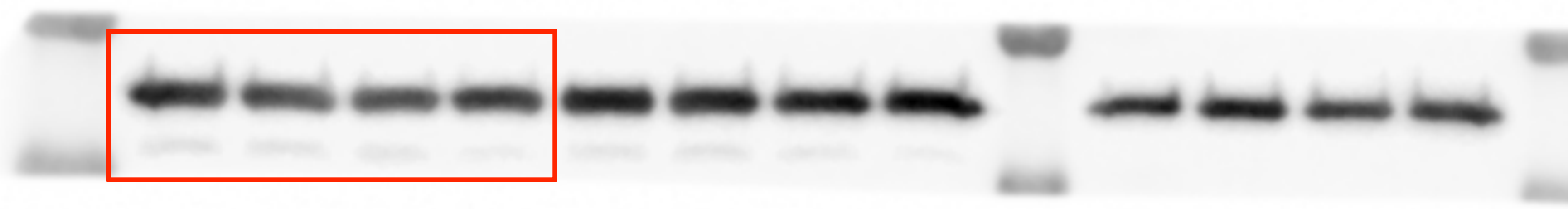

Red marked area was used for the figure.
Membrane was cut after transfer, and before probing by Ab
Right four lanes are for Figure 4E
Others are under different conditions

Fig. 4. PKC inhibitors impair interactions between CycT1 and CDK9, and promote CycT1 degradation

Jurkat cells

D

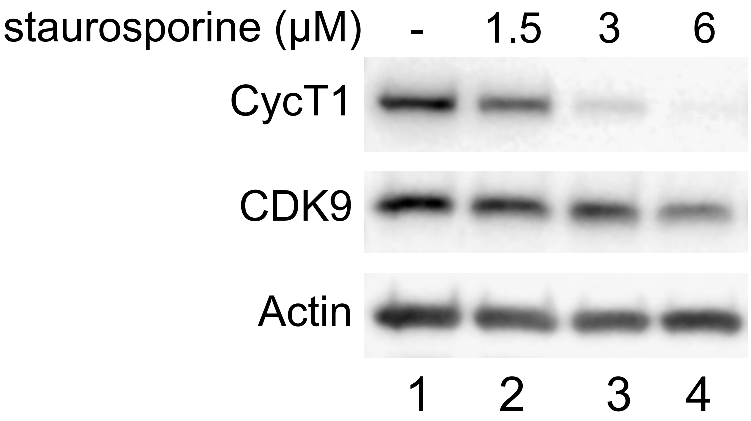

Source blot for Figure 4E

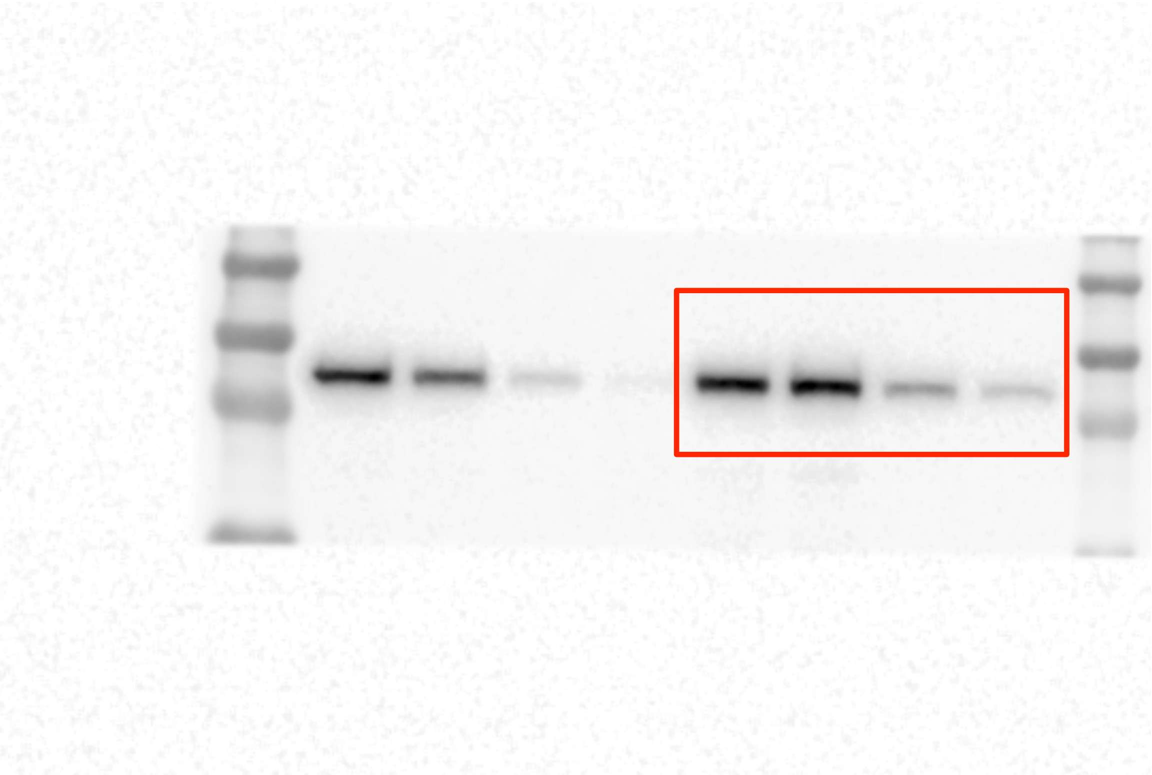

Red marked area was used for the figure.

Membrane was cut after transfer, and before probing by Ab
Left four lanes are for Figure 4D

Source blot for Figure 4E

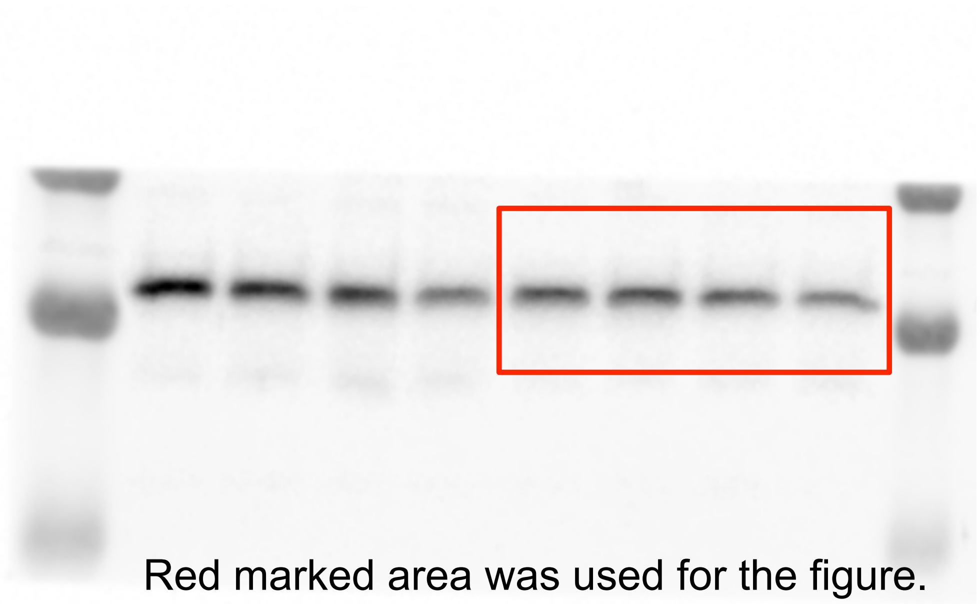

Red marked area was used for the figure.

Membrane was cut after transfer, and before probing by Ab
Left four lanes are for Figure 4D

Source blot for Figure 4E

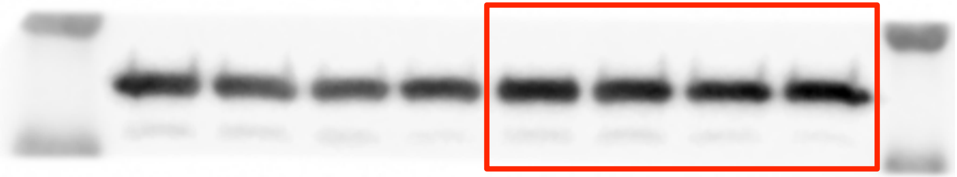

Red marked area was used for the figure.

Membrane was cut after transfer, and before probing by Ab
Left four lanes are for Figure 4D

Fig. 4. PKC inhibitors impair interactions between CycT1 and CDK9, and promote CycT1 degradation

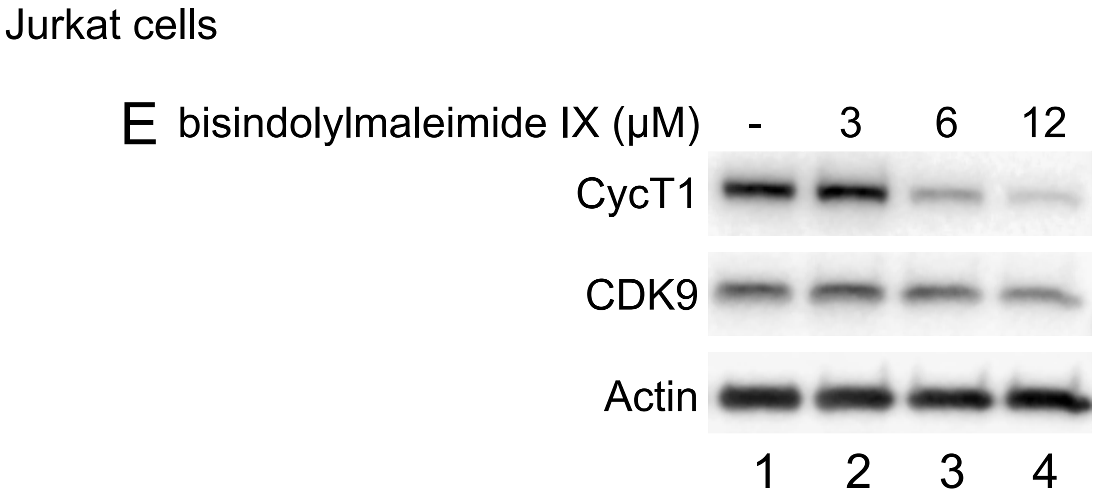

Source blot for Figure 4F

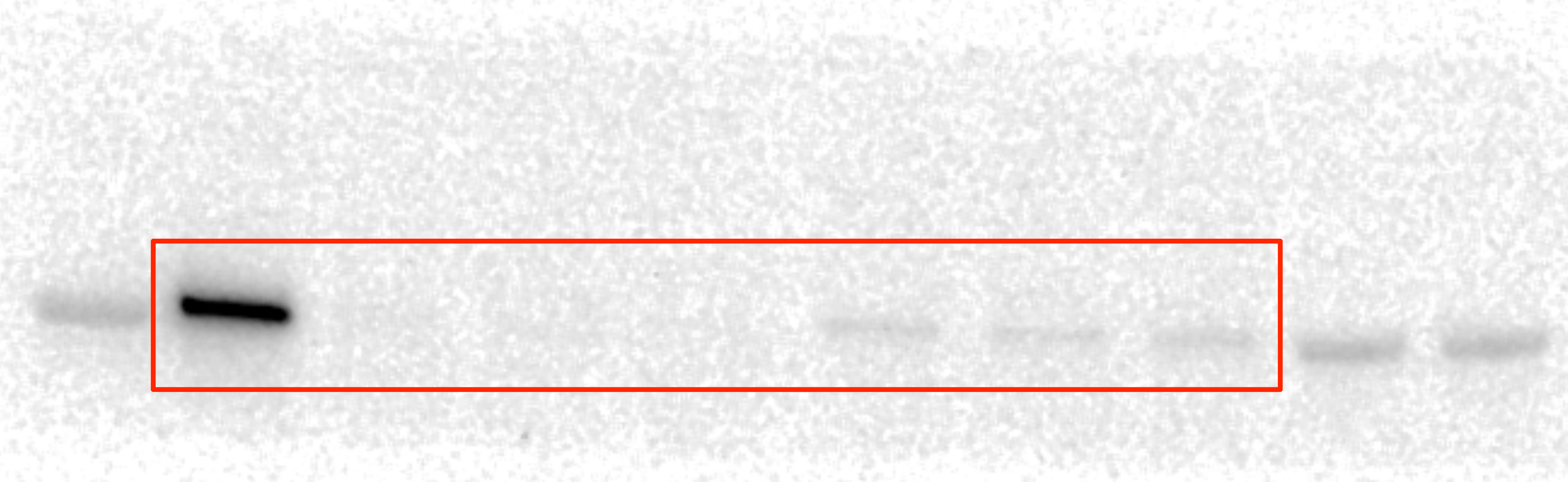

Red marked area was used for the figure.
Membrane was cut after transfer, and before probing by Ab

Source blot for Figure 4F

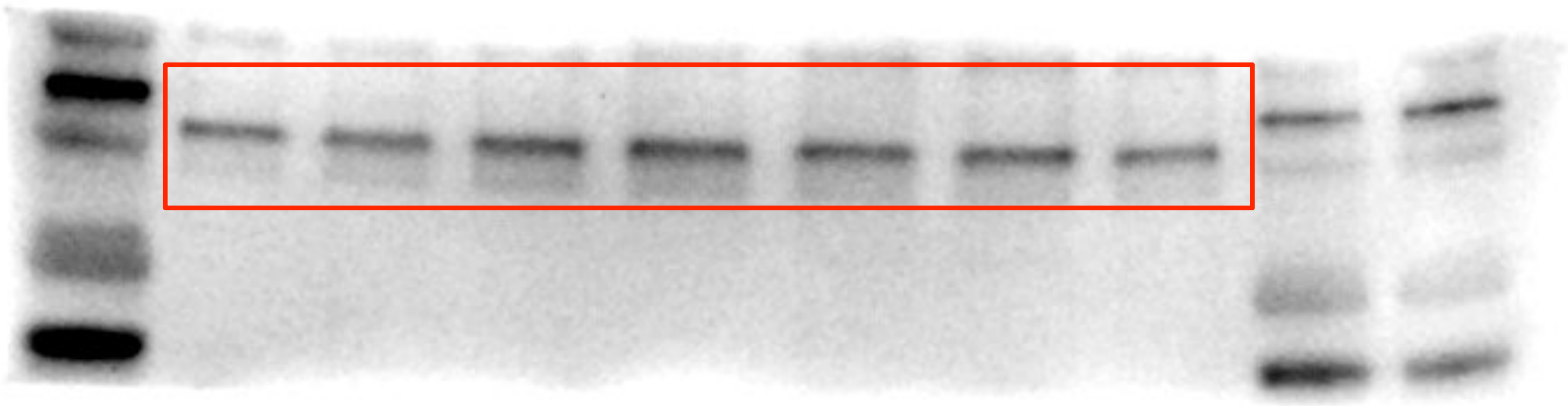

Red marked area was used for the figure.

Membrane was cut after transfer, and before probing by Ab

Source blot for Figure 4F

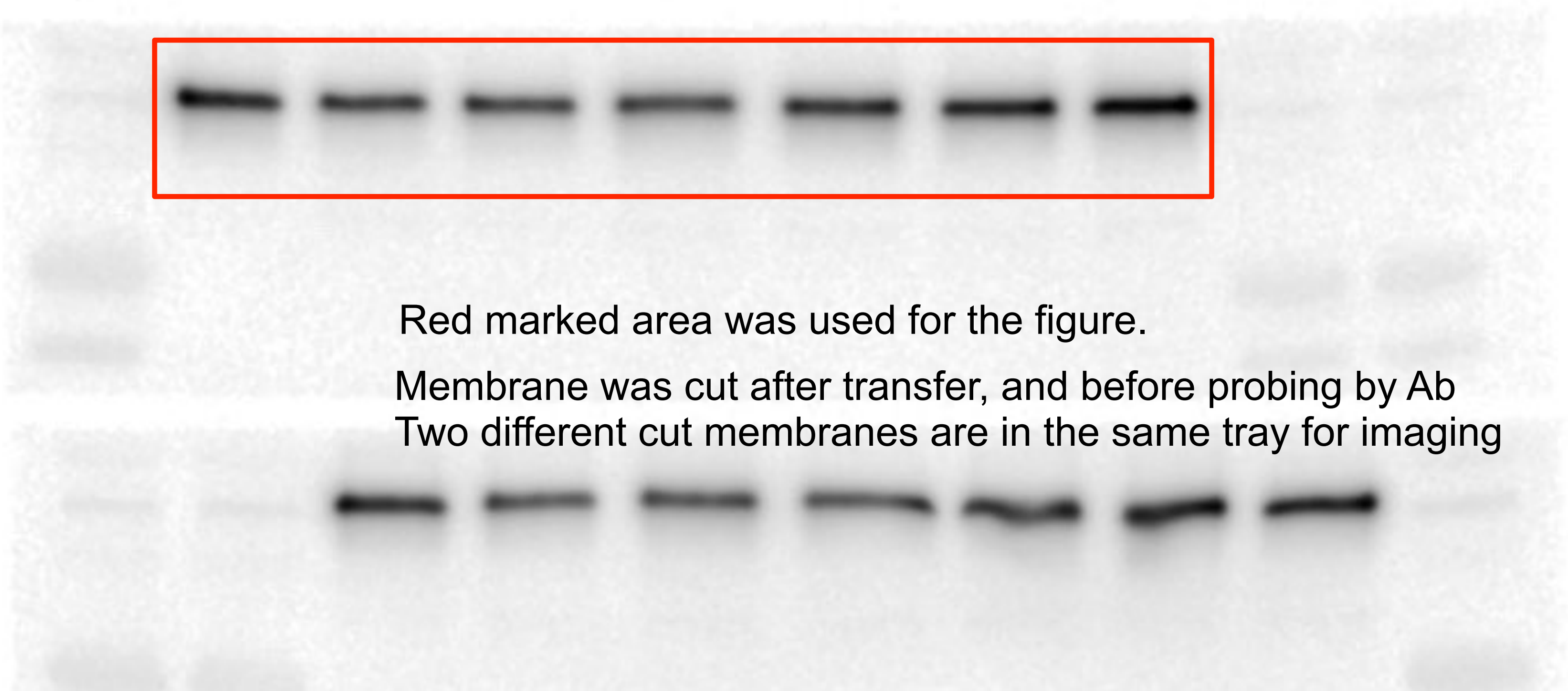

Fig. 4. PKC inhibitors impair interactions between CycT1 and CDK9, and promote CycT1 degradation

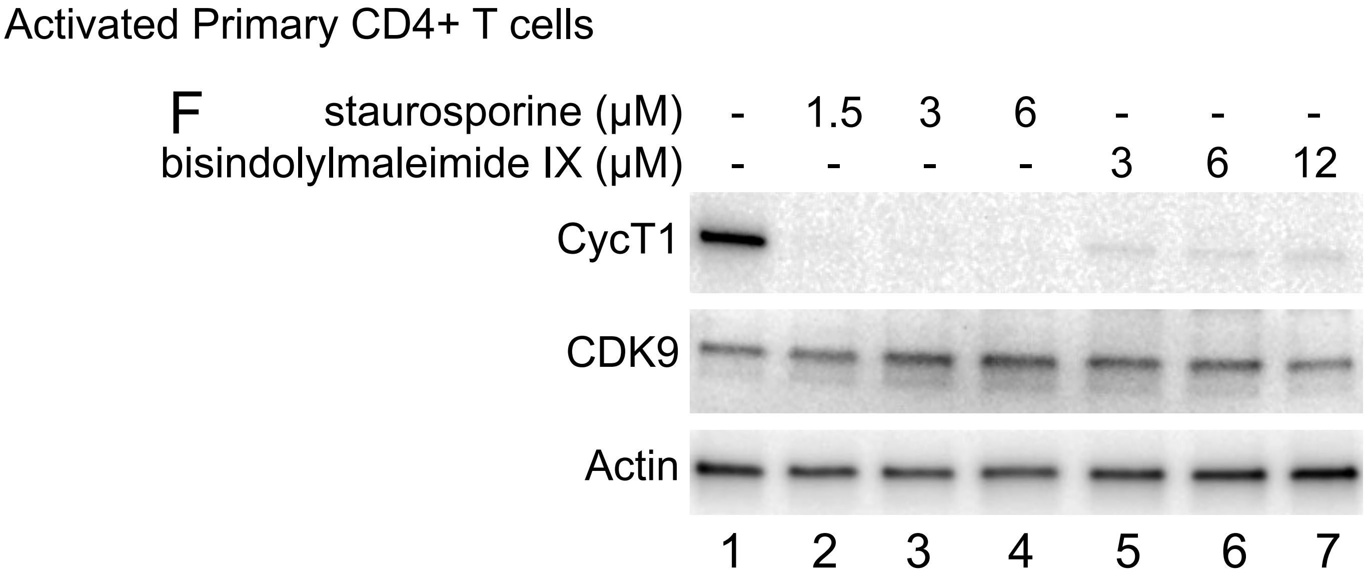

Source blot for Figure 5A

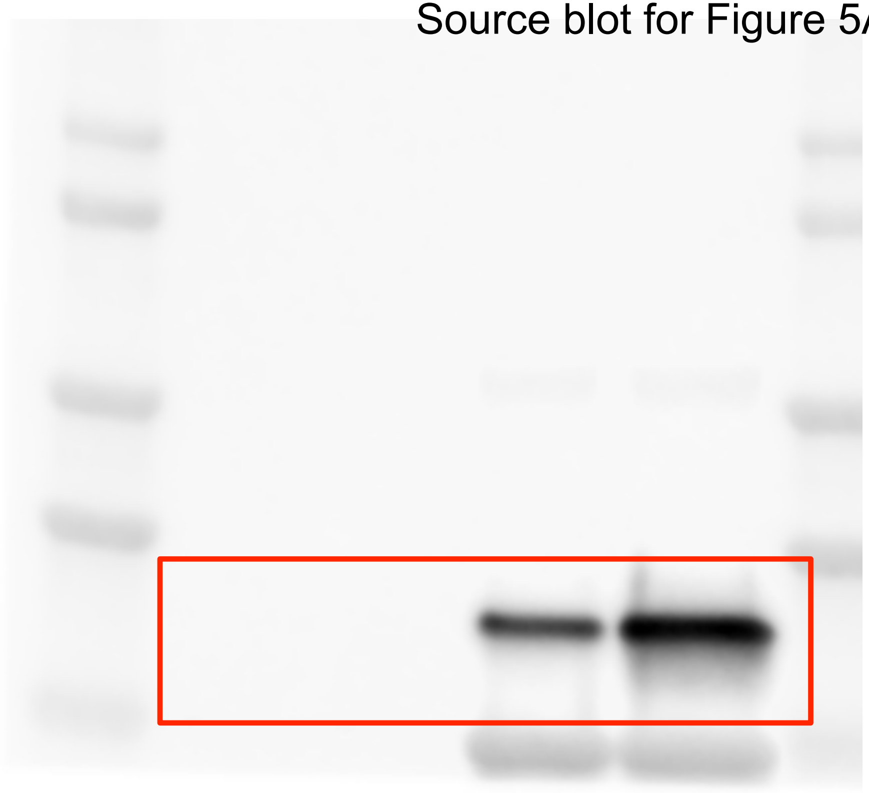

Red marked area was used for the figure.

Membrane was cut after transfer, and before probing by Ab

Source blot for Figure 5A

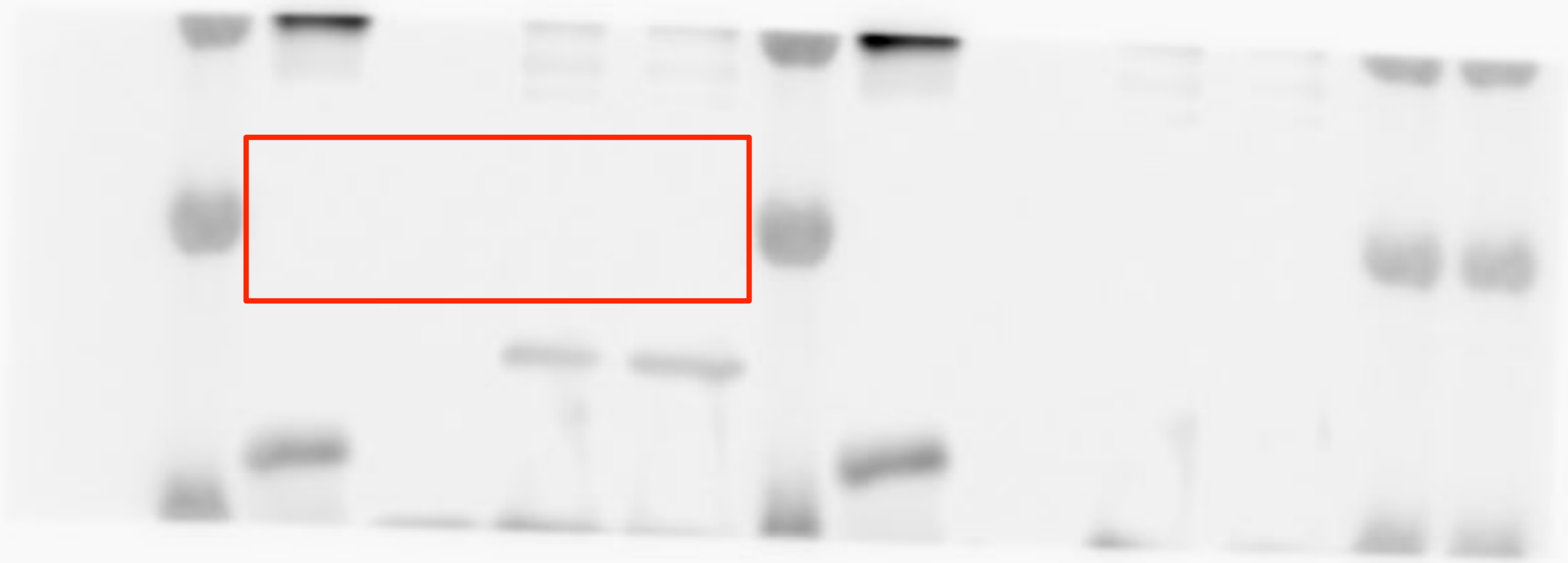

Red marked area was used for the figure.
Membrane was cut after transfer, and before probing by Ab

Source blot for Figure 5A

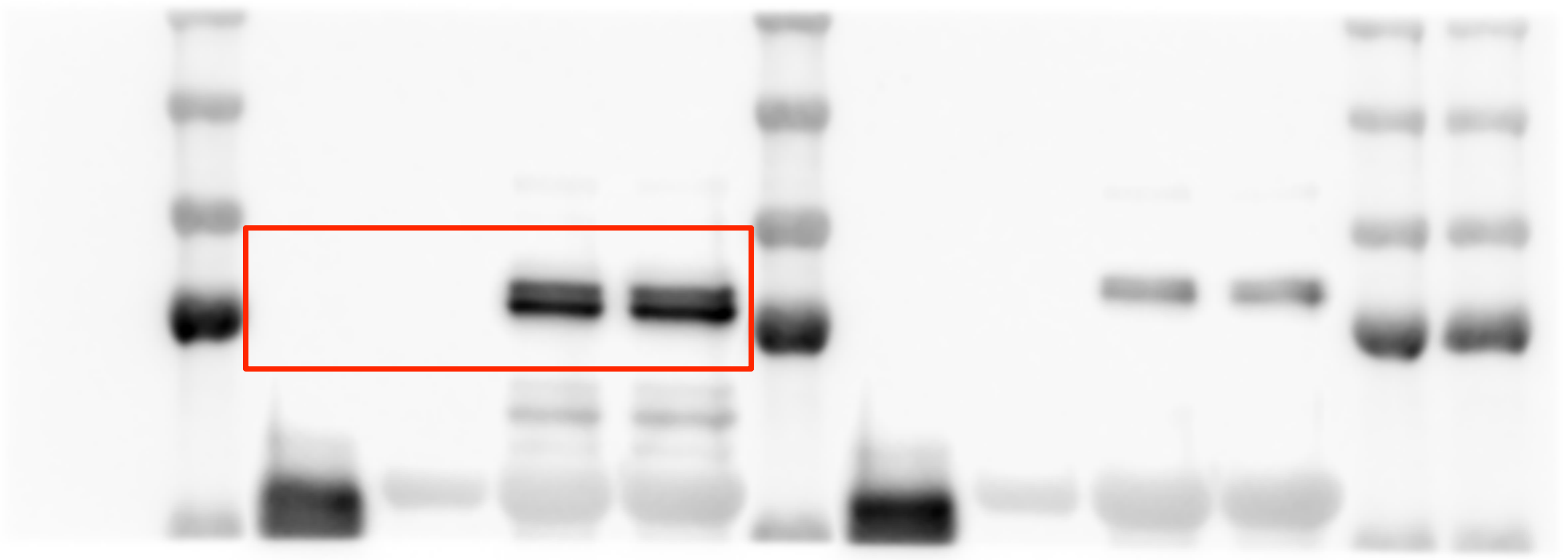

Red marked area was used for the figure.

Membrane was cut after transfer, and before probing by Ab
Right lanes are other proteins with same tag

Source blot for Figure 5A

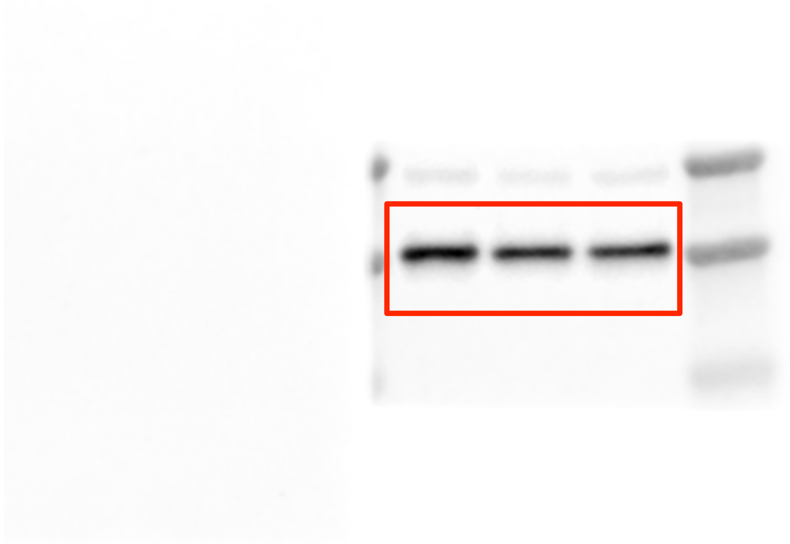

Red marked area was used for the figure.

Membrane was cut after transfer, and before probing by Ab

Source blot for Figure 5A

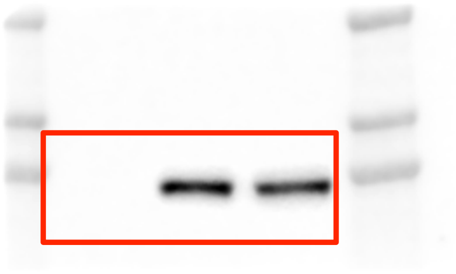

Red marked area was used for the figure.

Membrane was cut after transfer, and before probing by Ab

Fig. 5. PKCα and PKCβ bind to CycT1 for its phosphorylation, also promote interactions between CycT1 and CDK9, and increase the stability of CycT1

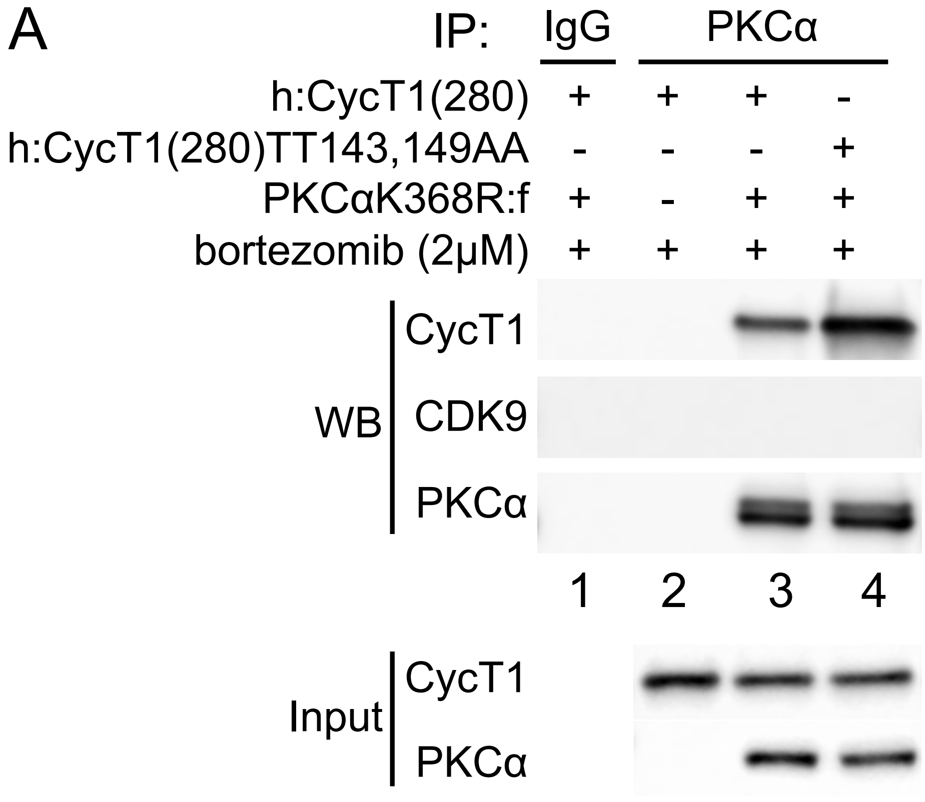

Source blot for Figure 5B

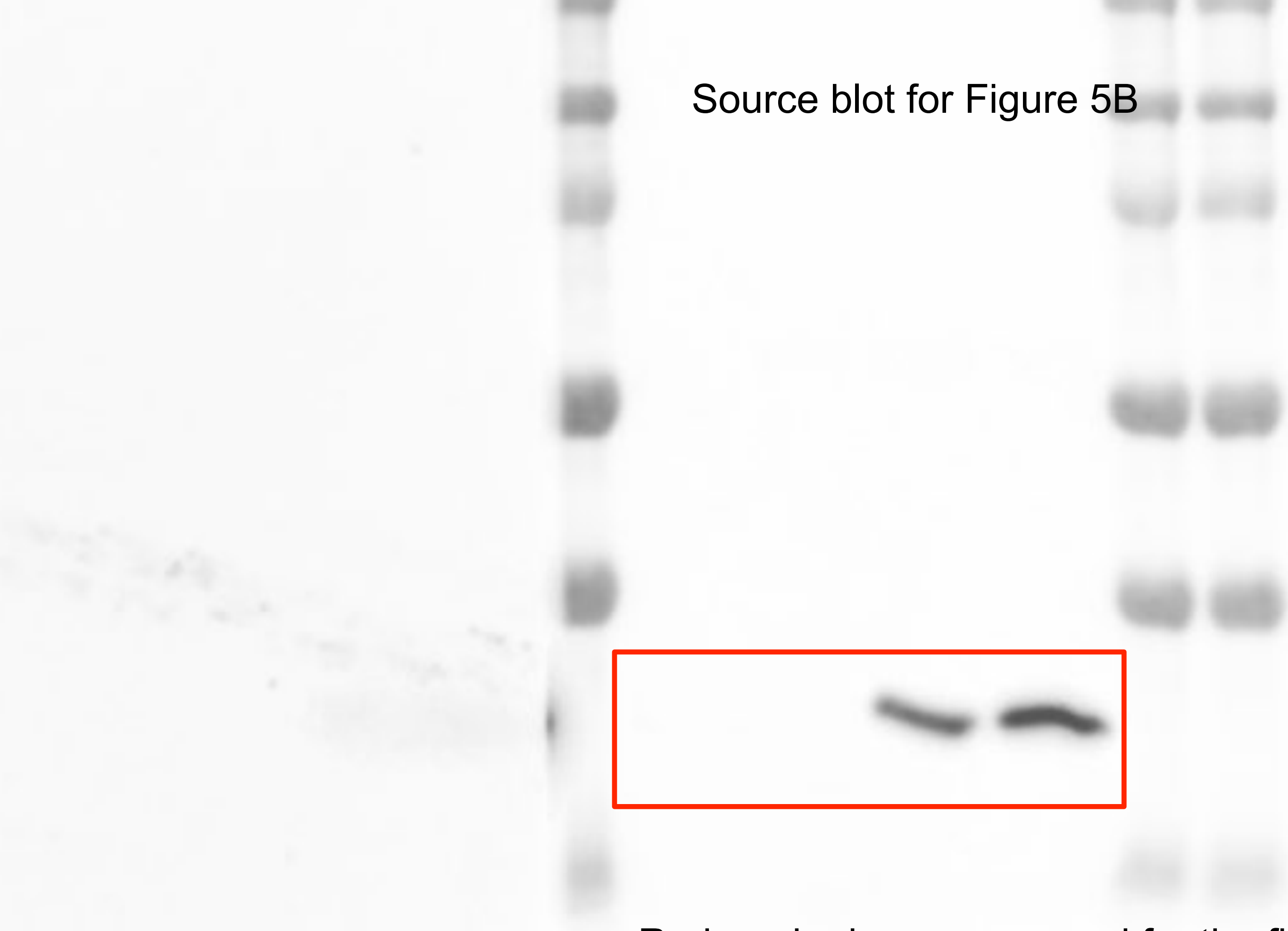

Red marked area was used for the figure.

Membrane was cut after transfer, and before probing by Ab

Source blot for Figure 5B

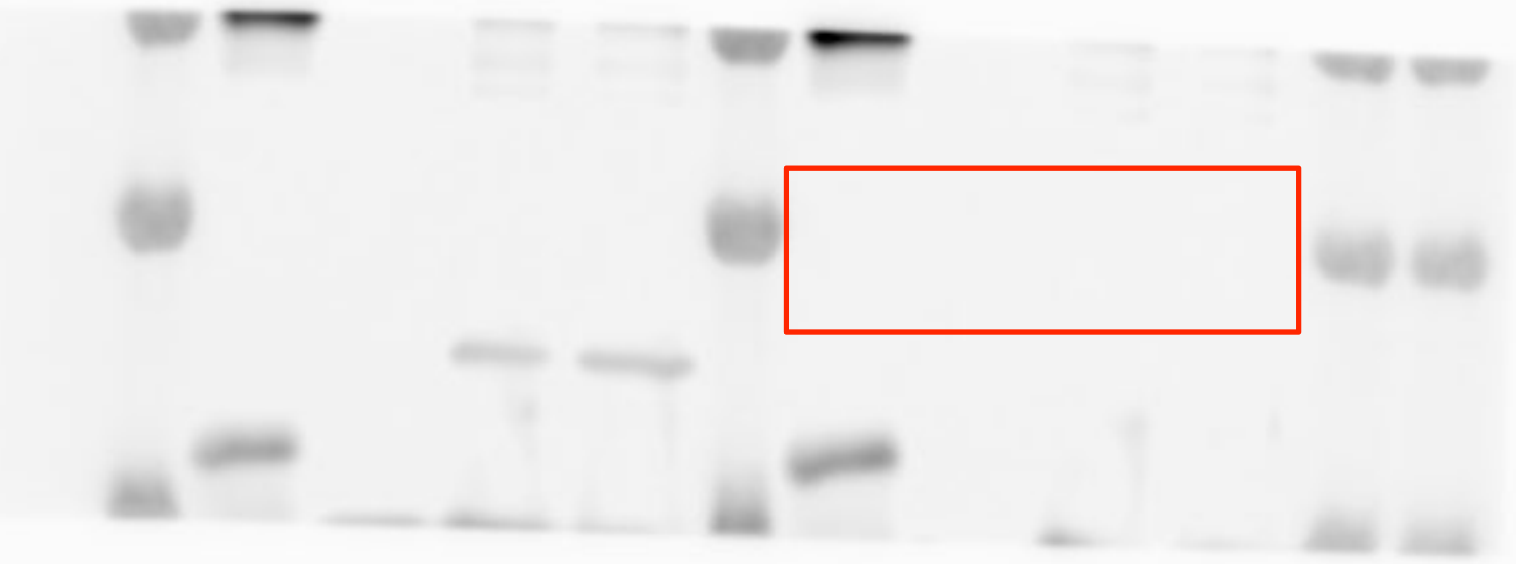

Red marked area was used for the figure.

Membrane was cut after transfer, and before probing by Ab

Source blot for Figure 5B

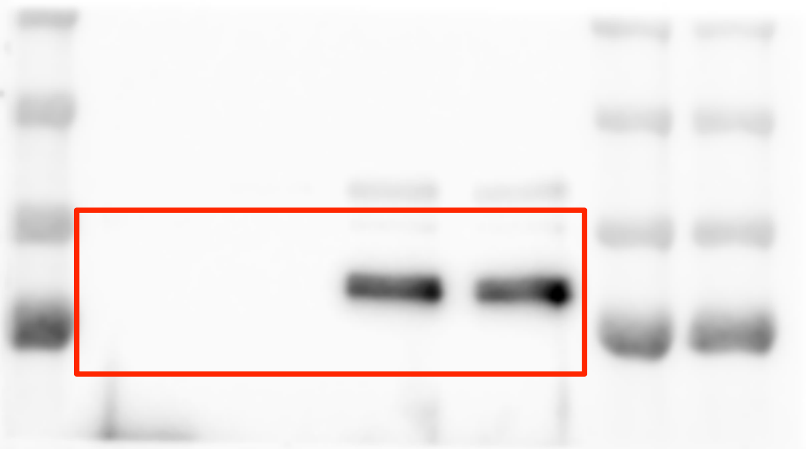

Red marked area was used for the figure.

Membrane was cut after transfer, and before probing by Ab

Source blot for Figure 5B

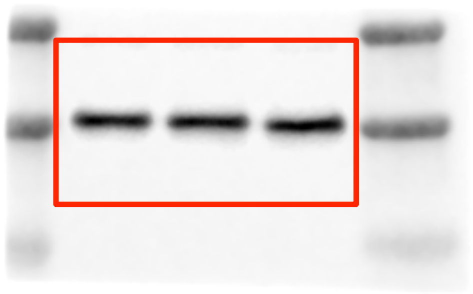

Red marked area was used for the figure.

Membrane was cut after transfer, and before probing by Ab

Source blot for Figure 5B

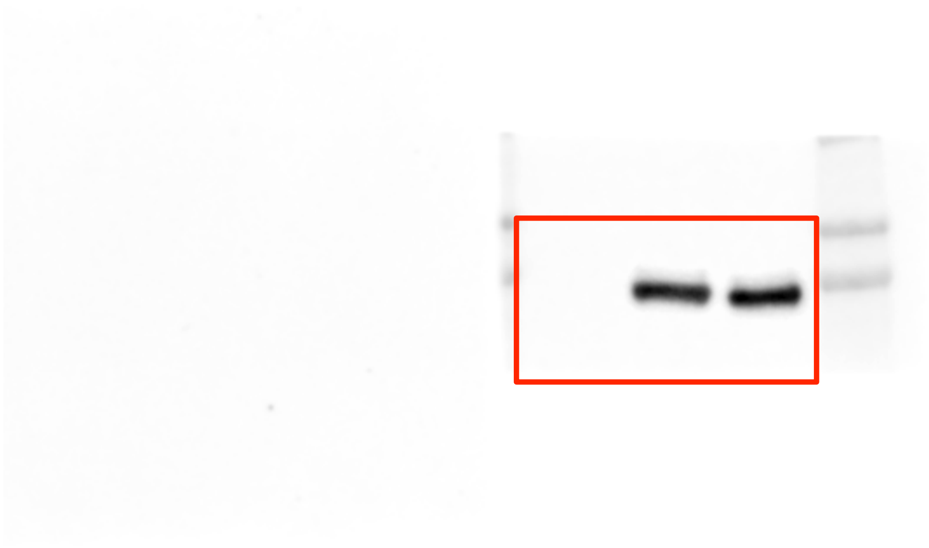

Red marked area was used for the figure.

Membrane was cut after transfer, and before probing by Ab

Fig. 5. PKCα and PKCβ bind to CycT1 for its phosphorylation, also promote interactions between CycT1 and CDK9, and increase the stability of CycT1

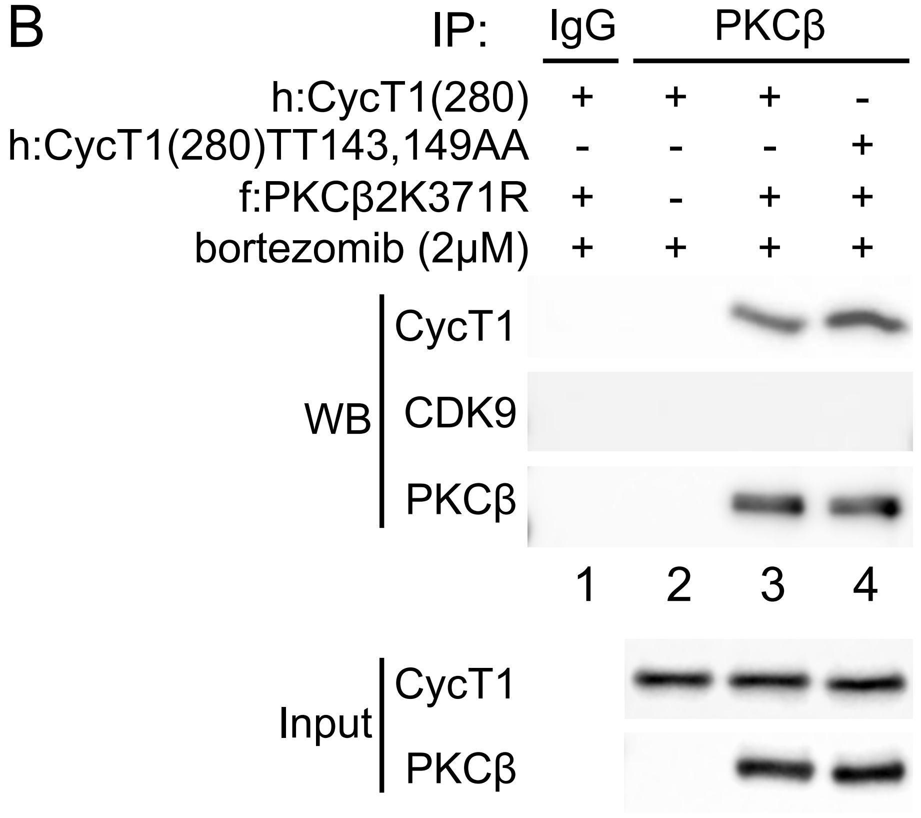

Source blot for Figure 5C

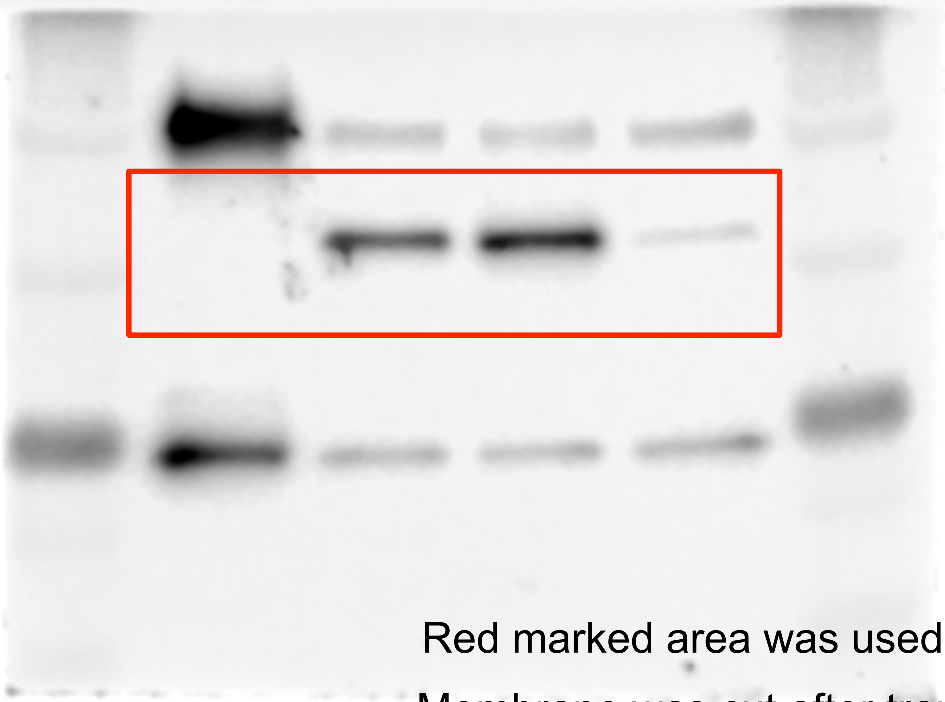

Red marked area was used for the figure.

Membrane was cut after transfer, and before probing by Ab
upper and lower bands are heavy chains and light chains

Source blot for Figure 5C

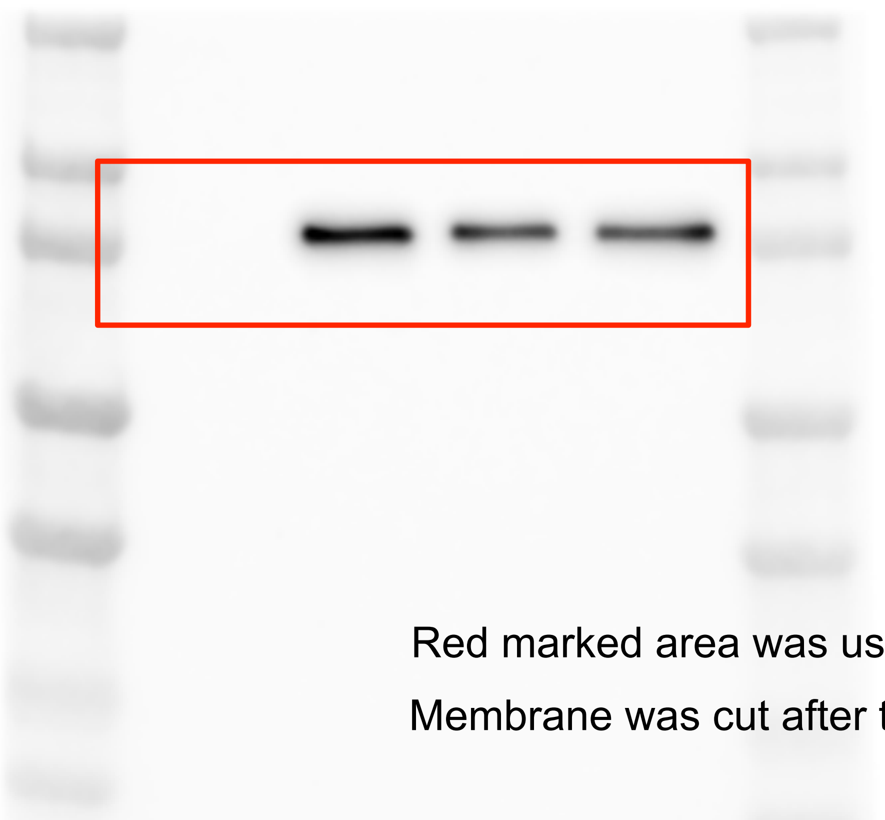

Red marked area was used for the figure.

Membrane was cut after transfer, and before probing by Ab

Source blot for Figure 5C

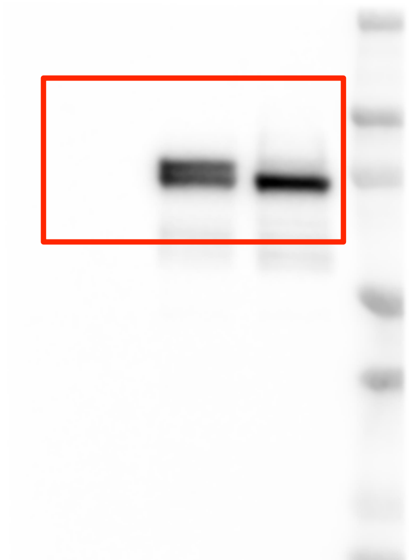

Red marked area was used for the figure.

Membrane was cut after transfer, and before probing by Ab

Source blot for Figure 5C

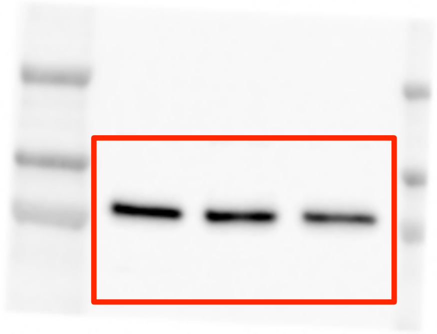

Red marked area was used for the figure.

Membrane was cut after transfer, and before probing by Ab

Source blot for Figure 5C

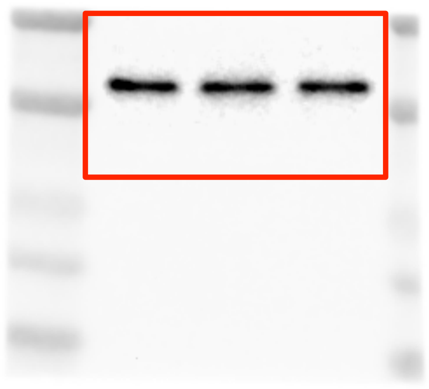

Red marked area was used for the figure.

Membrane was cut after transfer, and before probing by Ab

Fig. 5. PKCα and PKCβ bind to CycT1 for its phosphorylation, also promote interactions between CycT1 and CDK9, and increase the stability of CycT1

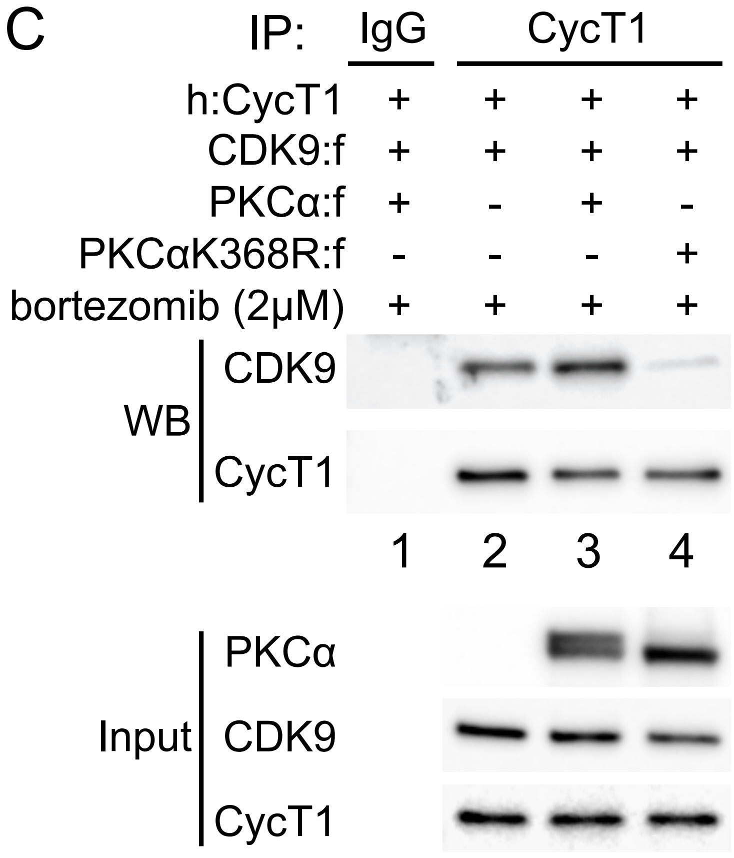

Source blot for Figure 5D

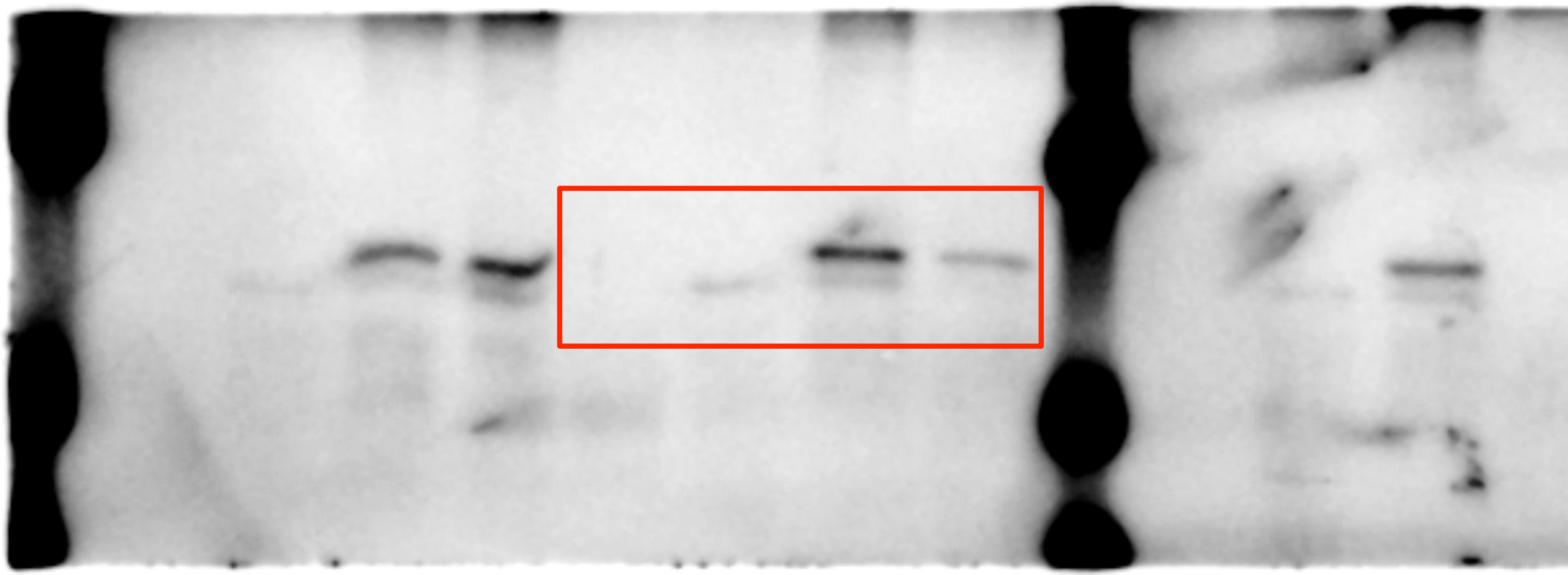

Red marked area was used for the figure.

Membrane was cut after transfer, and before probing by Ab
other bands are same proteins under different conditions

Source blot for Figure 5D

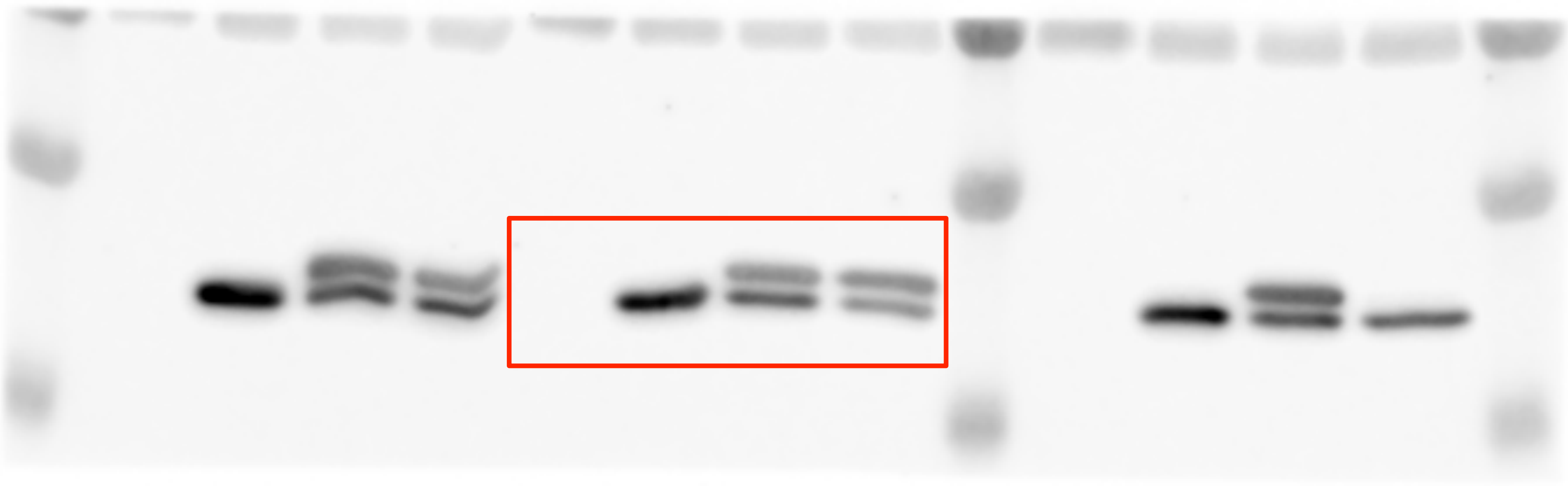

Red marked area was used for the figure.

Membrane was cut after transfer, and before probing by Ab
other bands are same proteins under different conditions

Source blot for Figure 5D

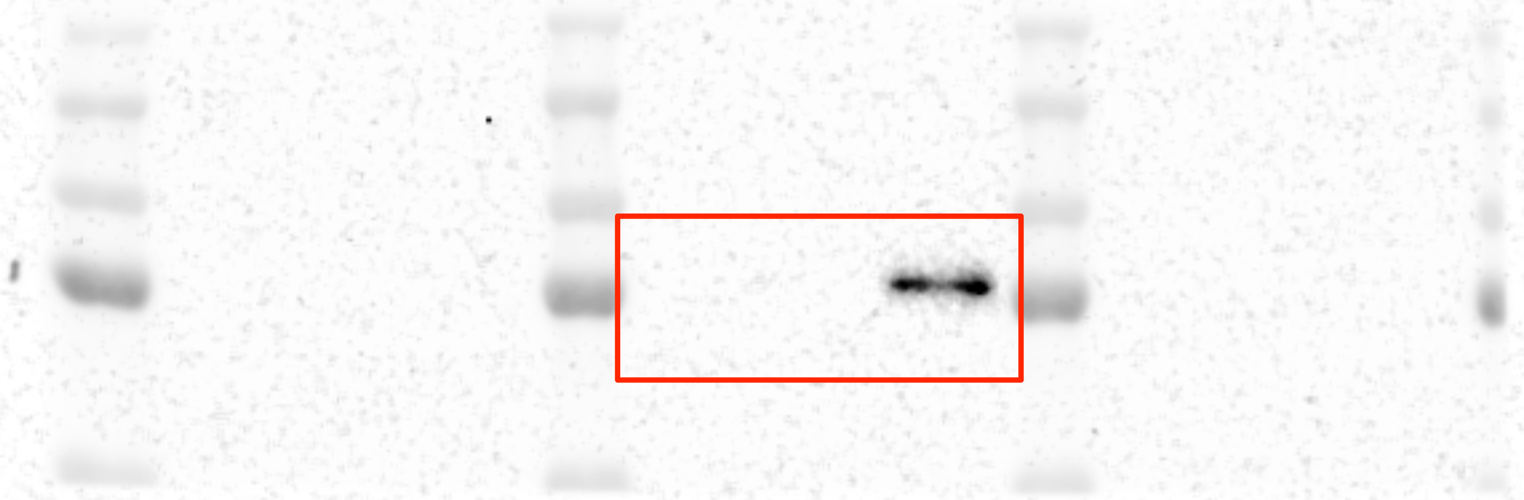

Red marked area was used for the figure.
Membrane was cut after transfer, and before probing by Ab

Source blot for Figure 5D

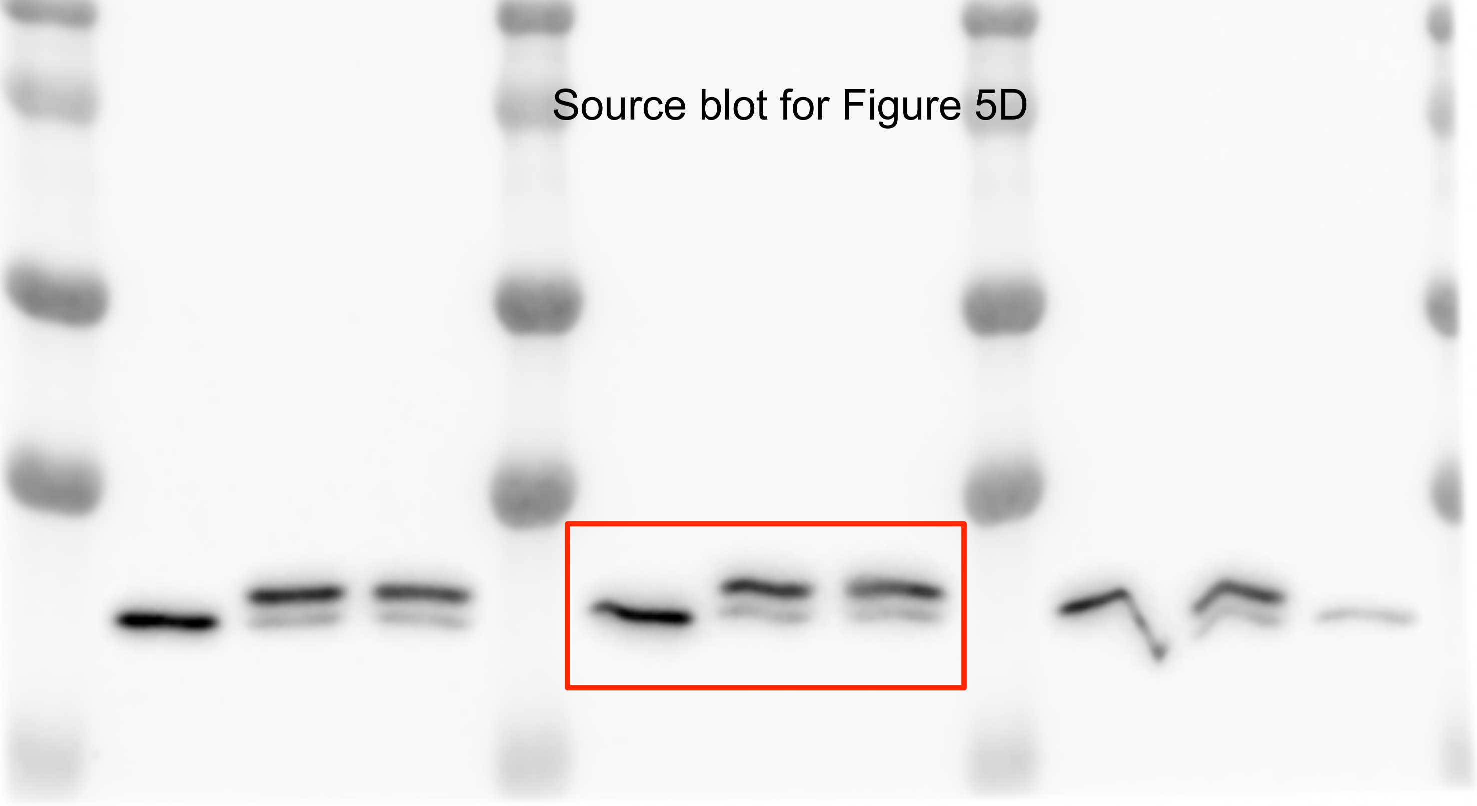

Red marked area was used for the figure.

Membrane was cut after transfer, and before probing by Ab
other bands are same proteins under different conditions

Fig. 5. PKCα and PKCβ bind to CycT1 for its phosphorylation, also promote interactions between CycT1 and CDK9, and increase the stability of CycT1

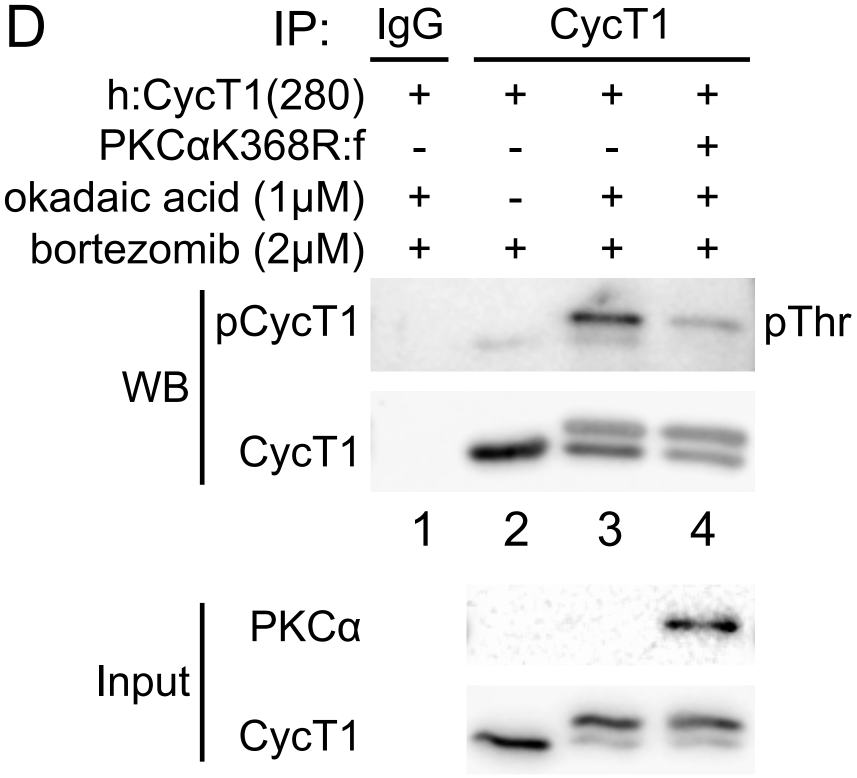

Source blot for Figure 5E

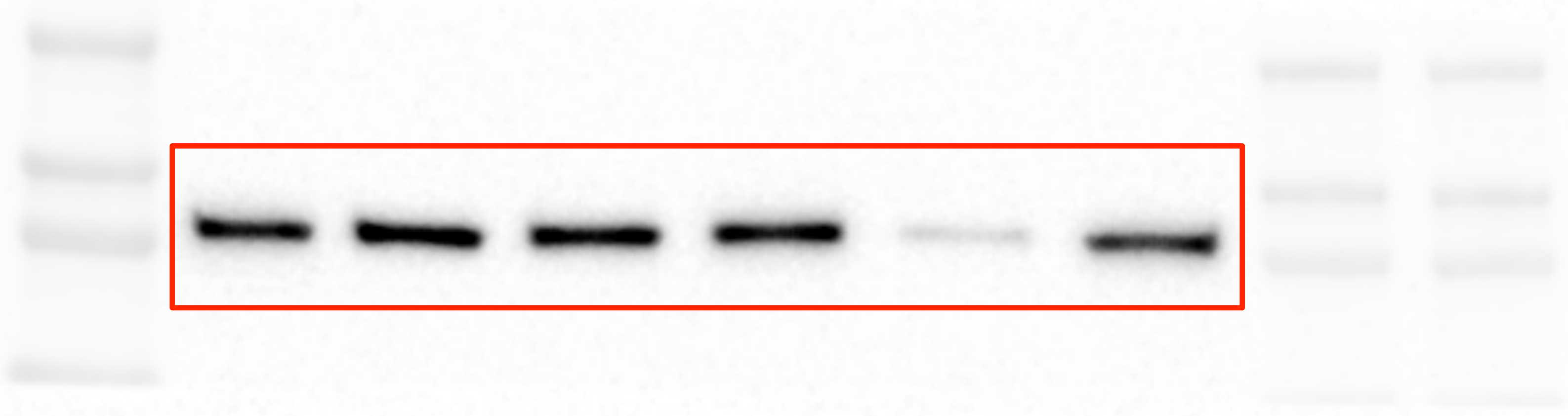

Red marked area was used for the figure.

Membrane was cut after transfer, and before probing by Ab

Source blot for Figure 5E

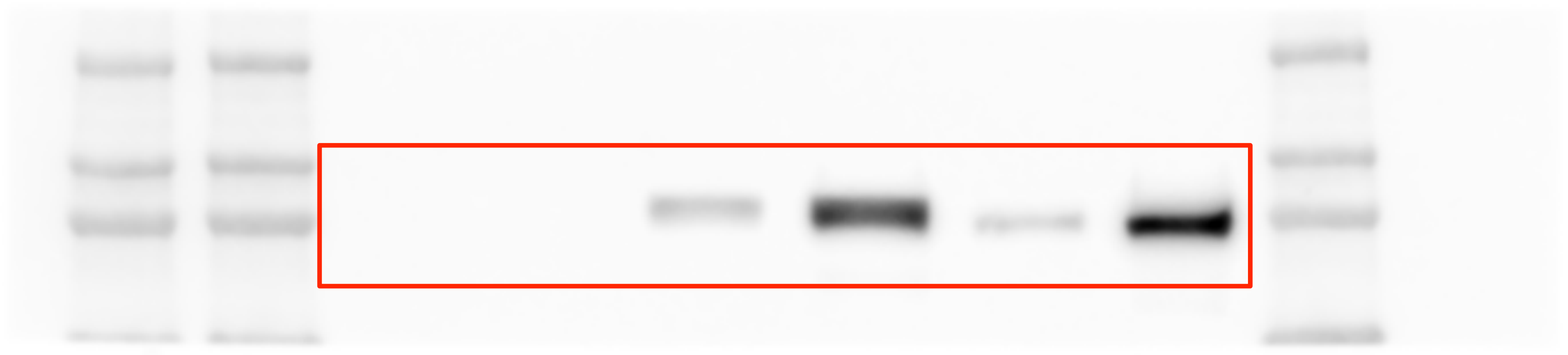

Red marked area was used for the figure.

Membrane was cut after transfer, and before probing by Ab

Source blot for Figure 5E

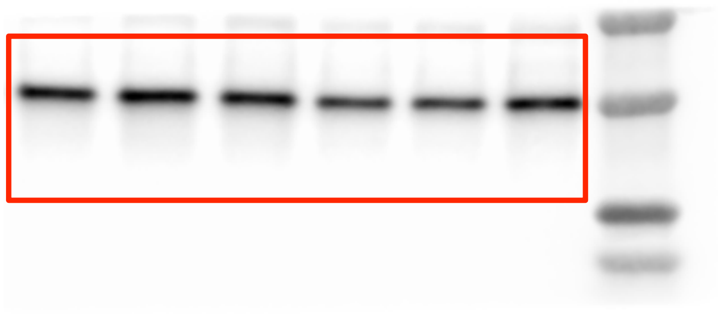

Red marked area was used for the figure.

Membrane was cut after transfer, and before probing by Ab

Source blot for Figure 5E

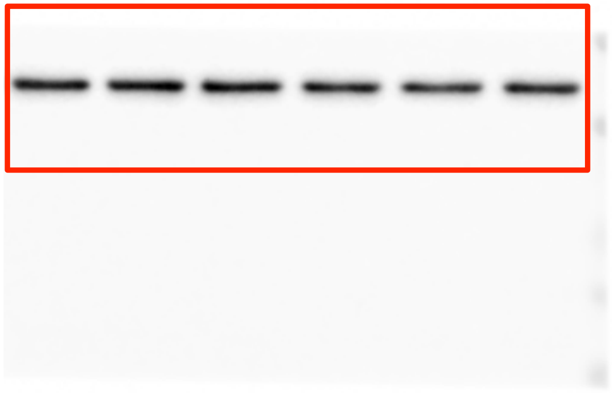

Red marked area was used for the figure.
Membrane was cut after transfer, and before probing by Ab

Fig. 5. PKCα and PKCβ bind to CycT1 for its phosphorylation, also promote interactions between CycT1 and CDK9, and increase the stability of CycT1

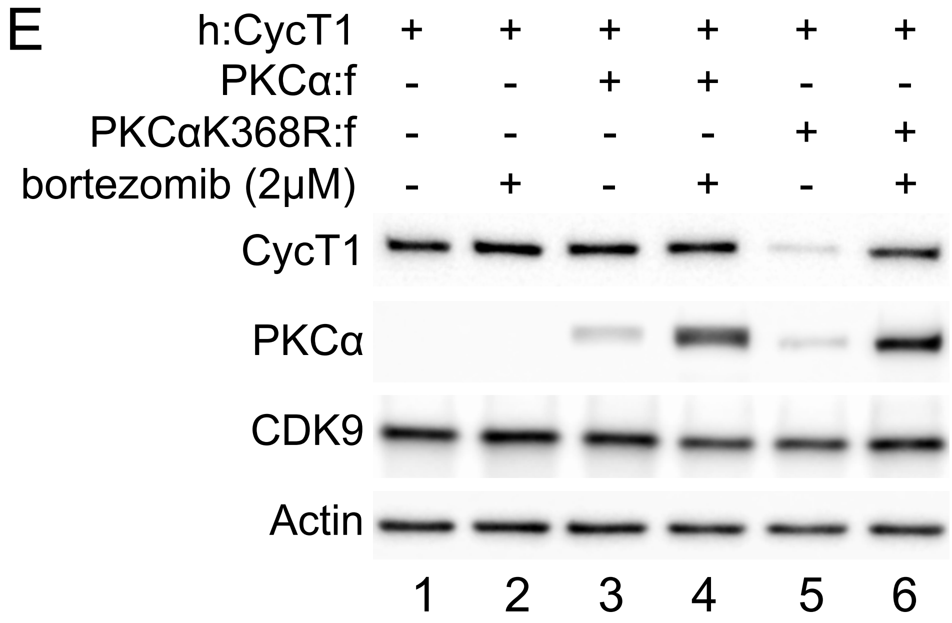

Source blot for Figure 5F

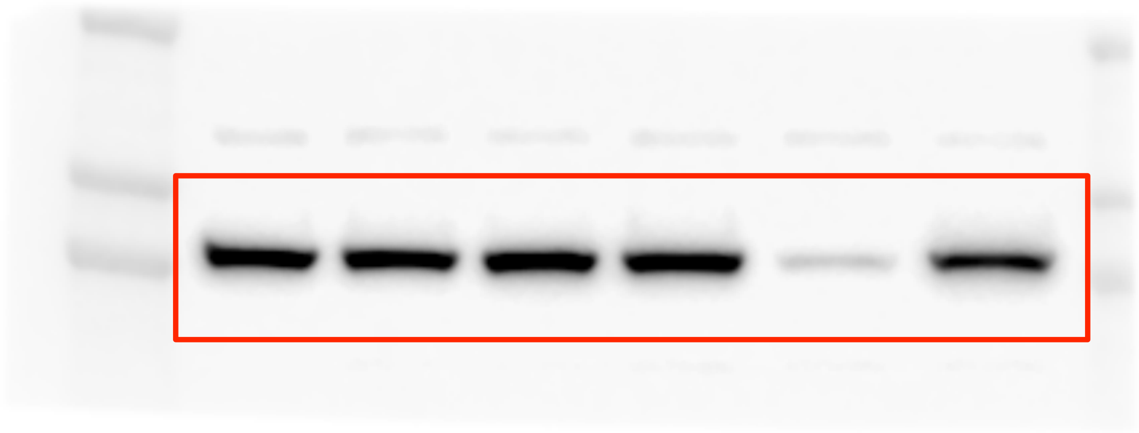

Red marked area was used for the figure.

Membrane was cut after transfer, and before probing by Ab

Source blot for Figure 5F

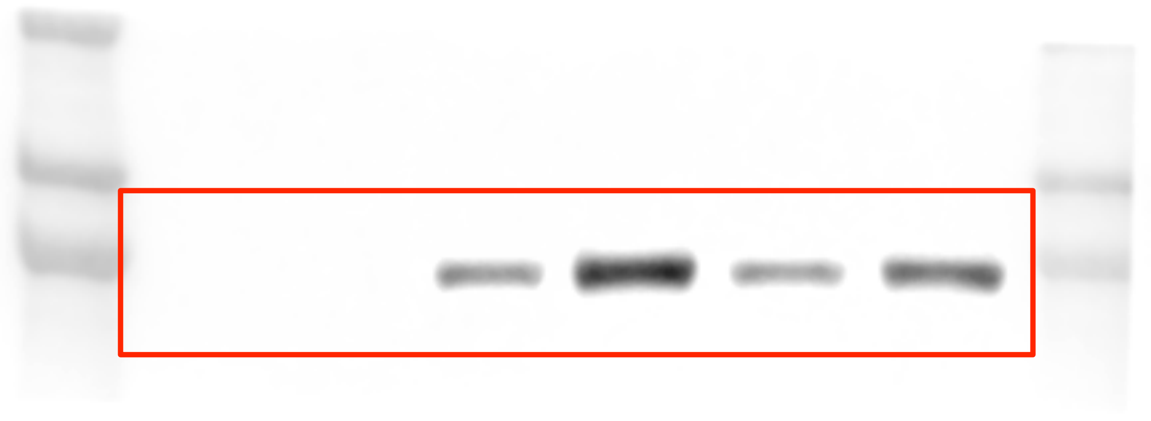

Red marked area was used for the figure.

Membrane was cut after transfer, and before probing by Ab

Source blot for Figure 5F

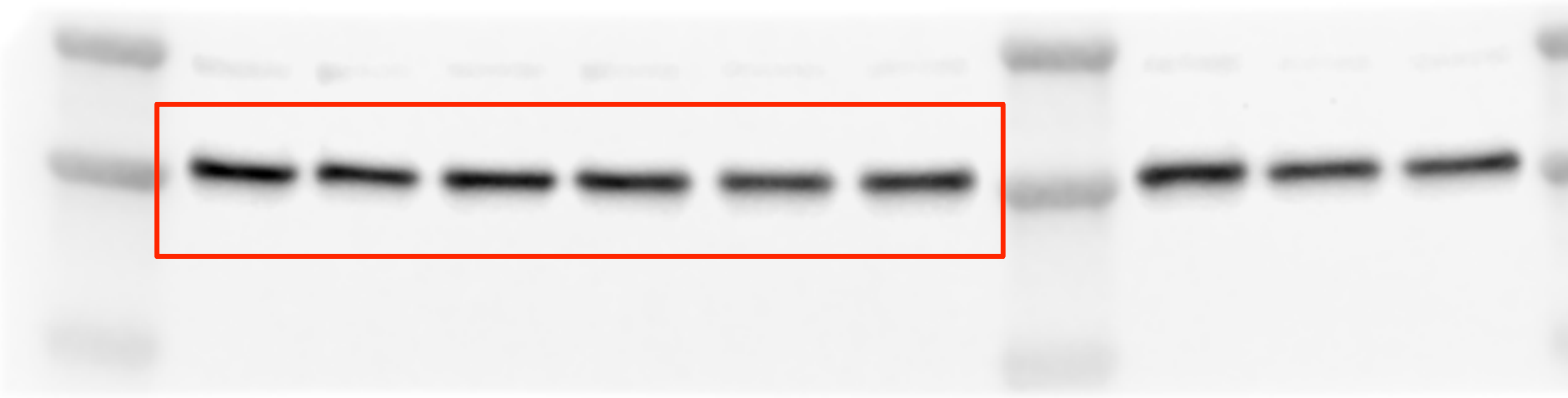

Red marked area was used for the figure.

Membrane was cut after transfer, and before probing by Ab

Right lanes are the same proteins under different conditions

Source blot for Figure 5F

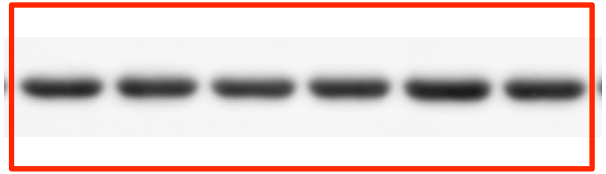

Red marked area was used for the figure.

Membrane was cut after transfer, and before probing by Ab

Fig. 5. PKCα and PKCβ bind to CycT1 for its phosphorylation, also promote interactions between CycT1 and CDK9, and increase the stability of CycT1

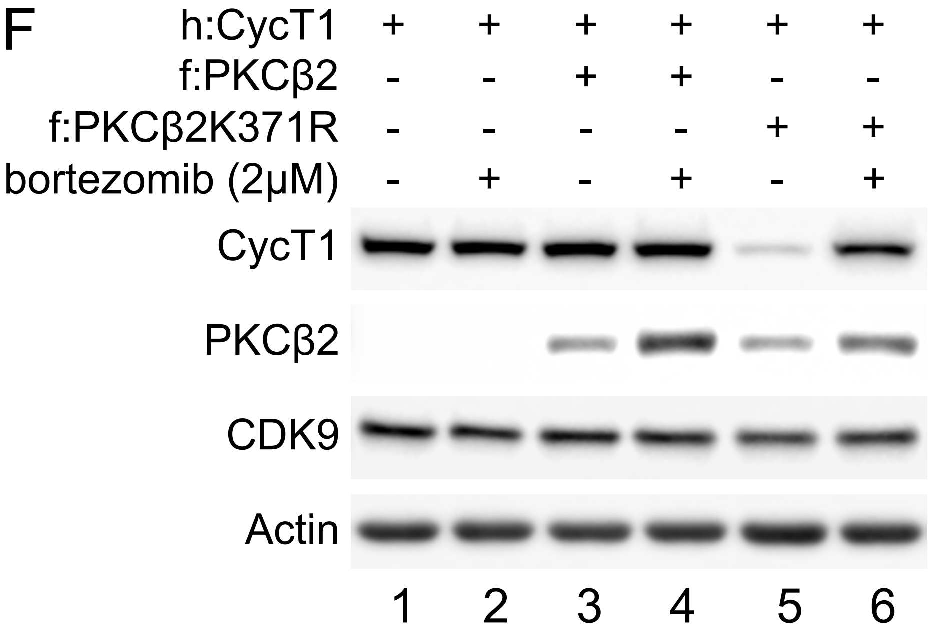

Source blot for Figure 5G

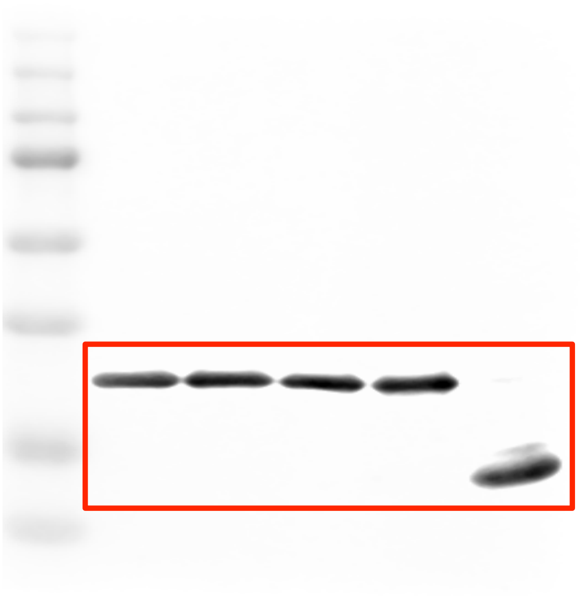

Red marked area was used for the figure.

Source gel-staining for Figure 5G

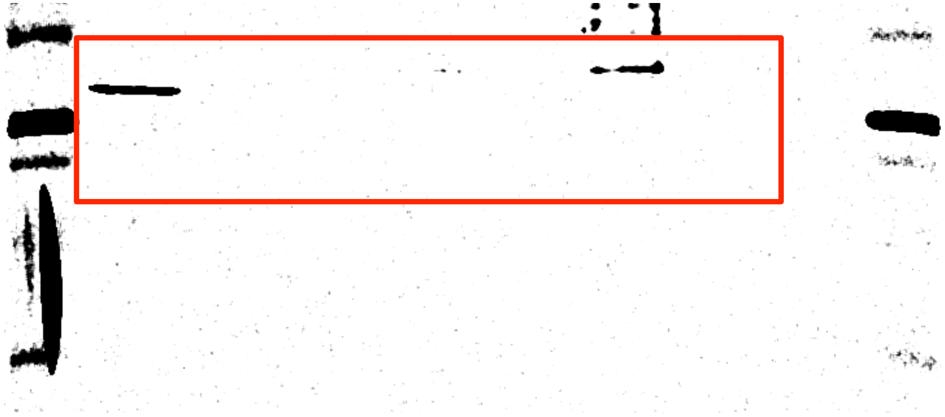

Red marked area was used for the figure.

Fig. 5. PKCα and PKCβ bind to CycT1 for its phosphorylation, also promote interactions between CycT1 and CDK9, and increase the stability of CycT1

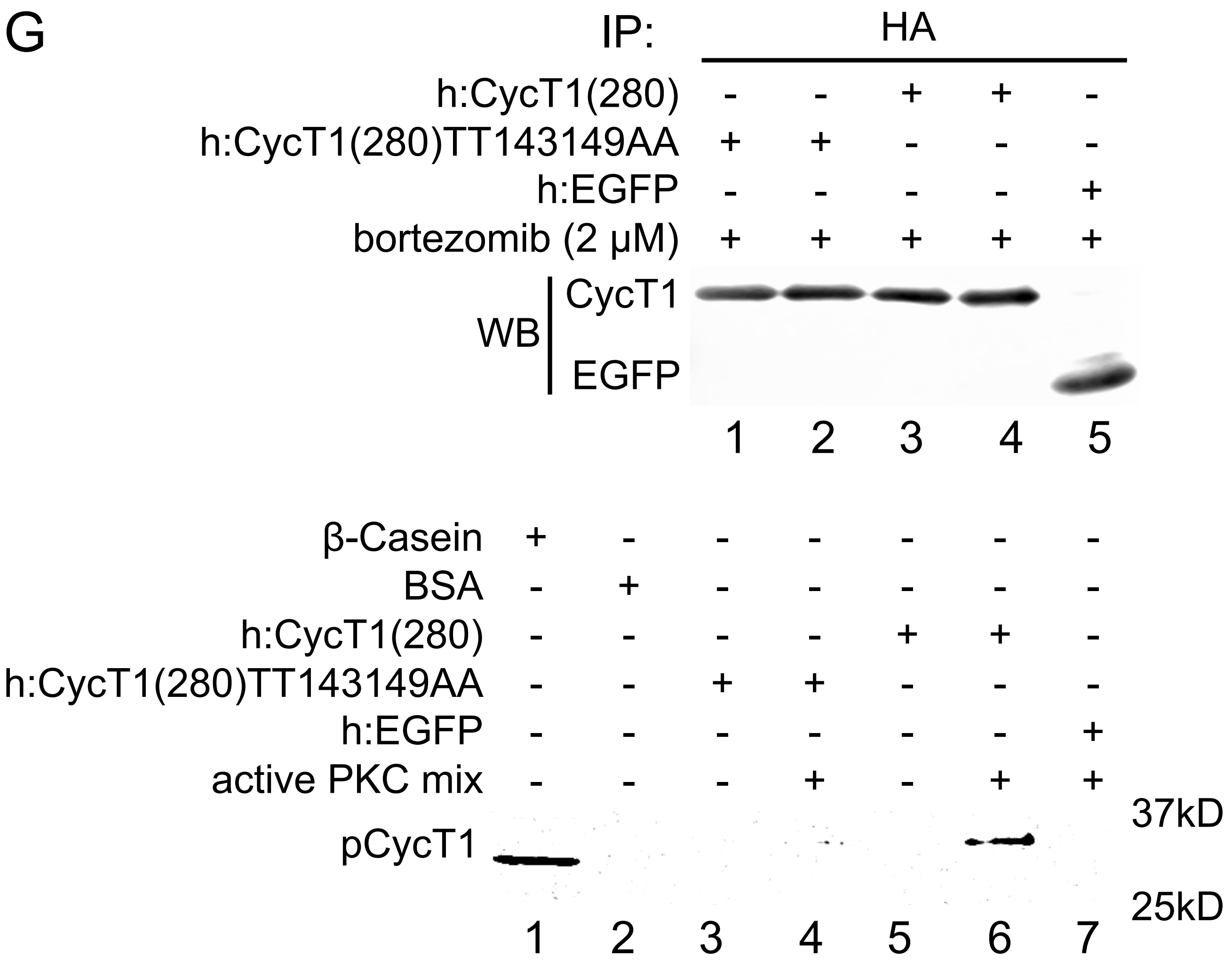

Source blot for Figure 6A

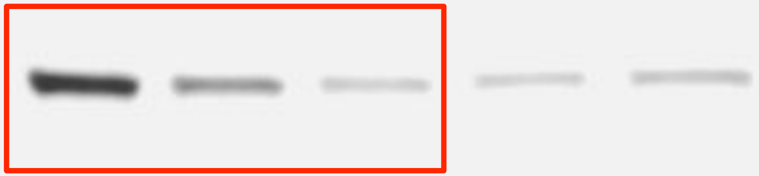

Red marked area was used for the figure.

Membrane was cut after transfer, and before probing by Ab
Right 2 lanes the same proteins under PMA treatment for 120 h and 144 h

Source blot for Figure 6A

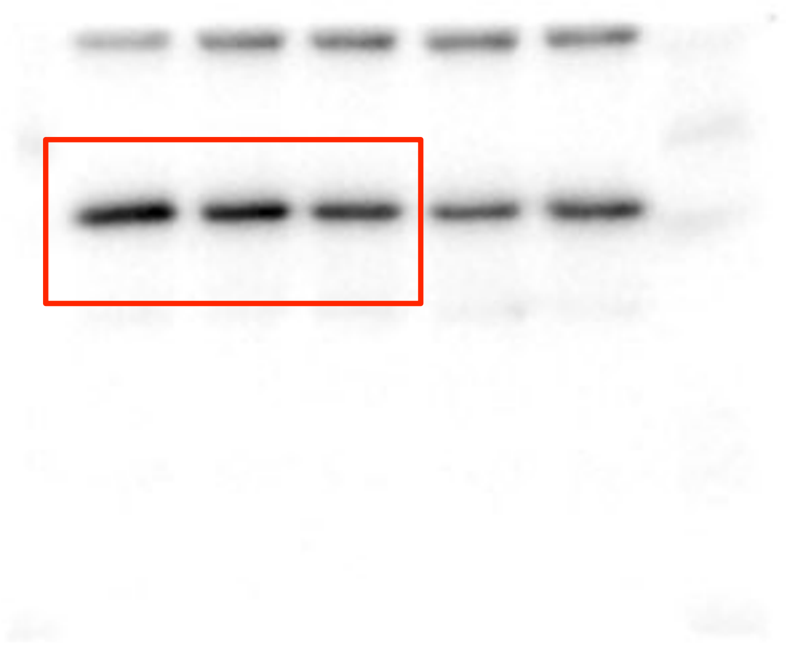

Red marked area was used for the figure.

Membrane was cut after transfer, and before probing by Ab
Right 2 lanes the same proteins under PMA treatment for 120 h and 144 h

Source blot for Figure 6A

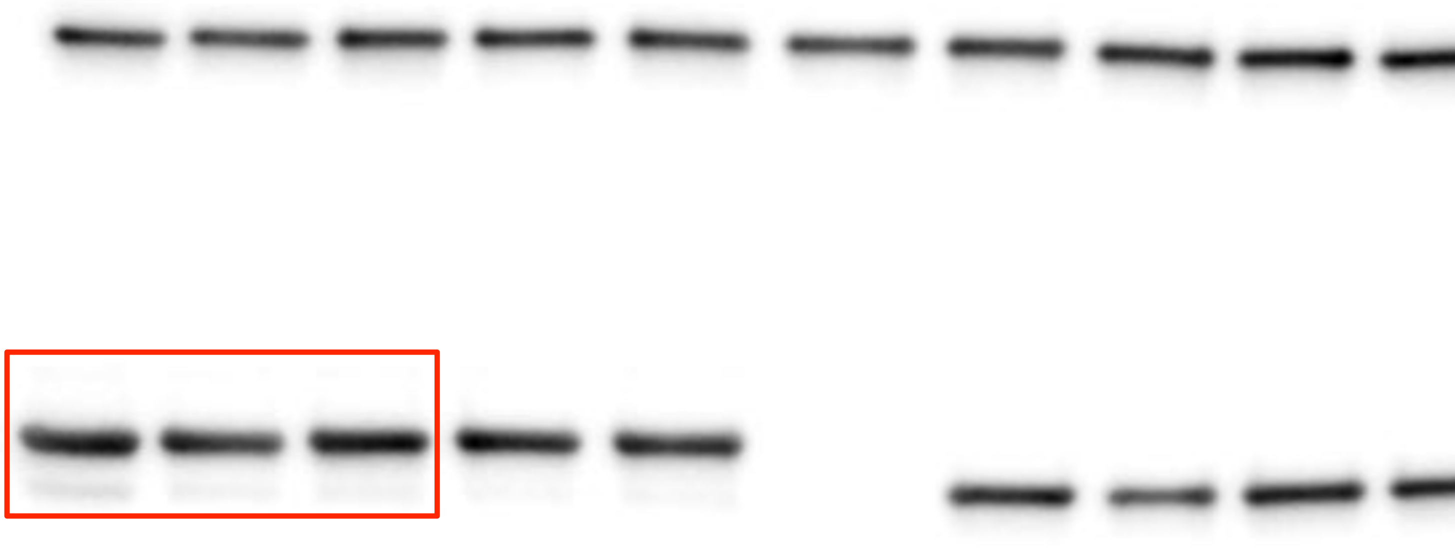

Red marked area was used for the figure.

Membrane was cut after transfer, and before probing by Ab
Right 2 lanes the same proteins under PMA treatment for 120 h and 144 h
3 different membranes are in the same tray for imaging

Fig. 6. Depletion of PKCs leads to decreased levels of CycT1 in cell lines and primary cells

Jurkat Cells

A

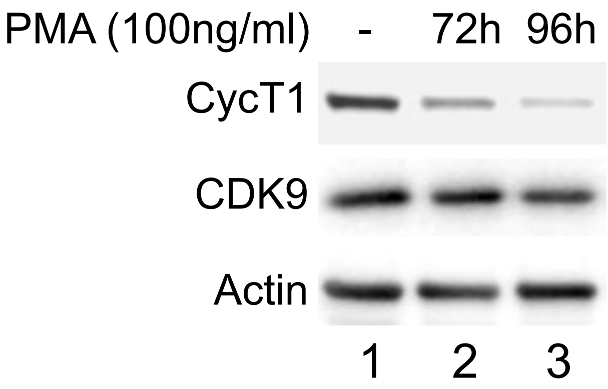

Source blot for Figure 6B

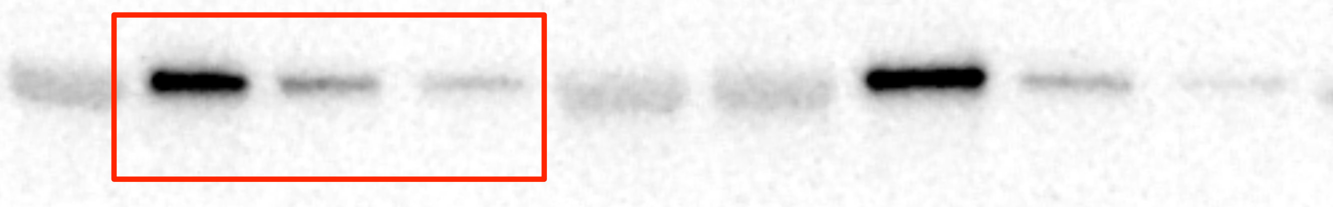

Red marked area was used for the figure.

Membrane was cut after transfer, and before probing by Ab
Right lanes the same proteins from 2nd donor

Source blot for Figure 6B

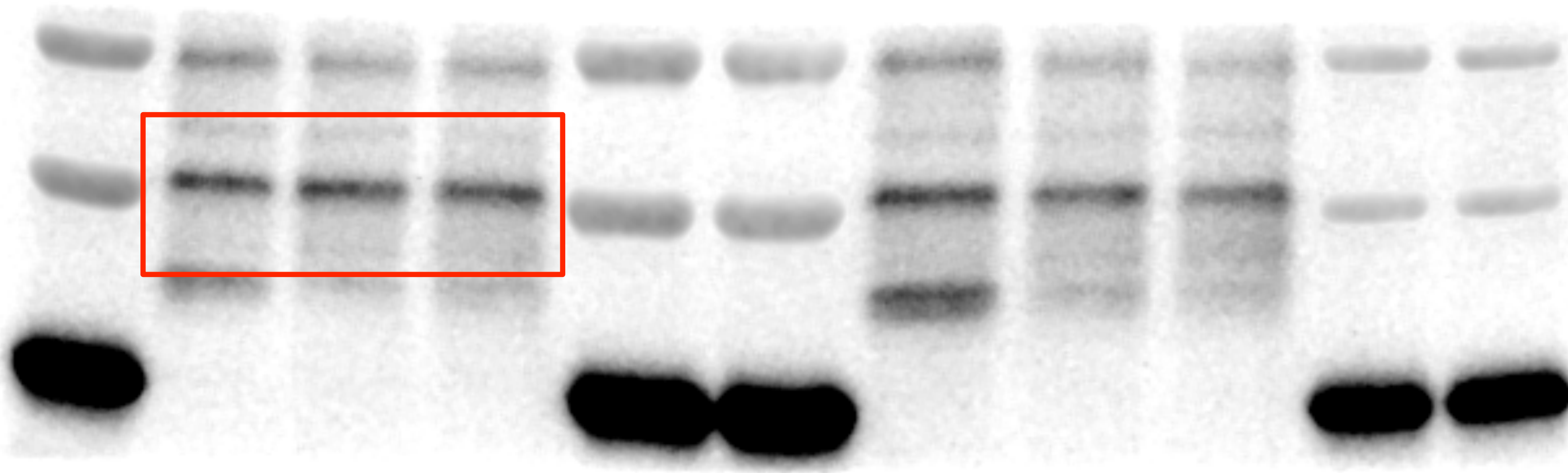

Red marked area was used for the figure.

Membrane was cut after transfer, and before probing by Ab
Right lanes the same proteins from 2nd donor

Source blot for Figure 6B

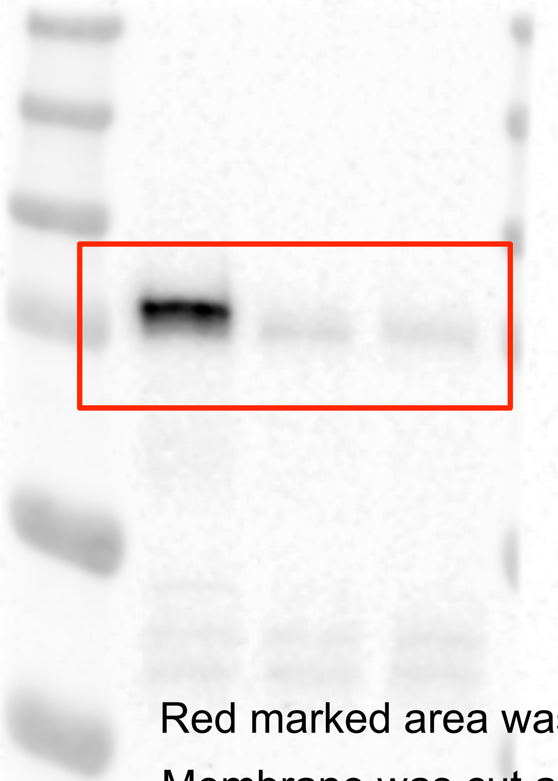

Red marked area was used for the figure.

Membrane was cut after transfer, and before probing by Ab

Source blot for Figure 6B

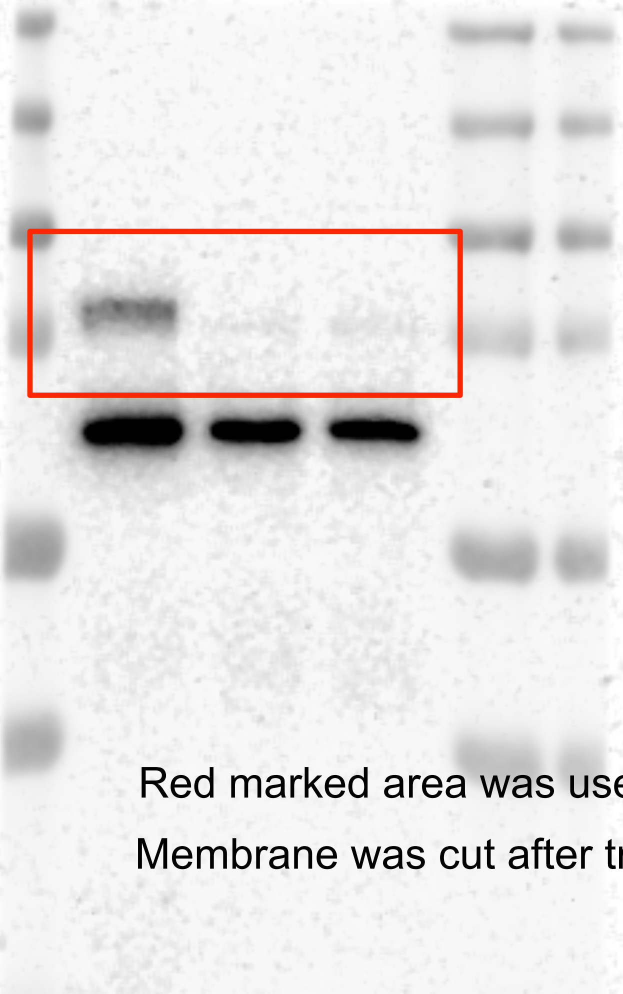

Red marked area was used for the figure.

Membrane was cut after transfer, and before probing by Ab

Source blot for Figure 6B

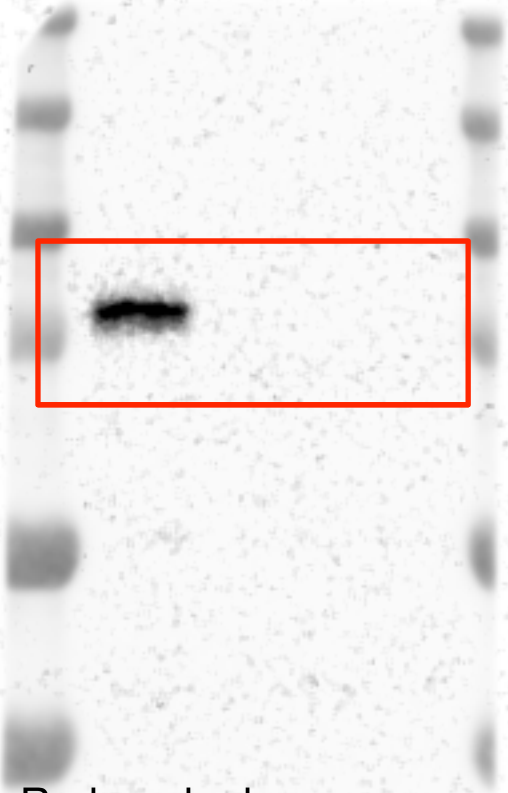

Red marked area was used for the figure.

Membrane was cut after transfer, and before probing by Ab

Source blot for Figure 6B

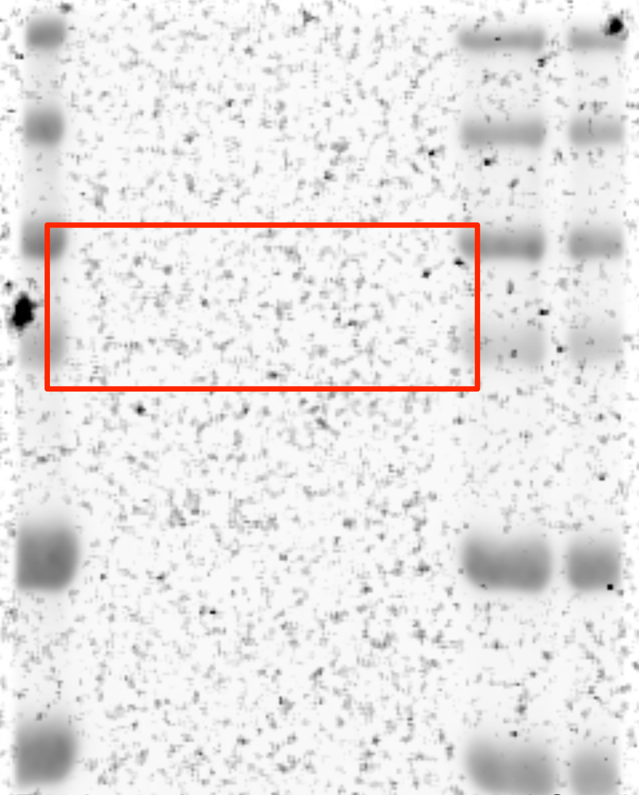

Red marked area was used for the figure.
Membrane was cut after transfer, and before probing by Ab

Source blot for Figure 6B

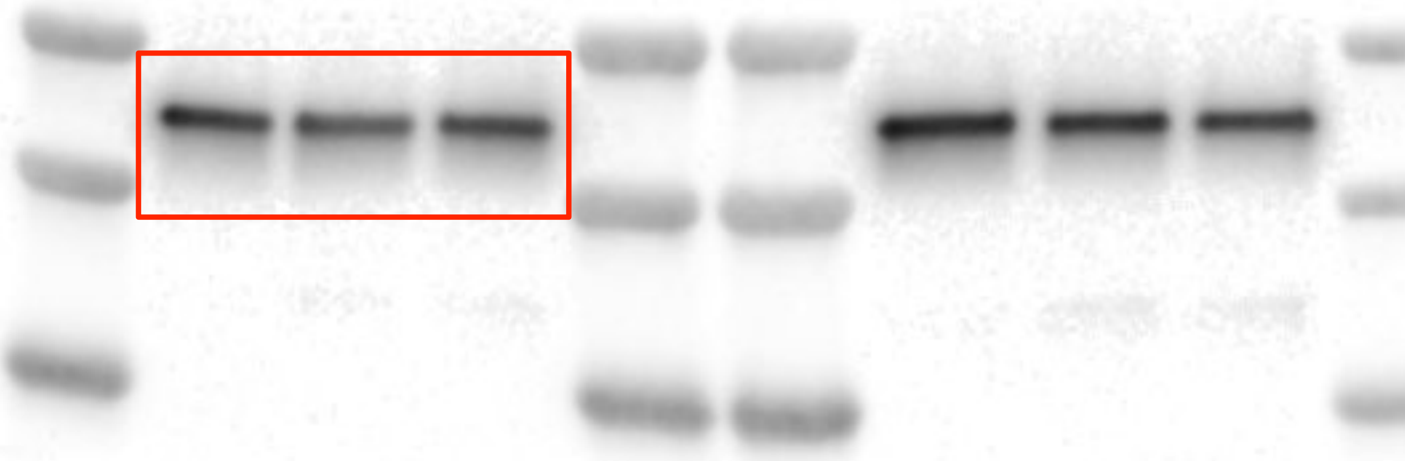

Red marked area was used for the figure.

Membrane was cut after transfer, and before probing by Ab
Right lanes the same proteins from 2nd donor

Fig. 6. Depletion of PKCs leads to decreased levels of CycT1 in cell lines and primary cells

Activated Primary CD4+ T cells

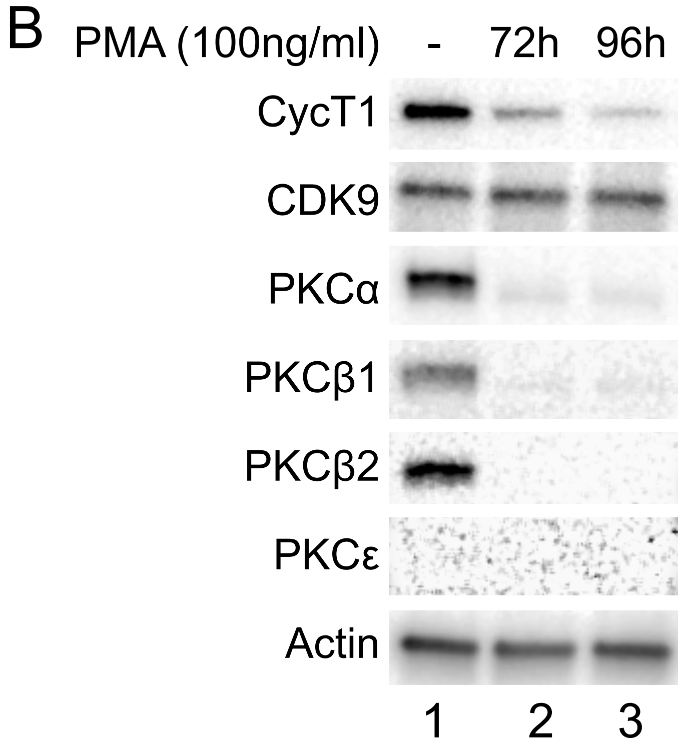

Source blot for Figure 6C

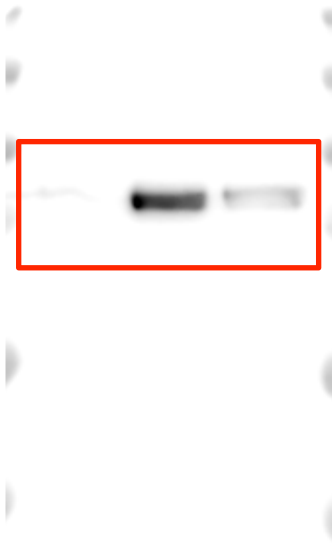

Red marked area was used for the figure.

Membrane was cut after transfer, and before probing by Ab

Source blot for Figure 6C

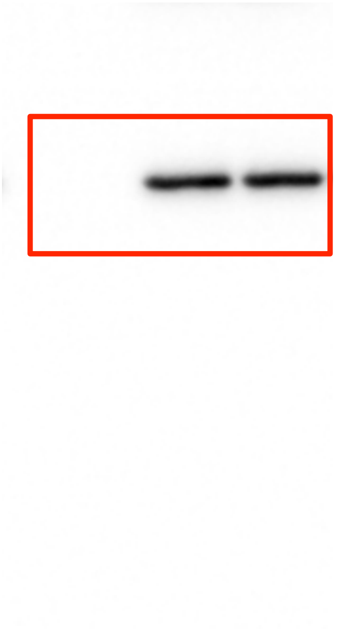

Red marked area was used for the figure.

Membrane was cut after transfer, and before probing by Ab

Source blot for Figure 6C

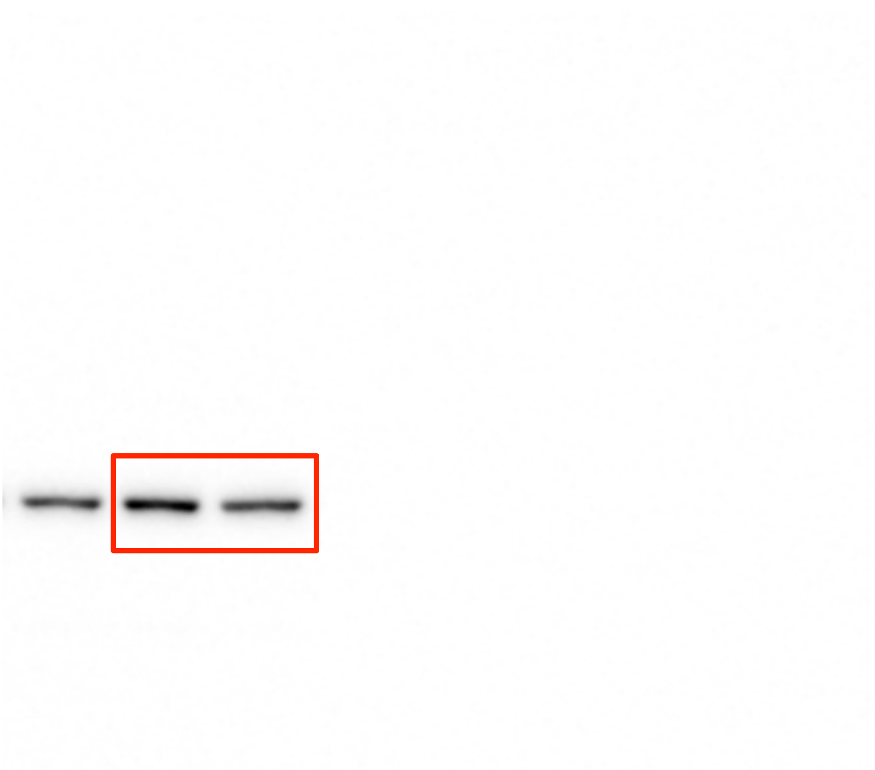

Red marked area was used for the figure.

Membrane was cut after transfer, and before probing by Ab
Left lane is the same protein without treatment

Source blot for Figure 6C

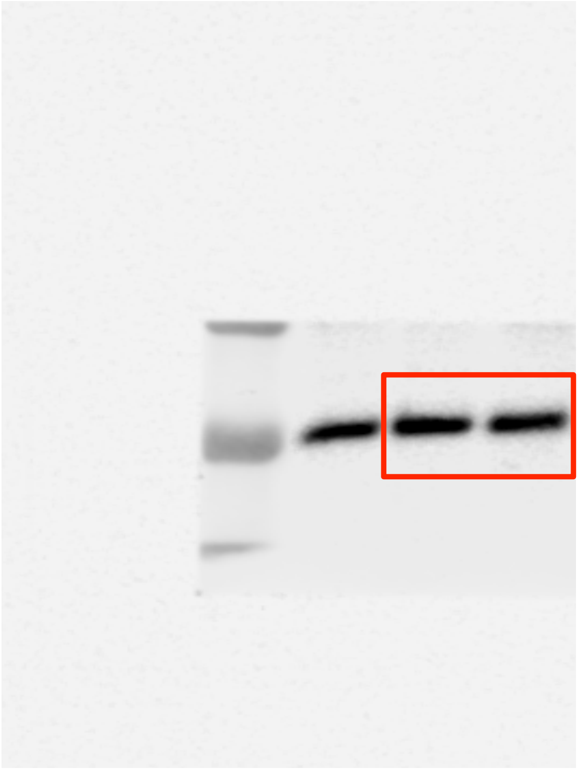

Red marked area was used for the figure.

Membrane was cut after transfer, and before probing by Ab
Left lane is the same protein without treatment

Fig. 6. Depletion of PKCs leads to decreased levels of CycT1 in cell lines and primary cells

Activated Primary CD4+ T cells

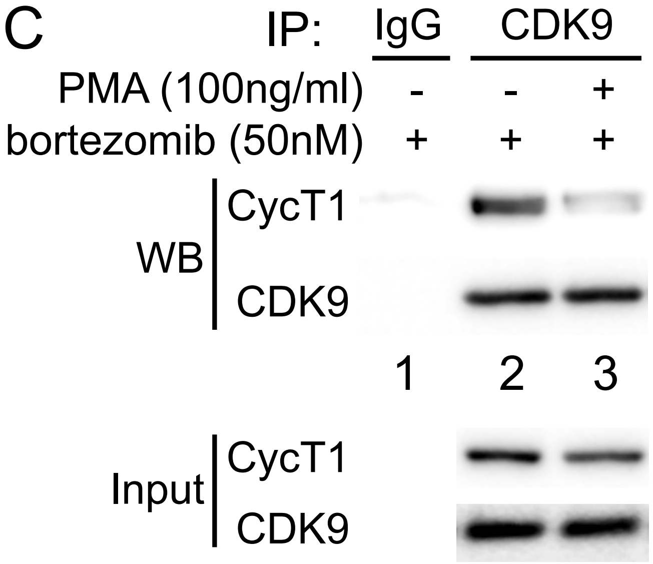

Source blot for Figure 6D

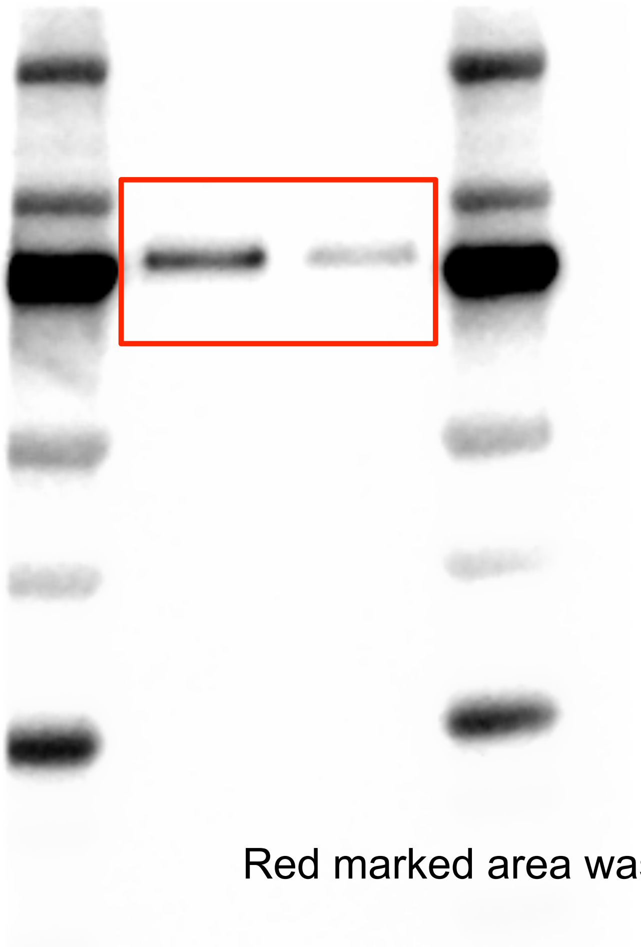

Red marked area was used for the figure.

Source blot for Figure 6D

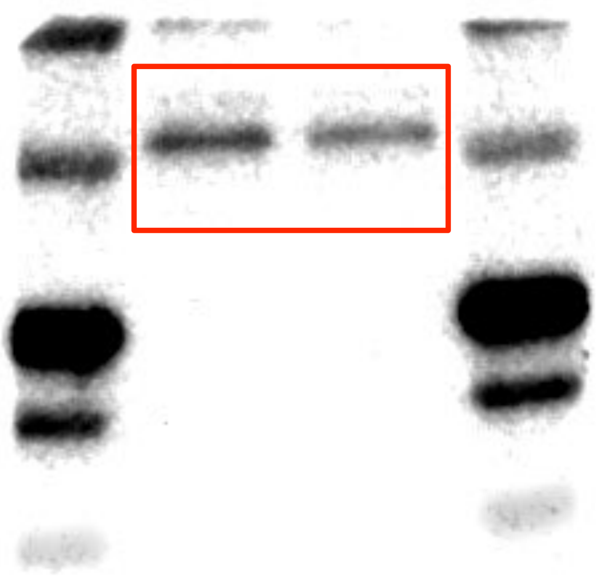

Red marked area was used for the figure.

Membrane was cut after transfer, and before probing by Ab

Source blot for Figure 6D

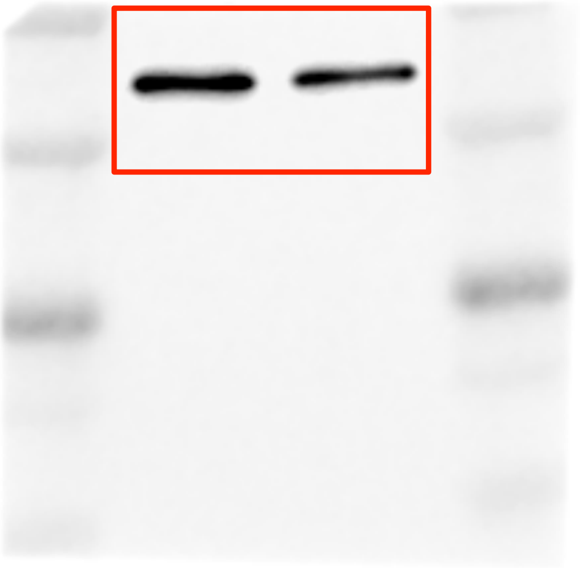

Red marked area was used for the figure.

Membrane was cut after transfer, and before probing by Ab

Fig. 6. Depletion of PKCs leads to decreased levels of CycT1 in cell lines and primary cells

Mouse anergic T cells

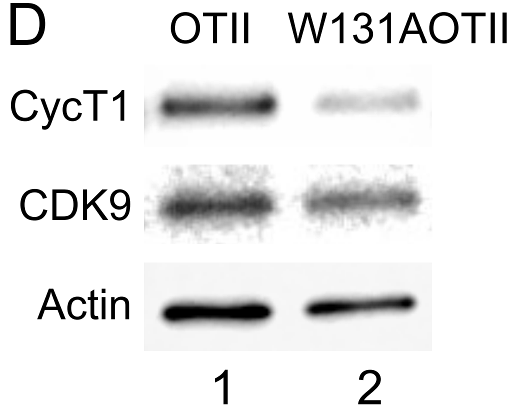

Source data for Figure 6E

Ensembl_ID	Gene	Description	Peripheral OT2 T cells			Peripheral W131AOT2		
ENSMUSG00000011960	Ccnt1	cyclin T1 [Source:MGI Symbol;Acc:MGI:1328363]	3808	4229.6	4076.9	3608.1	3656.1	4889.9
ENSMUSG00000009555	Cdk9	cyclin-dependent kinase 9 (CDC2-related kinase) [Sou	4060.6	4500	4485.6	4459.2	5112.8	5461.8

CycT1/OTII	3808	4038.167	0.943002	1	
	4229.6	4038.167	1.047406		
	4076.9	4038.167	1.009592		
CycT1/W131AOTII	3608.1	4038.167	0.8935	1.003269	0.17993
	3656.1	4038.167	0.905386		
	4889.9	4038.167	1.210921		
CDK9/OTII	4060.6	4348.733	0.933743	1	
	4500	4348.733	1.034784		
	4485.6	4348.733	1.031473		
CDK9/W131AOTII	4459.2	4348.733	1.025402	1.152351	0.117035
	5112.8	4348.733	1.175699		
	5461.8	4348.733	1.255952		

CycT1/OTII	1	0
CycT1/W131AOTII	1.003269	0.17993
CDK9/OTII	1	0
CDK9/W131AOTII	1.152351	0.117035

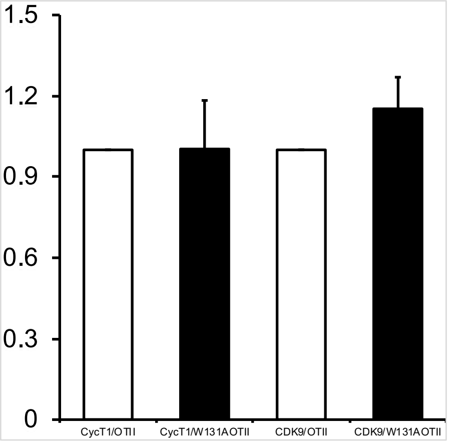

Fig. 6. Depletion of PKCs leads to decreased levels of CycT1 in cell lines and primary cells

Mouse anergic T cells

E

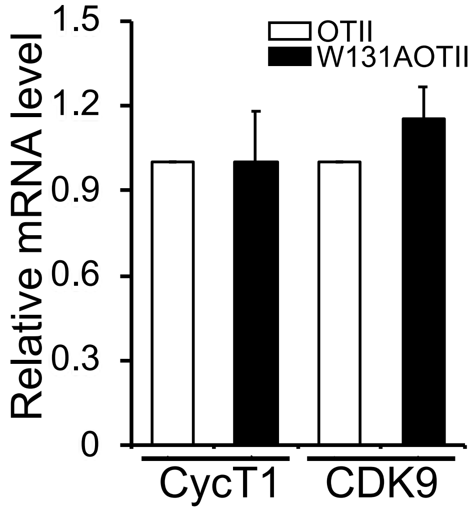

Source blot for Figure 6F

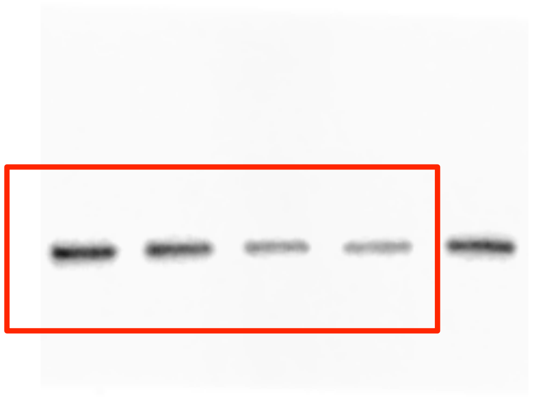

Red marked area was used for the figure.

Membrane was cut after transfer, and before probing by Ab
Right lane is the same protein without treatment, same as lane 1

Source blot for Figure 6F

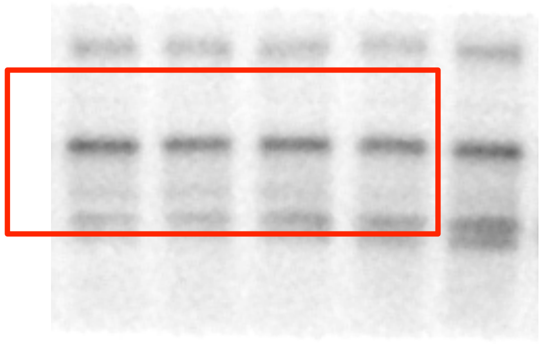

Red marked area was used for the figure.

Membrane was cut after transfer, and before probing by Ab
Right lane is the same protein without treatment, same as lane 1

Source blot for Figure 6F

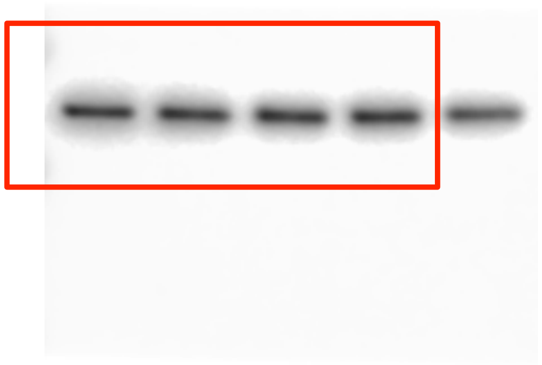

Red marked area was used for the figure.

Membrane was cut after transfer, and before probing by Ab
Right lane is the same protein without treatment, same as lane 1

Fig. 6. Depletion of PKCs leads to decreased levels of CycT1 in cell lines and primary cells

Activated Primary CD4+ T cells

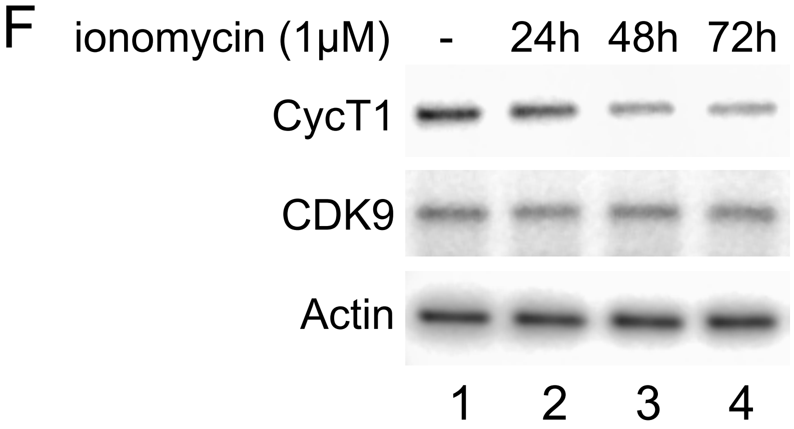

Figure supplement 1. Thr143 and T149 are the phosphorylated residues in CycT1

A

```
    EAYLQQVQDLVILESIILQTLGFELTIDHPHTHVVKCTQLVRA          #     50
%1  ...................T.....T.................
```

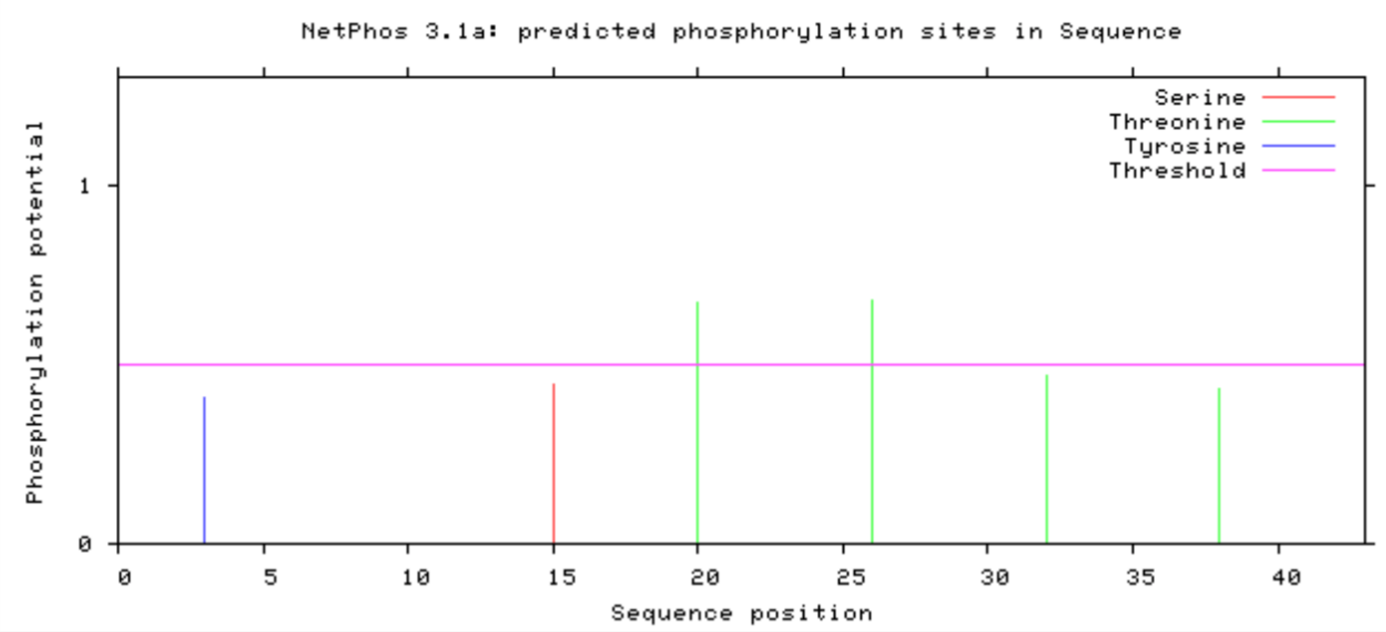

NetPhos 3.1 Server - prediction results

Technical University of Denmark

```
>Sequence       43 amino acids
#
# netphos-3.1b prediction results
#
# Sequence                 # x   Context     Score   Kinase    Answer
# -------------------------------------------------------------------
# Sequence                 3 Y   --EAYLQQV   0.409   EGFR        .
# Sequence                 3 Y   --EAYLQQV   0.360   INSR        .
# Sequence                 3 Y   --EAYLQQV   0.343   unsp        .
# Sequence                 3 Y   --EAYLQQV   0.313   SRC         .
#
# Sequence                15 S   VILESIILQ   0.445   CaM-II      .
# Sequence                15 S   VILESIILQ   0.443   PKC         .
# Sequence                15 S   VILESIILQ   0.429   GSK3        .
# Sequence                15 S   VILESIILQ   0.414   CKII        .
# Sequence                15 S   VILESIILQ   0.413   PKA         .
# Sequence                15 S   VILESIILQ   0.387   CKI         .
# Sequence                15 S   VILESIILQ   0.363   cdc2        .
# Sequence                15 S   VILESIILQ   0.345   DNAPK       .
# Sequence                15 S   VILESIILQ   0.331   p38MAPK     .
# Sequence                15 S   VILESIILQ   0.307   RSK         .
# Sequence                15 S   VILESIILQ   0.266   ATM         .
# Sequence                15 S   VILESIILQ   0.252   PKG         .
# Sequence                15 S   VILESIILQ   0.124   cdk5        .
# Sequence                15 S   VILESIILQ   0.076   PKB         .
# Sequence                15 S   VILESIILQ   0.008   unsp        .
#
# Sequence                20 T   IILQTLGFE   0.672   PKC        YES
# Sequence                20 T   IILQTLGFE   0.463   cdc2        .
# Sequence                20 T   IILQTLGFE   0.437   PKA         .
# Sequence                20 T   IILQTLGFE   0.436   GSK3        .
# Sequence                20 T   IILQTLGFE   0.423   CaM-II      .
# Sequence                20 T   IILQTLGFE   0.374   CKI         .
# Sequence                20 T   IILQTLGFE   0.371   CKII        .
# Sequence                20 T   IILQTLGFE   0.350   DNAPK       .
# Sequence                20 T   IILQTLGFE   0.313   p38MAPK     .
# Sequence                20 T   IILQTLGFE   0.236   ATM         .
# Sequence                20 T   IILQTLGFE   0.228   PKG         .
# Sequence                20 T   IILQTLGFE   0.220   RSK         .
# Sequence                20 T   IILQTLGFE   0.161   cdk5        .
# Sequence                20 T   IILQTLGFE   0.089   PKB         .
# Sequence                20 T   IILQTLGFE   0.049   unsp        .
#
# Sequence                26 T   GFELTIDHP   0.680   unsp       YES
# Sequence                26 T   GFELTIDHP   0.553   CKI        YES
# Sequence                26 T   GFELTIDHP   0.457   CaM-II      .
# Sequence                26 T   GFELTIDHP   0.434   GSK3        .
# Sequence                26 T   GFELTIDHP   0.420   cdc2        .
# Sequence                26 T   GFELTIDHP   0.400   CKII        .
# Sequence                26 T   GFELTIDHP   0.349   DNAPK       .
# Sequence                26 T   GFELTIDHP   0.338   p38MAPK     .
# Sequence                26 T   GFELTIDHP   0.258   ATM         .
# Sequence                26 T   GFELTIDHP   0.246   PKG         .
# Sequence                26 T   GFELTIDHP   0.226   RSK         .
# Sequence                26 T   GFELTIDHP   0.182   cdk5        .
# Sequence                26 T   GFELTIDHP   0.150   PKC         .
# Sequence                26 T   GFELTIDHP   0.130   PKA         .
# Sequence                26 T   GFELTIDHP   0.085   PKB         .
#
# Sequence                32 T   DHPHTHVVK   0.468   GSK3        .
# Sequence                32 T   DHPHTHVVK   0.405   cdc2        .
# Sequence                32 T   DHPHTHVVK   0.404   CaM-II      .
# Sequence                32 T   DHPHTHVVK   0.400   p38MAPK     .
# Sequence                32 T   DHPHTHVVK   0.382   CKI         .
# Sequence                32 T   DHPHTHVVK   0.373   CKII        .
# Sequence                32 T   DHPHTHVVK   0.343   DNAPK       .
# Sequence                32 T   DHPHTHVVK   0.282   unsp        .
# Sequence                32 T   DHPHTHVVK   0.243   ATM         .
# Sequence                32 T   DHPHTHVVK   0.242   PKG         .
# Sequence                32 T   DHPHTHVVK   0.231   RSK         .
# Sequence                32 T   DHPHTHVVK   0.200   cdk5        .
# Sequence                32 T   DHPHTHVVK   0.195   PKC         .
# Sequence                32 T   DHPHTHVVK   0.108   PKA         .
# Sequence                32 T   DHPHTHVVK   0.080   PKB         .
#
# Sequence                38 T   VVKCTQLVR   0.431   ATM         .
# Sequence                38 T   VVKCTQLVR   0.423   GSK3        .
# Sequence                38 T   VVKCTQLVR   0.423   PKG         .
# Sequence                38 T   VVKCTQLVR   0.413   CaM-II      .
# Sequence                38 T   VVKCTQLVR   0.402   p38MAPK     .
# Sequence                38 T   VVKCTQLVR   0.376   cdc2        .
# Sequence                38 T   VVKCTQLVR   0.373   DNAPK       .
# Sequence                38 T   VVKCTQLVR   0.361   CKI         .
# Sequence                38 T   VVKCTQLVR   0.324   CKII        .
# Sequence                38 T   VVKCTQLVR   0.263   PKC         .
# Sequence                38 T   VVKCTQLVR   0.231   RSK         .
# Sequence                38 T   VVKCTQLVR   0.216   PKA         .
# Sequence                38 T   VVKCTQLVR   0.175   cdk5        .
# Sequence                38 T   VVKCTQLVR   0.079   PKB         .
# Sequence                38 T   VVKCTQLVR   0.026   unsp        .
#
    EAYLQQVQDLVILESIILQTLGFELTIDHPHTHVVKCTQLVRA          #     50
%1  ...................T.....T.................
```

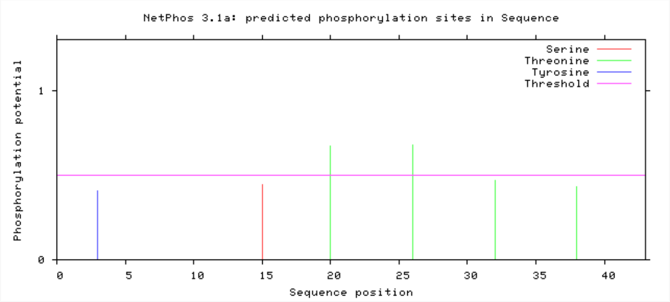

Explain the output. Go **back**.

Source blot for Figure supplement 1B

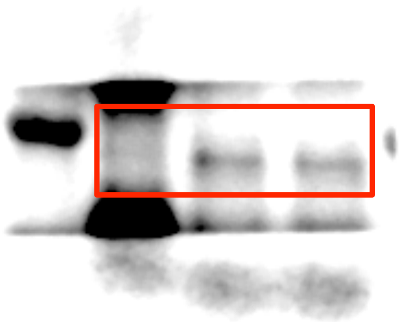

Red marked area was used for the figure.

Membrane was cut after transfer, and before probing by Ab

Source blot for Figure supplement 1B

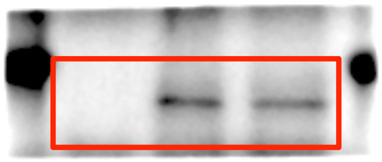

Red marked area was used for the figure.

Membrane was cut after transfer, and before probing by Ab

Fig. S1.

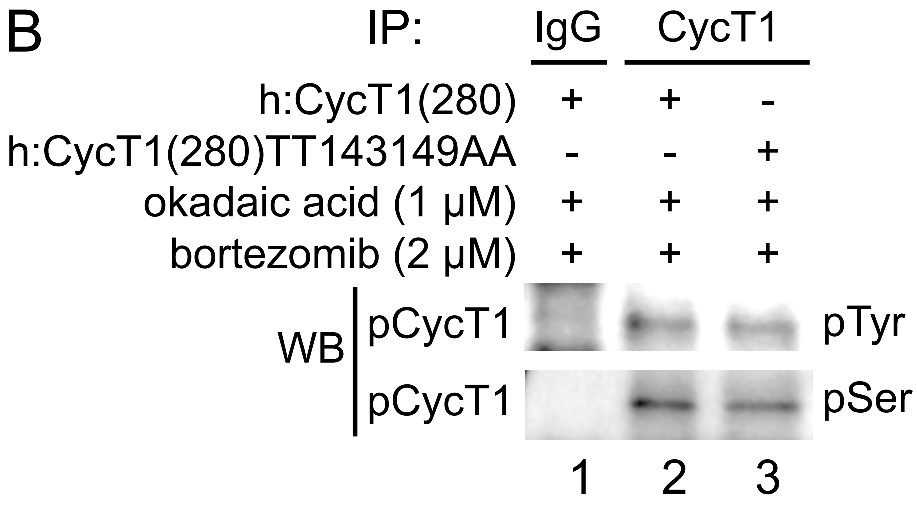
B
IP:
IgG
CycT1
h:CycT1(280) + + -
h:CycT1(280)TT143149AA - - +
okadaic acid (1 µM) + + +
bortezomib (2 µM) + + +
WB
pCycT1
pCycT1
pTyr
pSer
1 2 3

Source blot for Figure supplement 1C

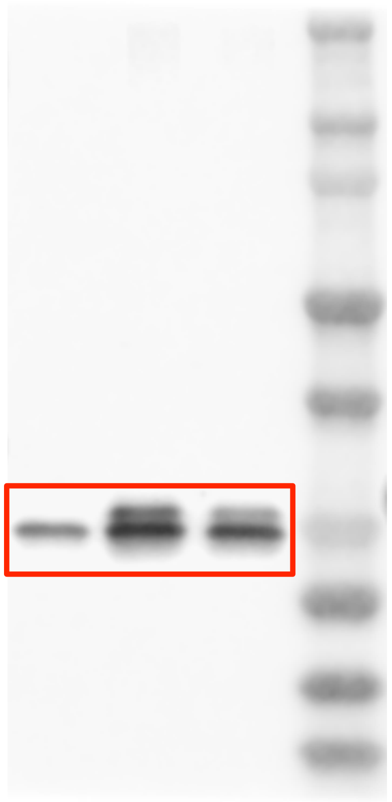

Red marked area was used for the figure.

Membrane was cut after transfer, and before probing by Ab

Fig. S1.

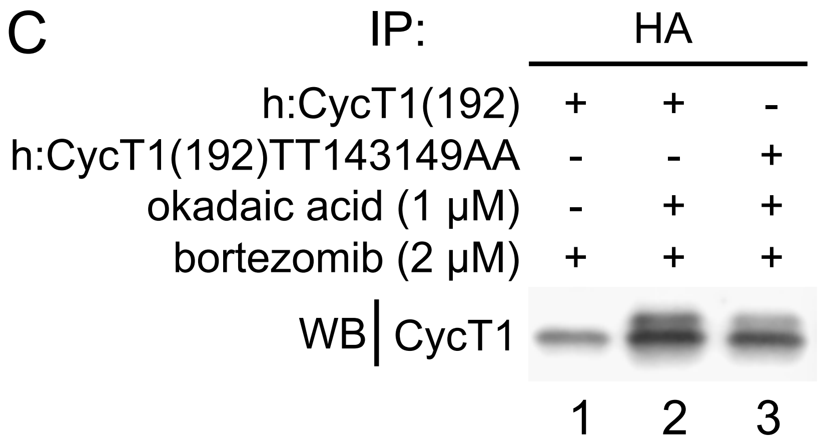
C
IP:
HA
h:CycT1(192) + + -
h:CycT1(192)TT143149AA - - +
okadaic acid (1 µM) - + +
bortezomib (2 µM) + + +
WB CycT1
1 2 3

Source blot for Figure supplement 2A

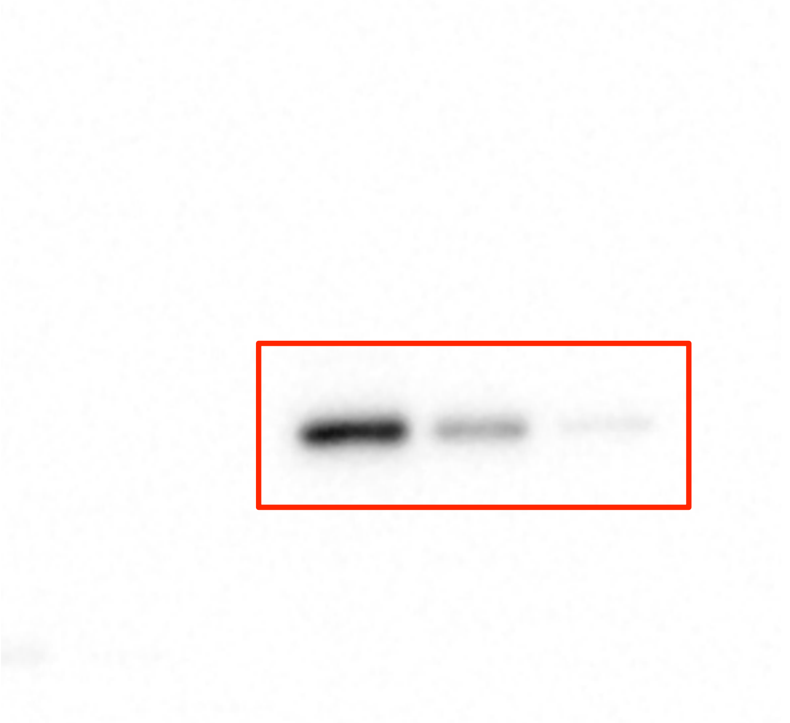

Red marked area was used for the figure.

Membrane was cut after transfer, and before probing by Ab

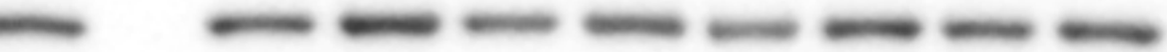

Source blot for Figure supplement 2A

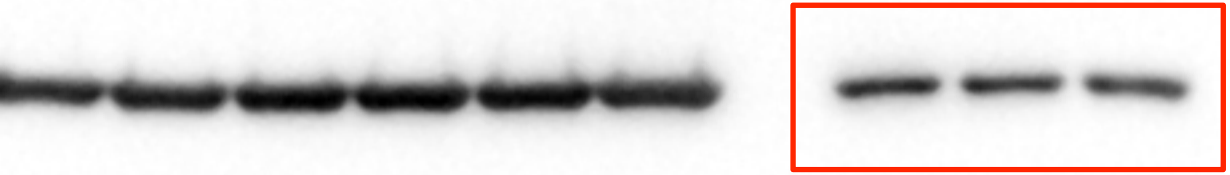

Red marked area was used for the figure.

Membrane was cut after transfer, and before probing by Ab
Two different cut membranes are in the same tray for imaging

Fig. S2. PKC inhibitors promote CycT1 degradation in different cells

293T Cells

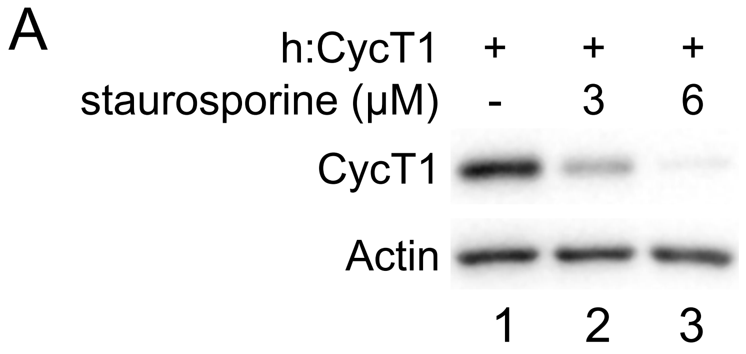

Source blot for Figure supplement 2B

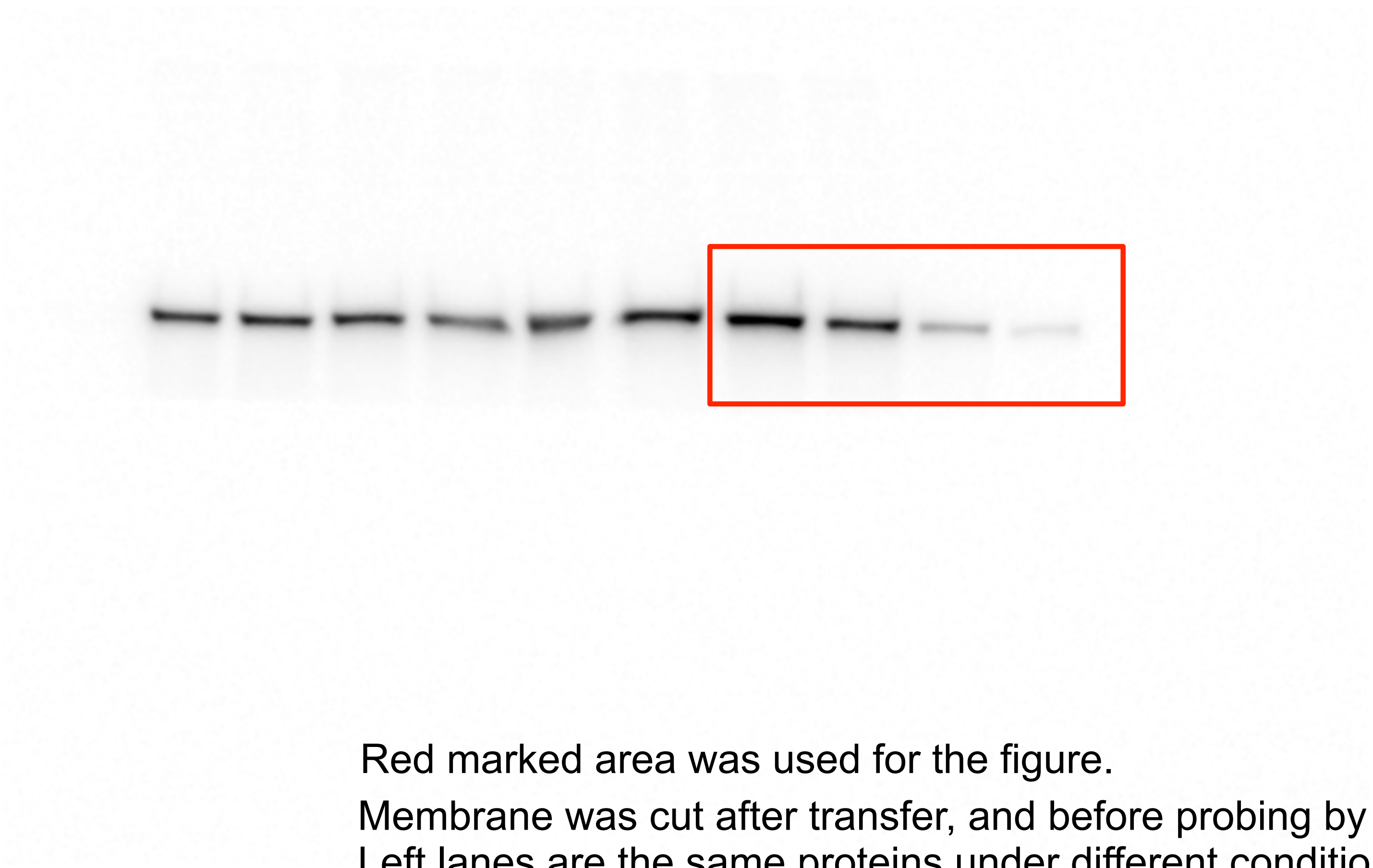

Red marked area was used for the figure.

Membrane was cut after transfer, and before probing by Ab

Left lanes are the same proteins under different conditions

Source blot for Figure supplement 2B

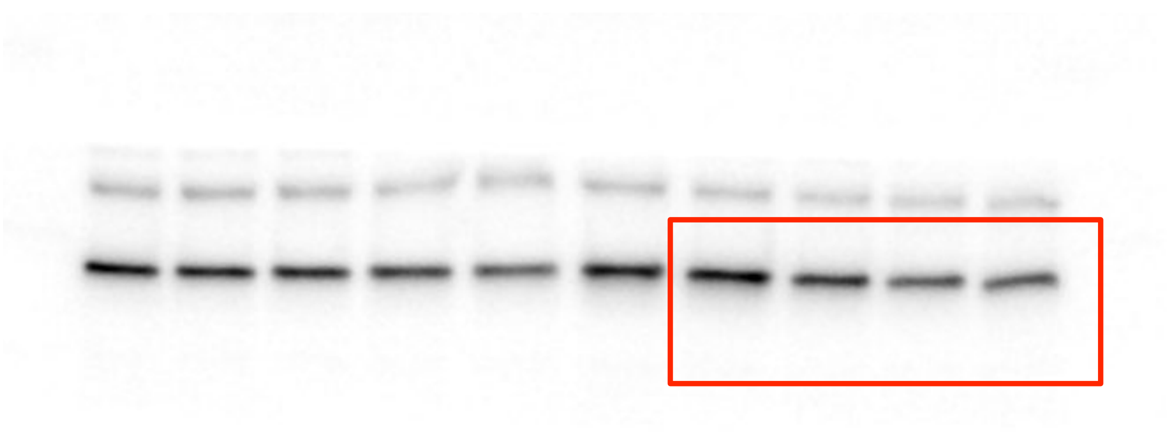

Red marked area was used for the figure.

Membrane was cut after transfer, and before probing by Ab
Left lanes are the same proteins under different conditions

Source blot for Figure supplement 2B

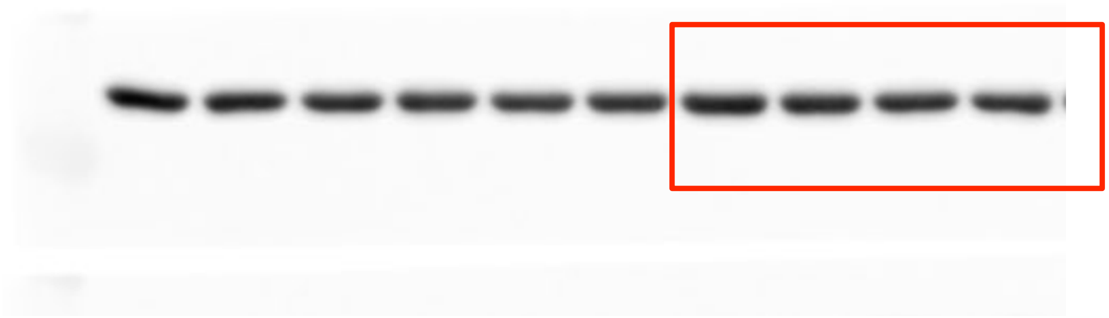

Red marked area was used for the figure.

Membrane was cut after transfer, and before probing by Ab
Left lanes are the same proteins under different conditions

Fig. S2. PKC inhibitors promote CycT1 degradation in different cells

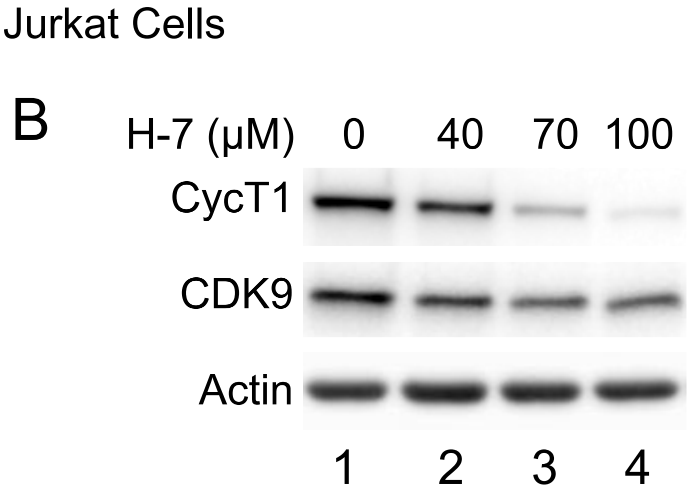

Source blot for Figure supplement 2C

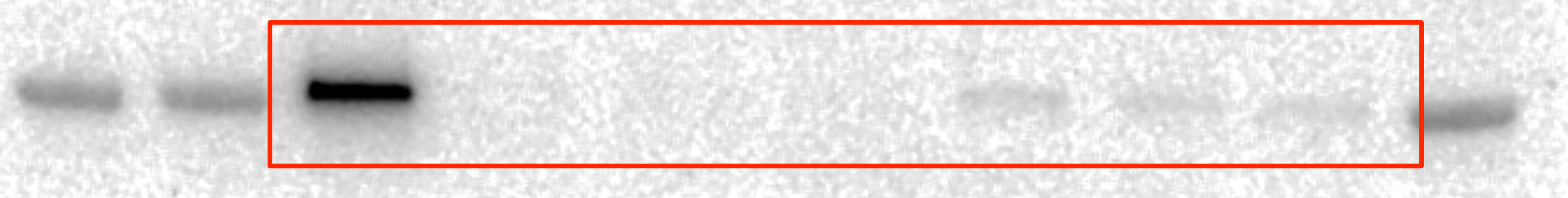

Red marked area was used for the figure.

Membrane was cut after transfer, and before probing by Ab

Source blot for Figure supplement 2C

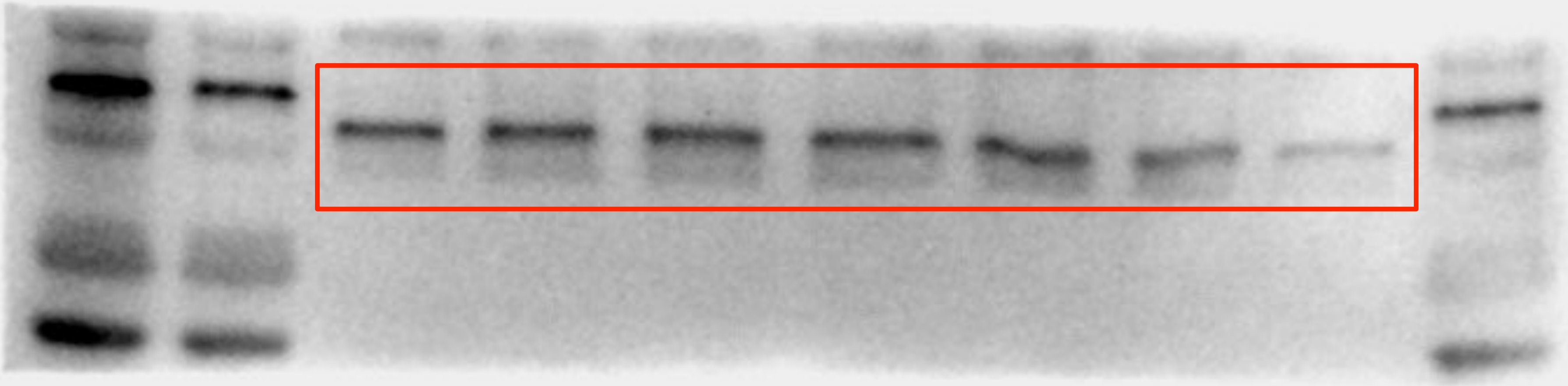

Red marked area was used for the figure.

Membrane was cut after transfer, and before probing by Ab

Source blot for Figure supplement 2C

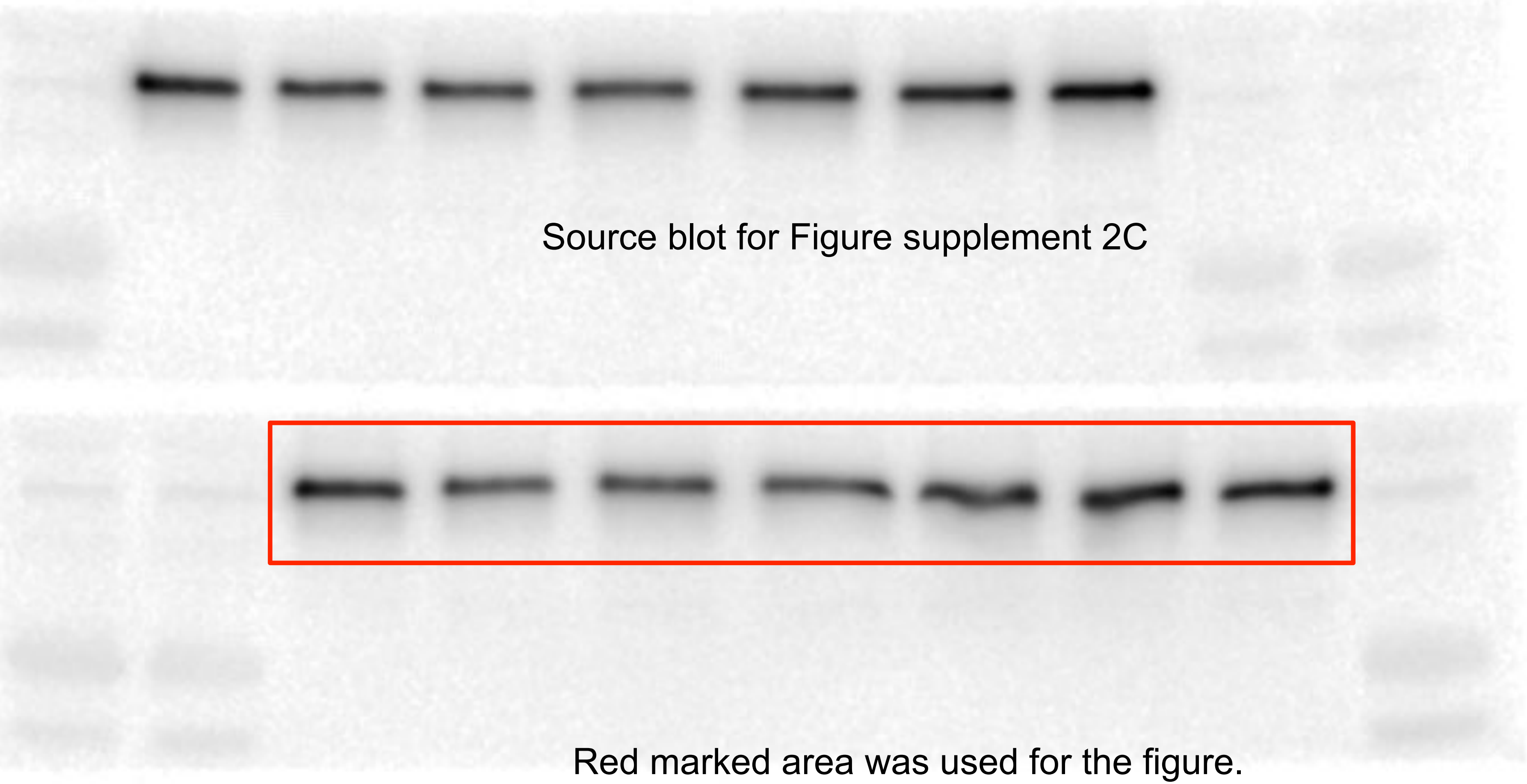

Red marked area was used for the figure.

Membrane was cut after transfer, and before probing by Ab
Two different cut membranes are in the same tray for imaging

Fig. S2. PKC inhibitors promote CycT1 degradation in different cells

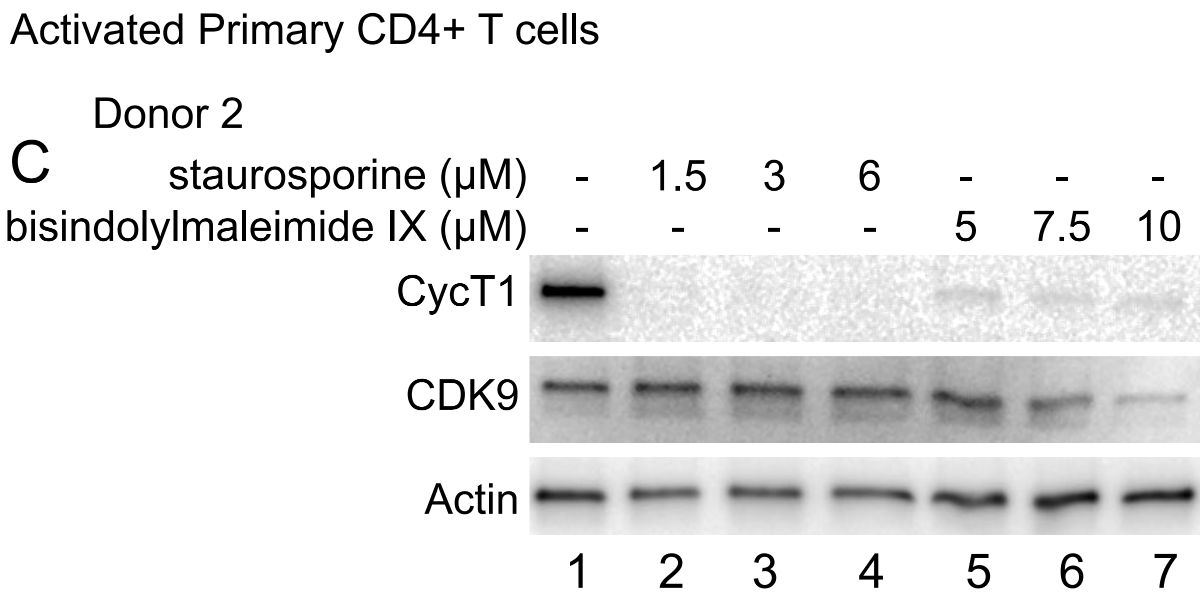

Source blot for Figure supplement 2D

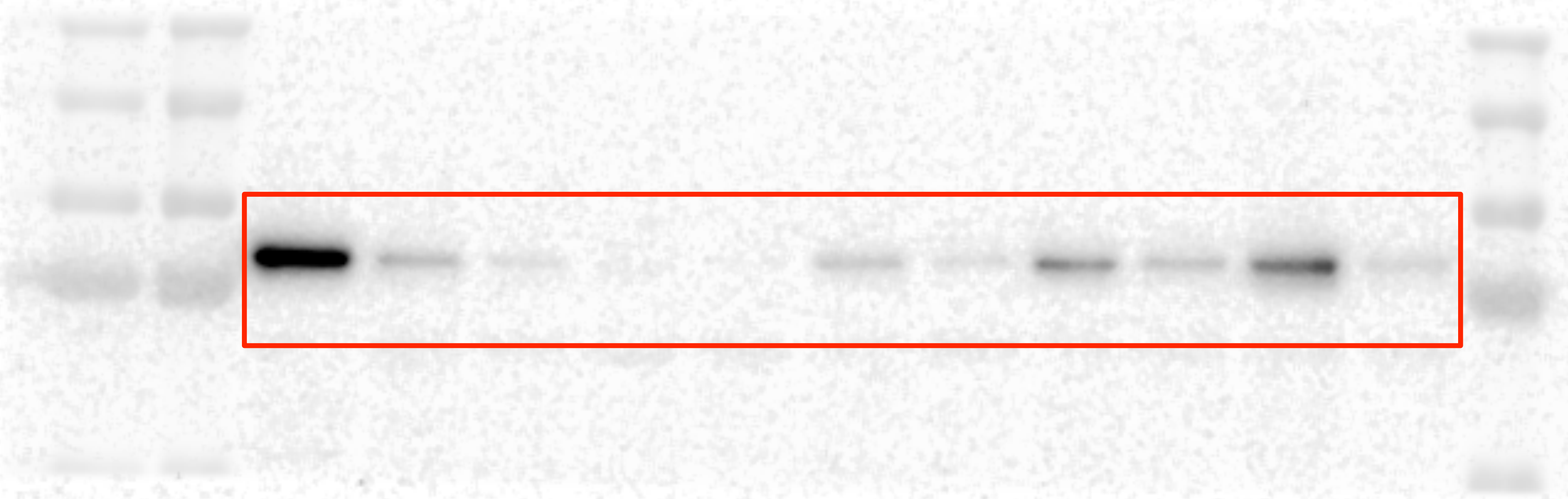

Red marked area was used for the figure.
Membrane was cut after transfer, and before probing by Ab

Source blot for Figure supplement 2D

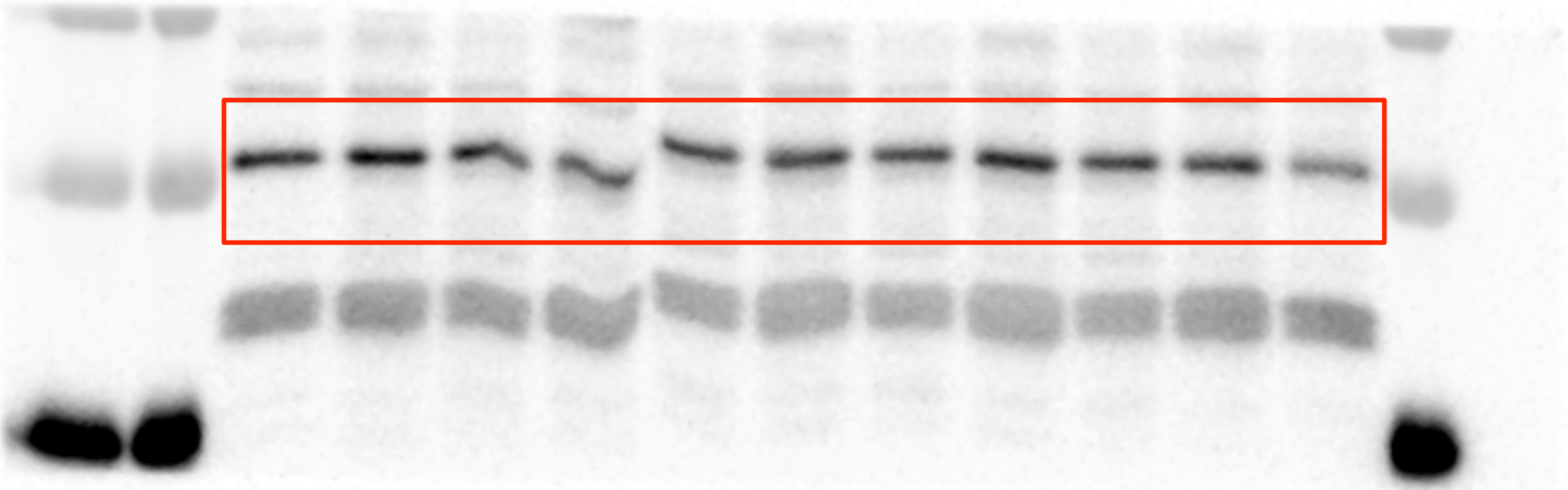

Red marked area was used for the figure.

Membrane was cut after transfer, and before probing by Ab

Source blot for Figure supplement 2D

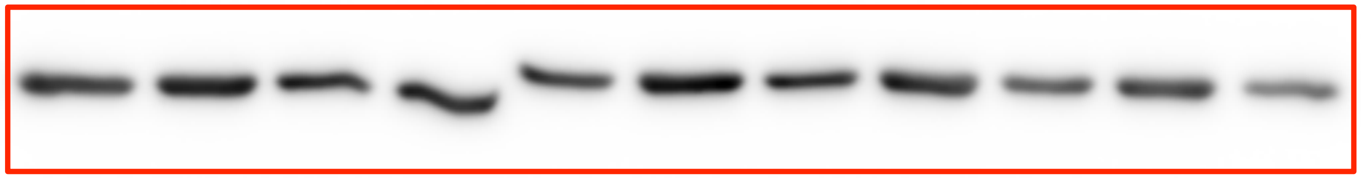

Red marked area was used for the figure.

Membrane was cut after transfer, and before probing by Ab

Fig. S2. PKC inhibitors promote CycT1 degradation in different cells

Activated Primary CD4+ T cells

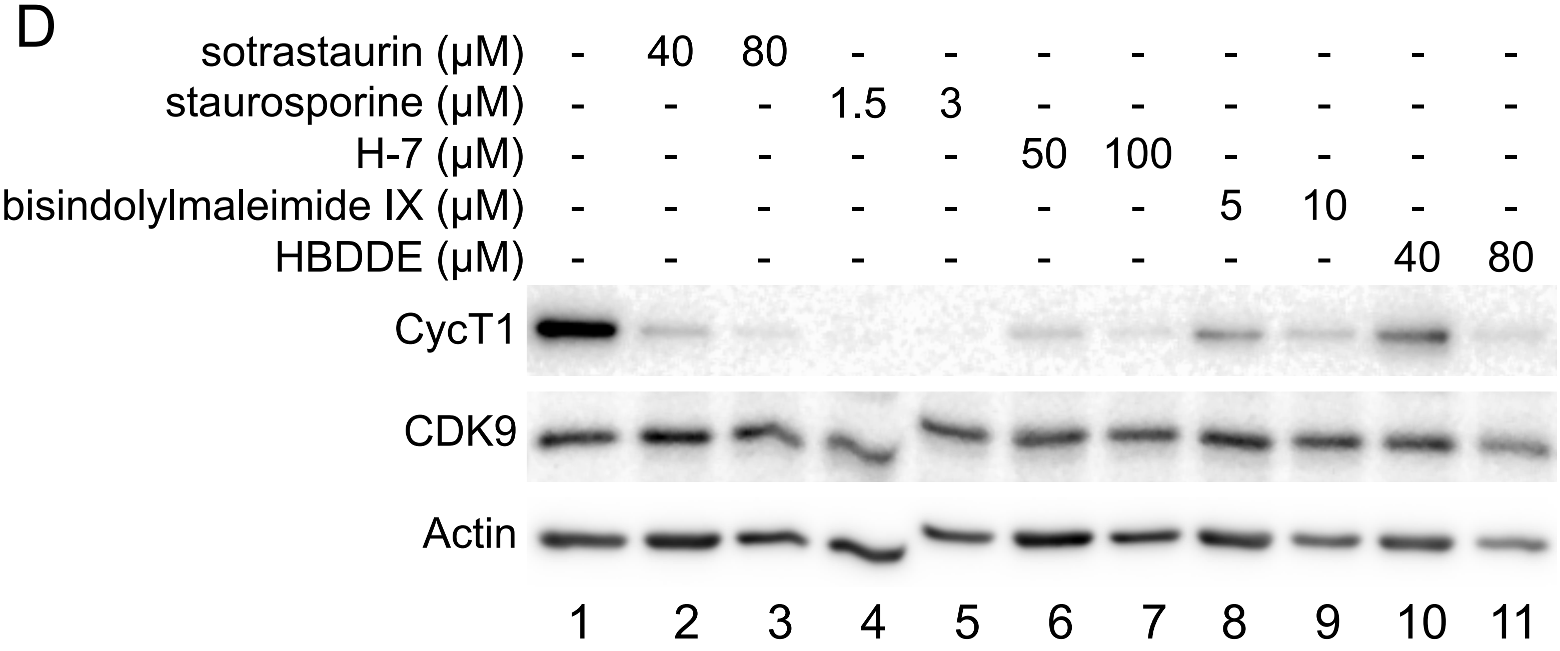

Source blot for Figure supplement 2E

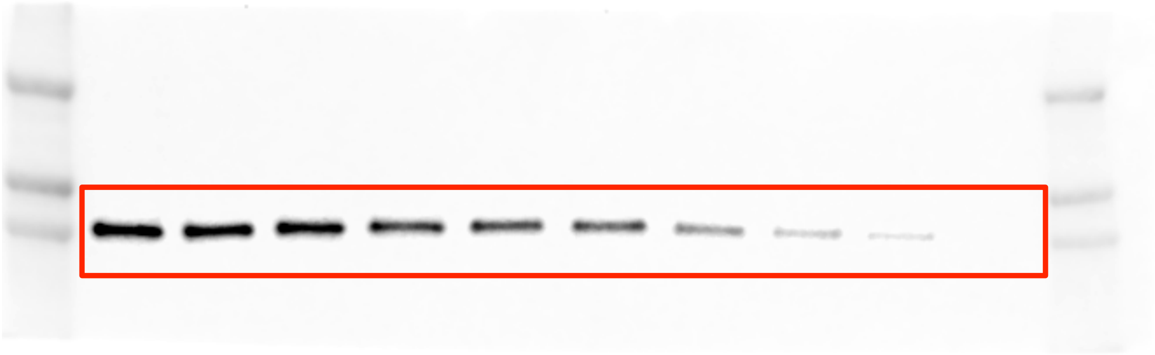

Red marked area was used for the figure.

Membrane was cut after transfer, and before probing by Ab

Source blot for Figure supplement 2E

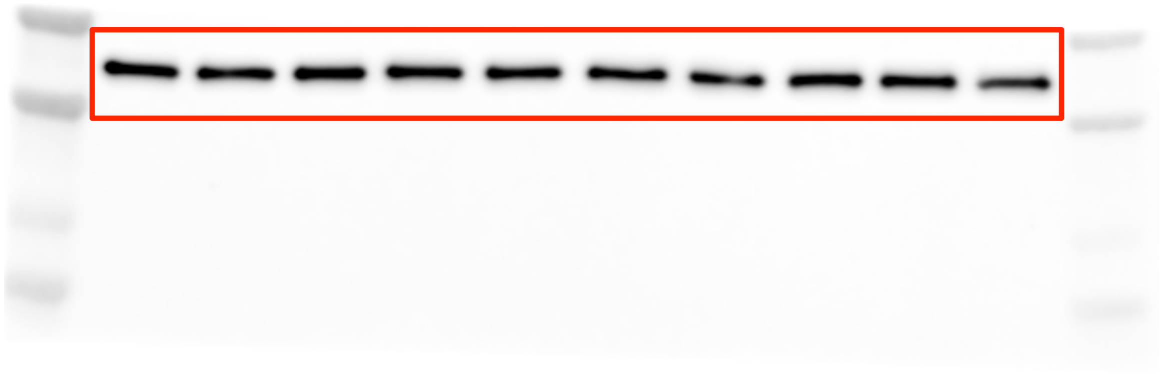

Red marked area was used for the figure.

Membrane was cut after transfer, and before probing by Ab

Fig. S2. PKC inhibitors promote CycT1 degradation in different cells

Activated Primary CD4+ T cells

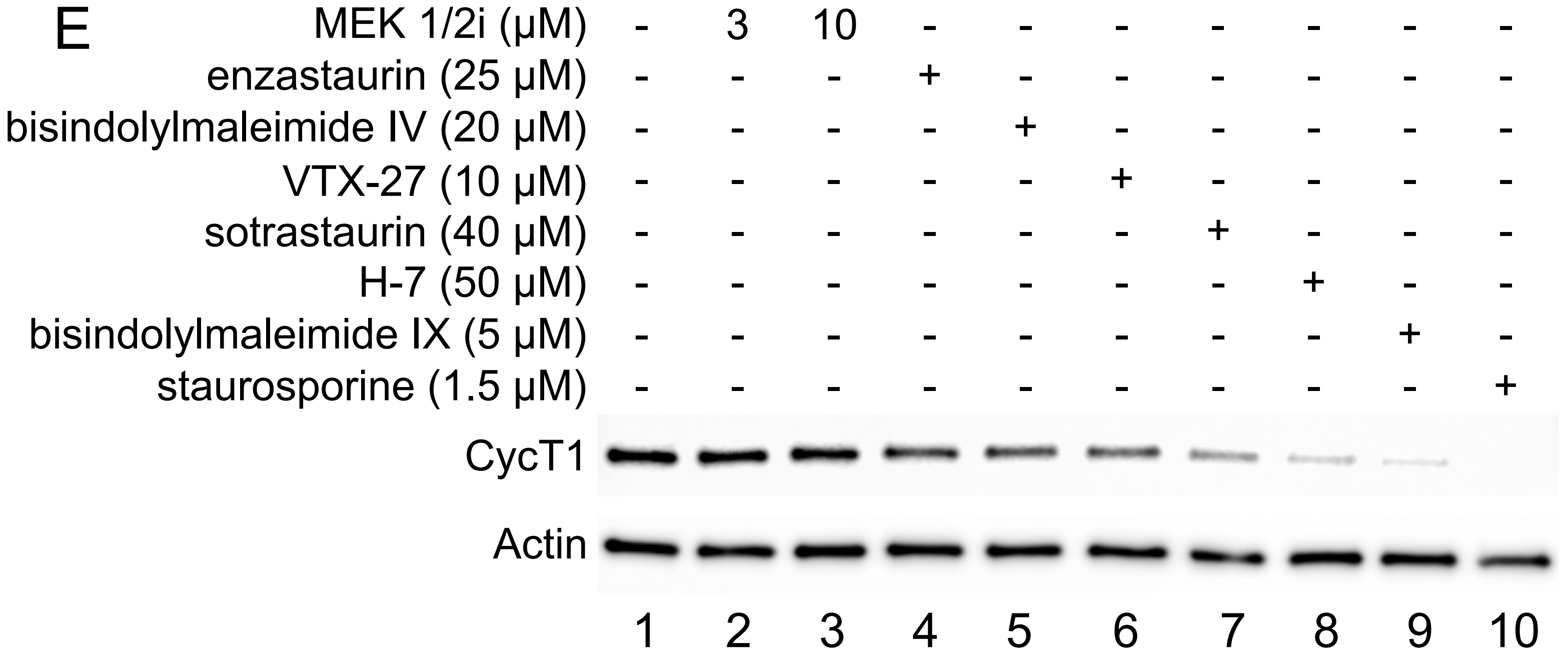

Source blot for Figure supplement 3A

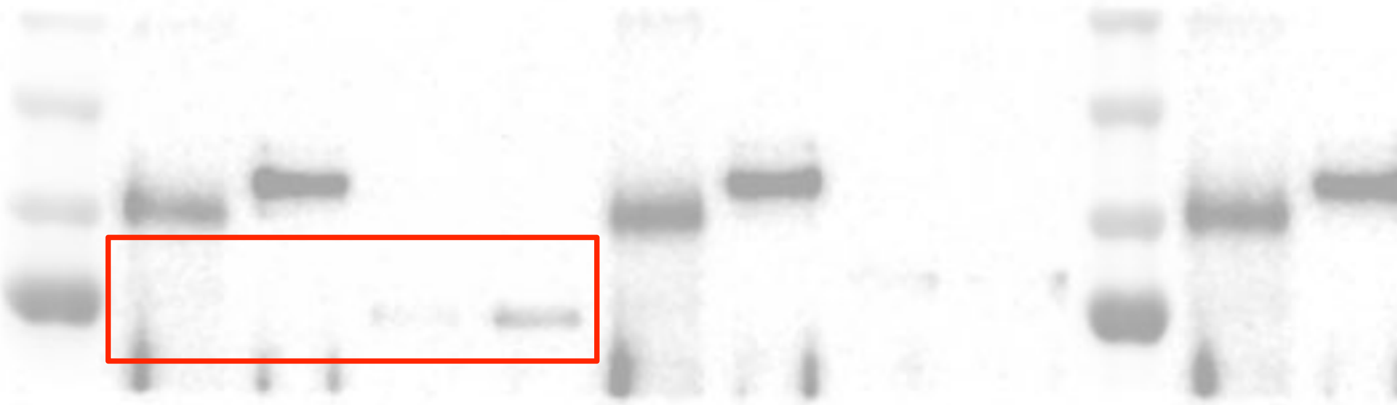

Red marked area was used for the figure.

Membrane was cut after transfer, and before probing by Ab

Source blot for Figure supplement 3A

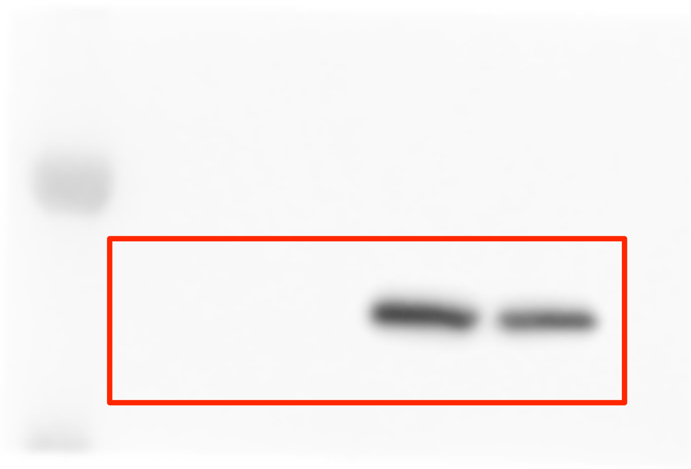

Red marked area was used for the figure.

Membrane was cut after transfer, and before probing by Ab

Source blot for Figure supplement 3A

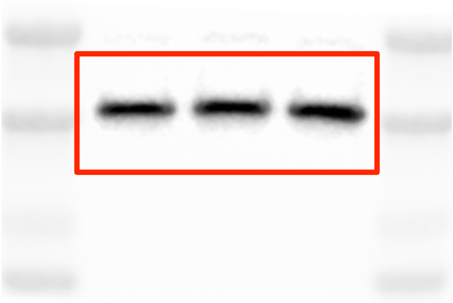

Red marked area was used for the figure.

Membrane was cut after transfer, and before probing by Ab

Source blot for Figure supplement 3A

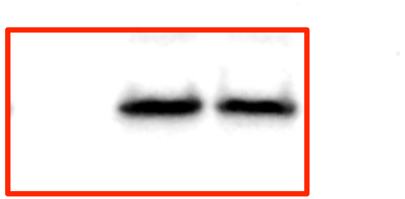

Red marked area was used for the figure.

Membrane was cut after transfer, and before probing by Ab

Fig. S3. PKCδ, PKCγ, and PKCε bind weakly to CycT1

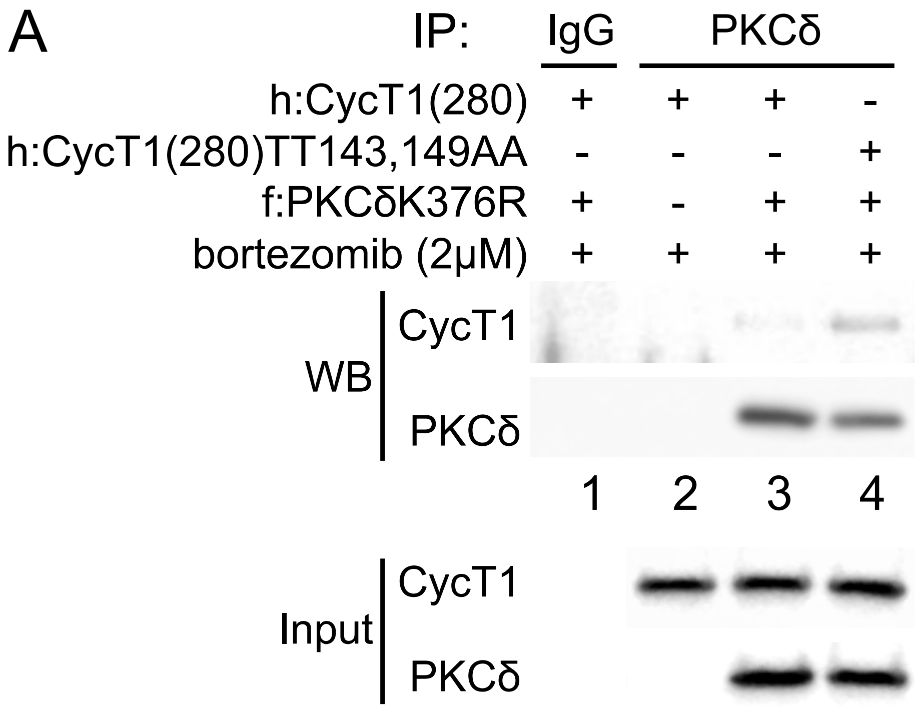

Source blot for Figure supplement 3B

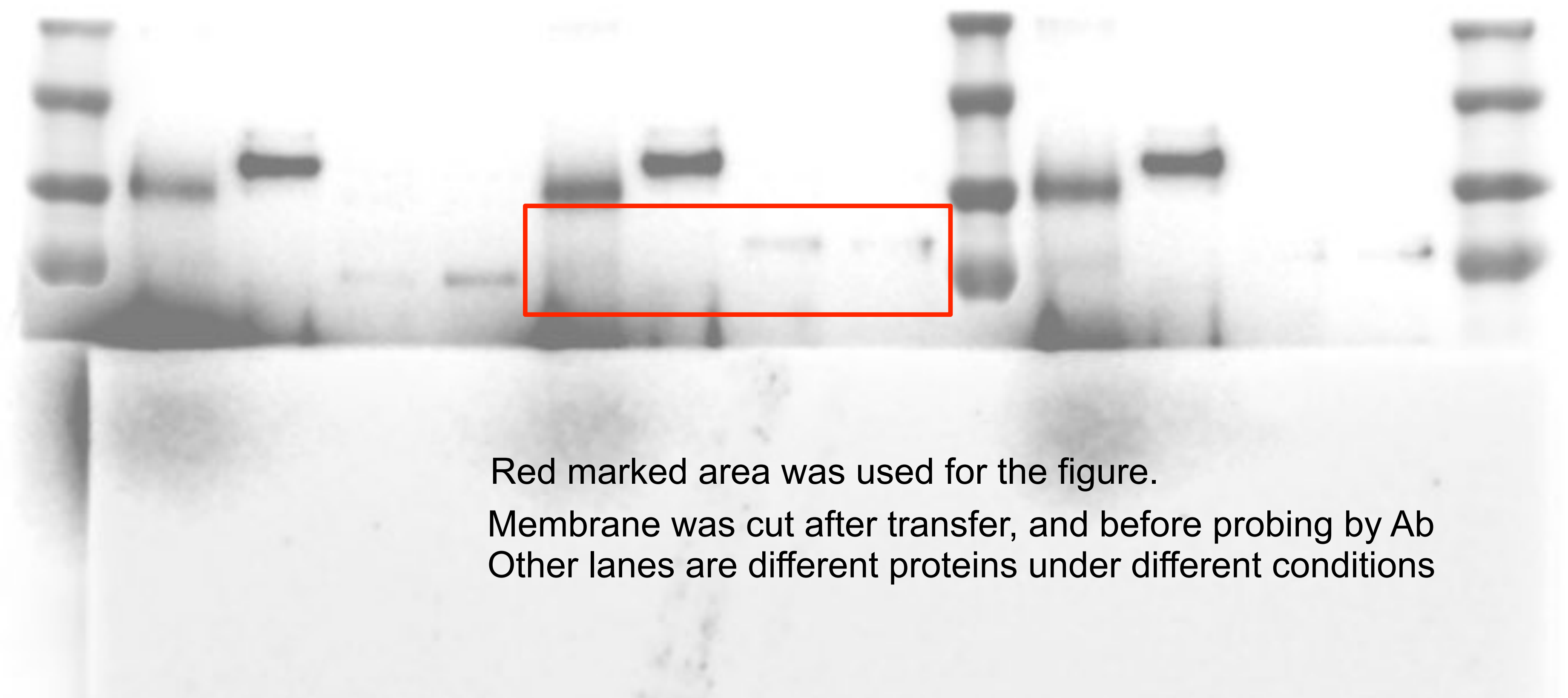

Red marked area was used for the figure.

Membrane was cut after transfer, and before probing by Ab
Other lanes are different proteins under different conditions

Source blot for Figure supplement 3B

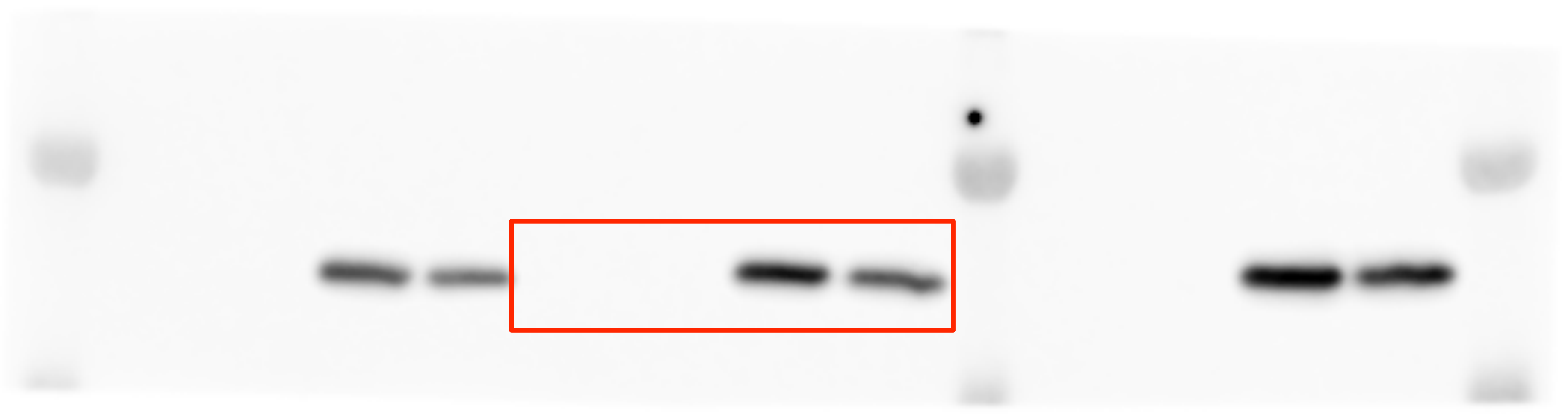

Red marked area was used for the figure.

Membrane was cut after transfer, and before probing by Ab
Other lanes are the same proteins under different conditions

Source blot for Figure supplement 3B

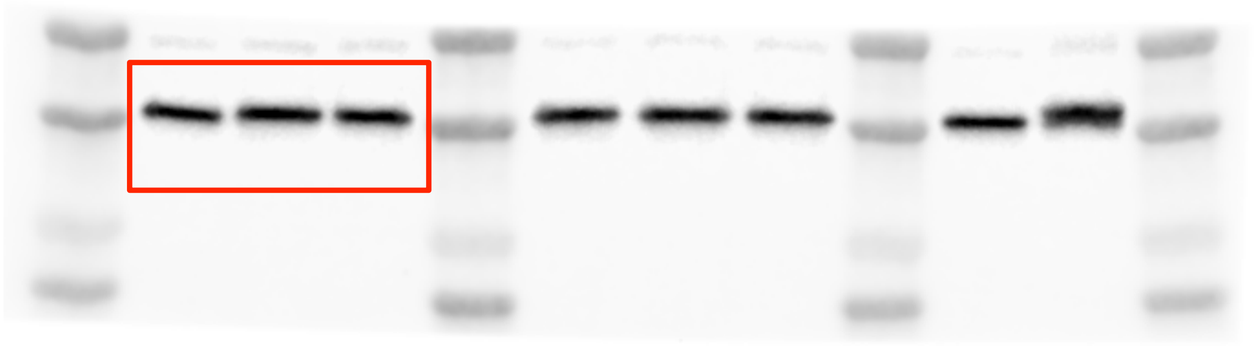

Red marked area was used for the figure.

Membrane was cut after transfer, and before probing by Ab
Other lanes are the same proteins under different conditions

Source blot for Figure supplement 3B

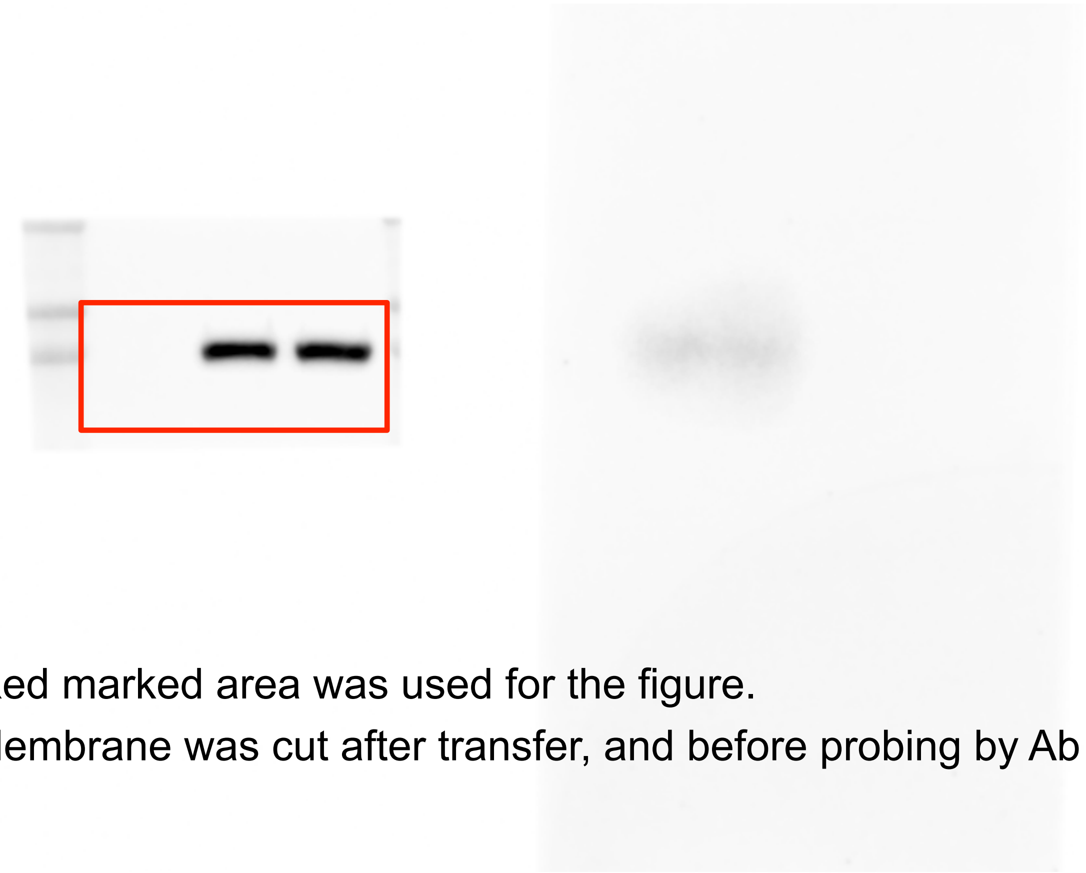

Red marked area was used for the figure.

Membrane was cut after transfer, and before probing by Ab

Fig. S3. PKCδ, PKCγ, and PKCε bind weakly to CycT1

B

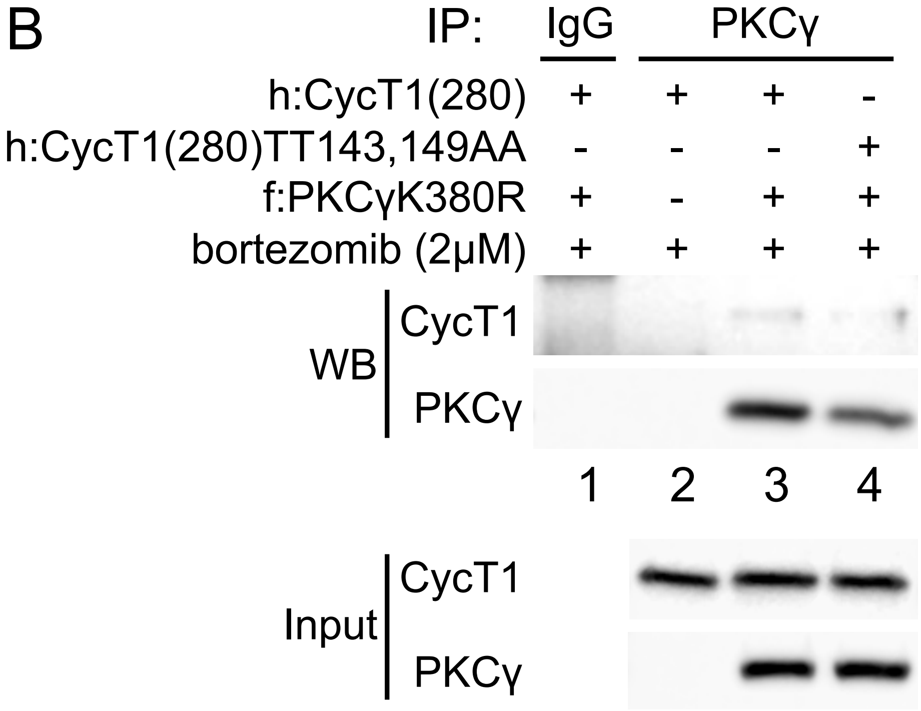

Source blot for Figure supplement 3C

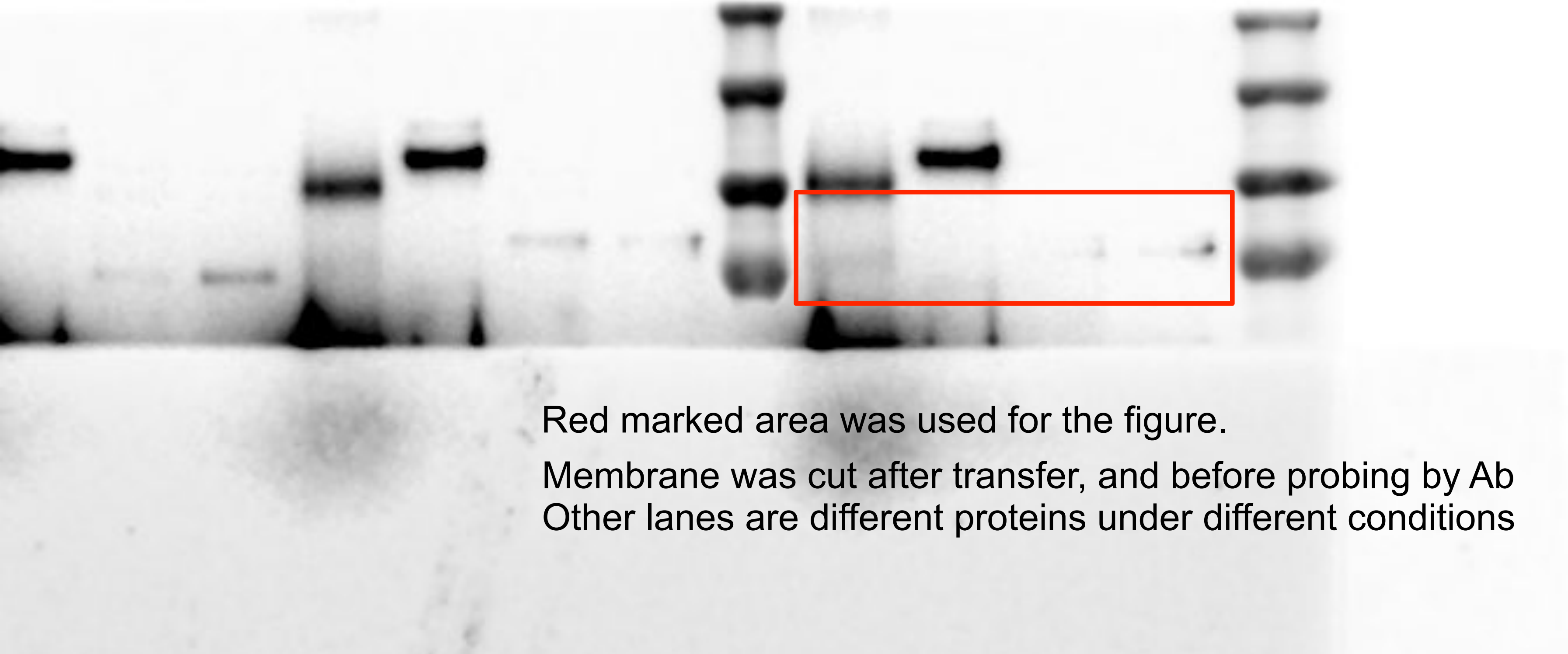

Red marked area was used for the figure.

Membrane was cut after transfer, and before probing by Ab
Other lanes are different proteins under different conditions

Source blot for Figure supplement 3C

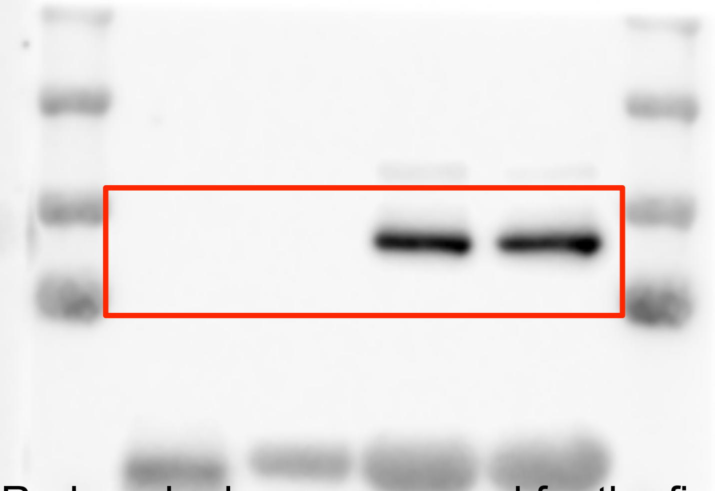

Red marked area was used for the figure.

Membrane was cut after transfer, and before probing by Ab

Source blot for Figure supplement 3C

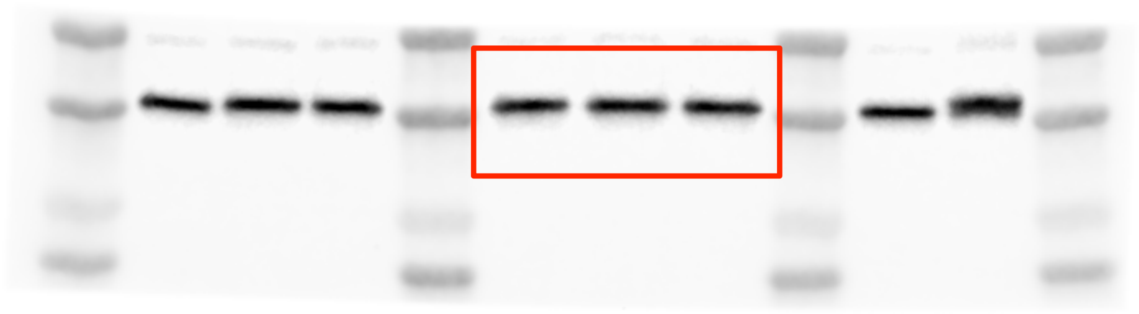

Red marked area was used for the figure.

Membrane was cut after transfer, and before probing by Ab
Other lanes are the same proteins under different conditions

Source blot for Figure supplement 3C

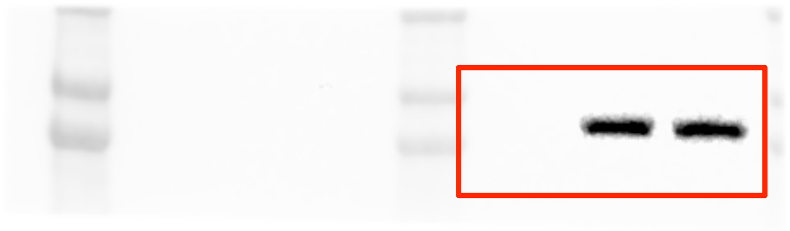

Red marked area was used for the figure.

Membrane was cut after transfer, and before probing by Ab

Fig. S3. PKCδ, PKCγ, and PKCε bind weakly to CycT1

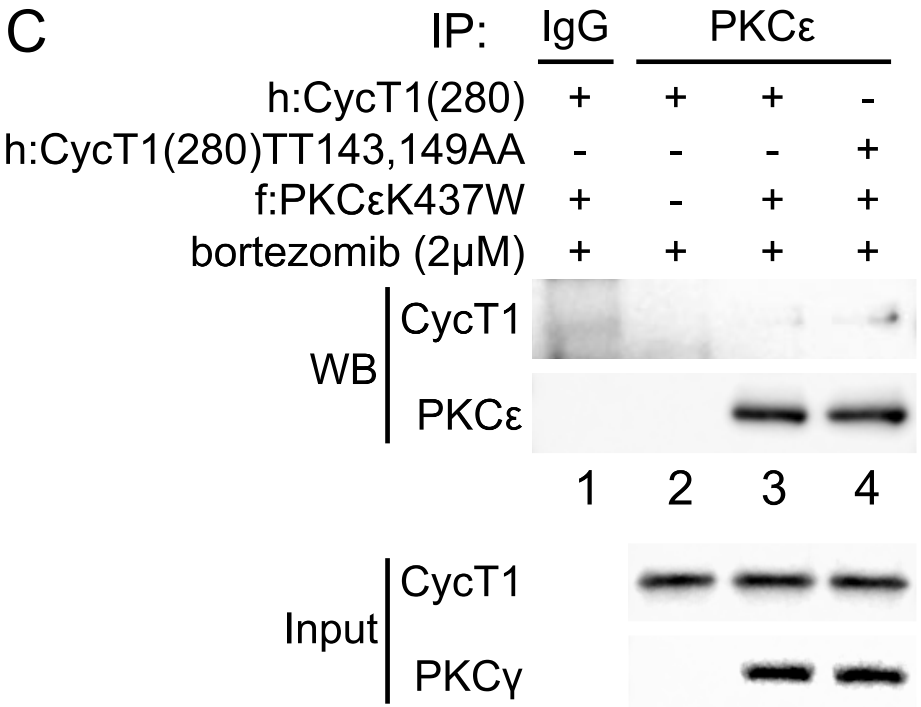

Source blot for Figure supplement 4A

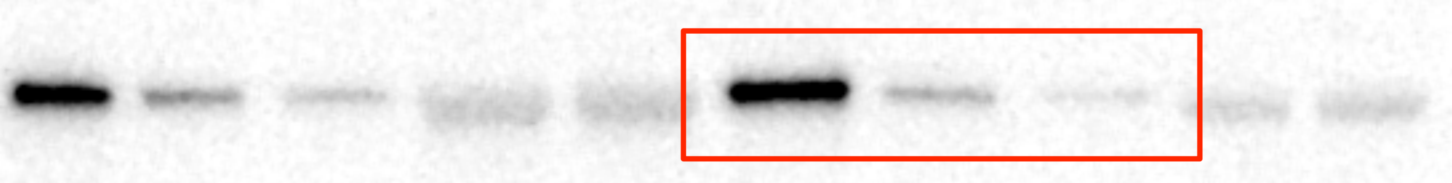

Red marked area was used for the figure.

Membrane was cut after transfer, and before probing by Ab
Left lanes the same proteins from 1st donor

Source blot for Figure supplement 4A

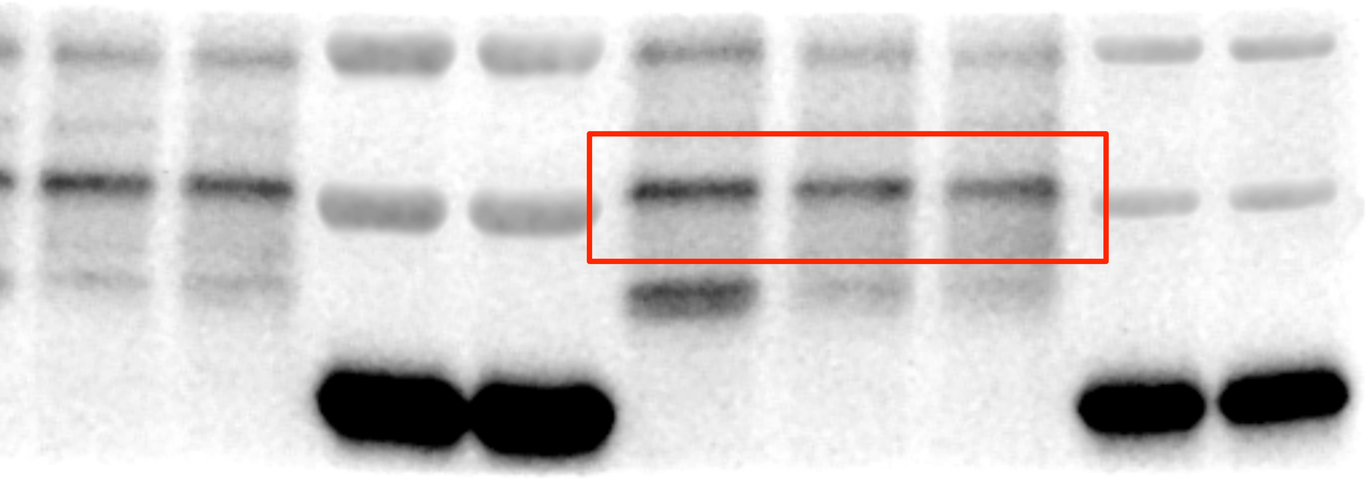

Red marked area was used for the figure.

Membrane was cut after transfer, and before probing by Ab
Left lanes the same proteins from 1st donor

Source blot for Figure supplement 4A

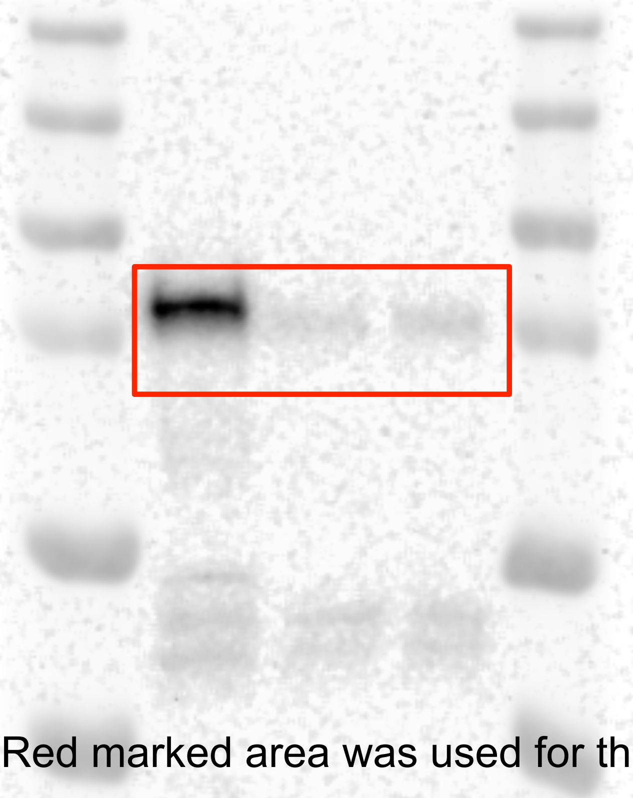

Red marked area was used for the figure.

Membrane was cut after transfer, and before probing by Ab

Source blot for Figure supplement 4A

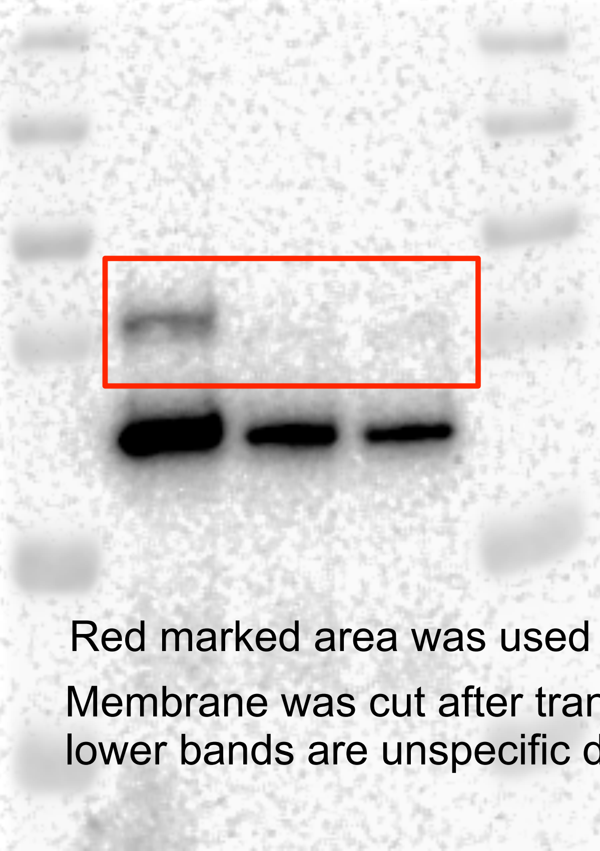

Red marked area was used for the figure.

Membrane was cut after transfer, and before probing by Ab
lower bands are unspecific detection by target antibodies

Source blot for Figure supplement 4A

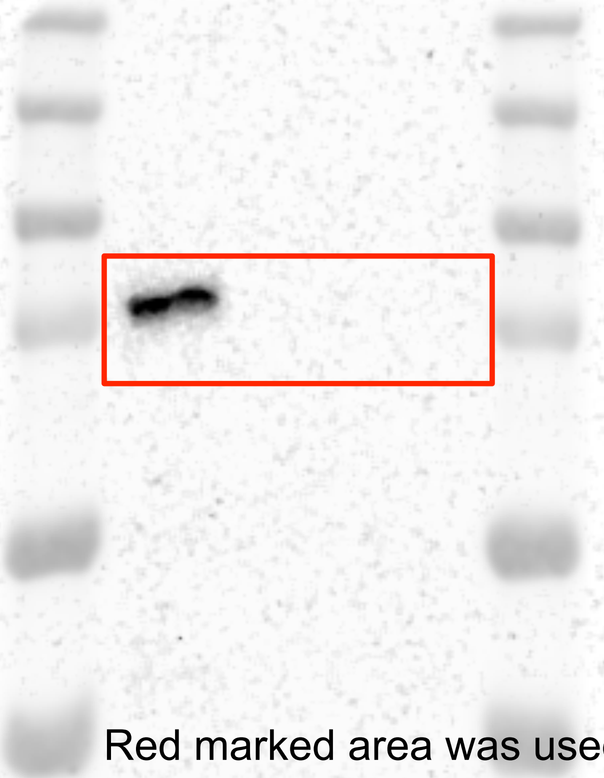

Red marked area was used for the figure.

Membrane was cut after transfer, and before probing by Ab

Source blot for Figure supplement 4A

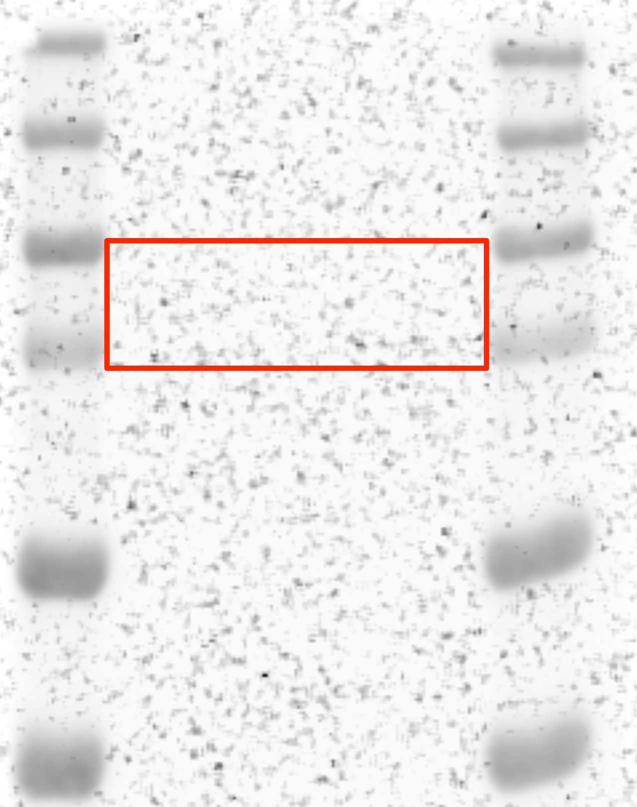

Red marked area was used for the figure.

Membrane was cut after transfer, and before probing by Ab

Source blot for Figure supplement 4A

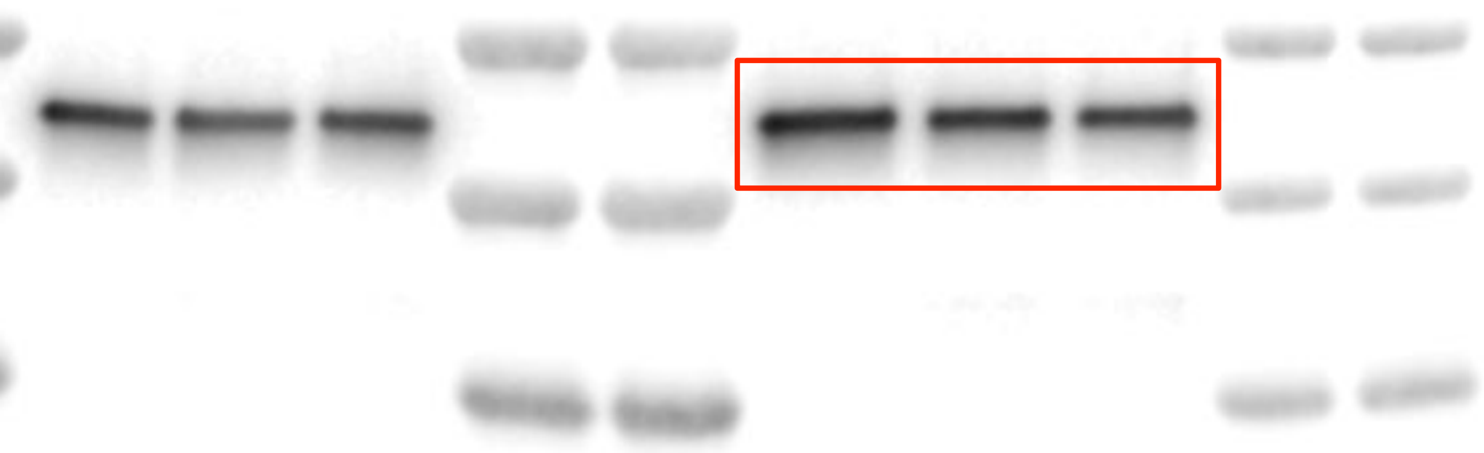

Red marked area was used for the figure.

Membrane was cut after transfer, and before probing by Ab
Left lanes the same proteins from 1st donor

Fig. S4. Chronic activation in primary cells decreases levels of endogenous CycT1 protein

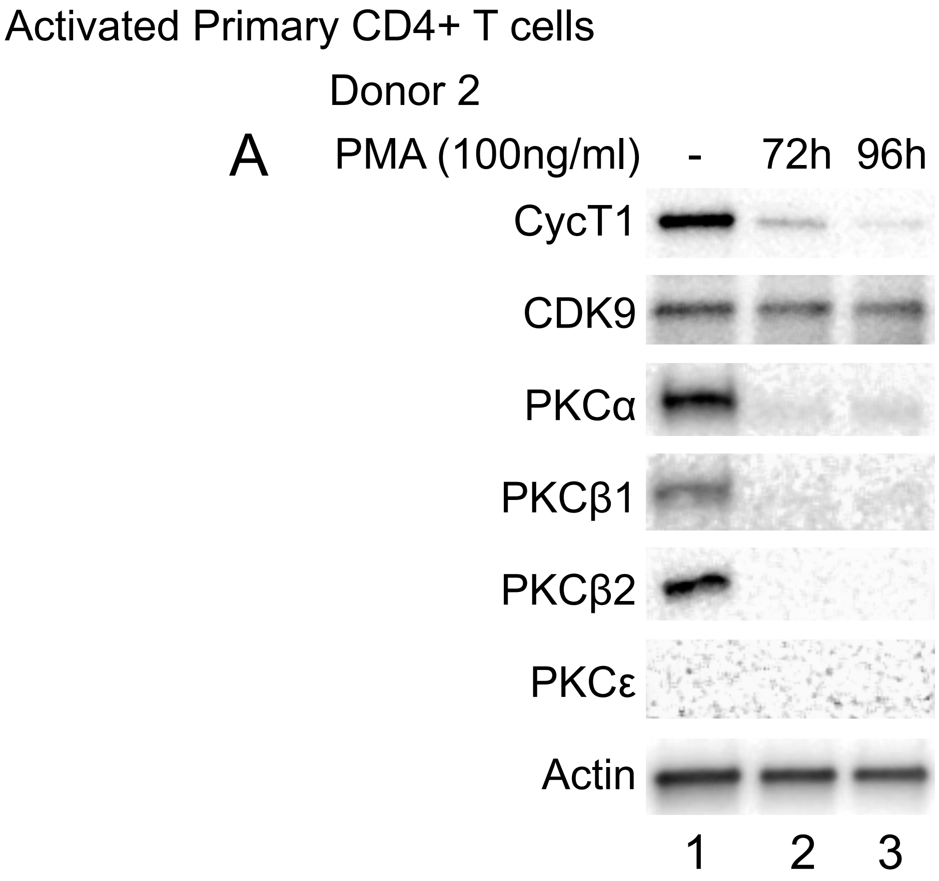

Source blot for Figure supplement 4B

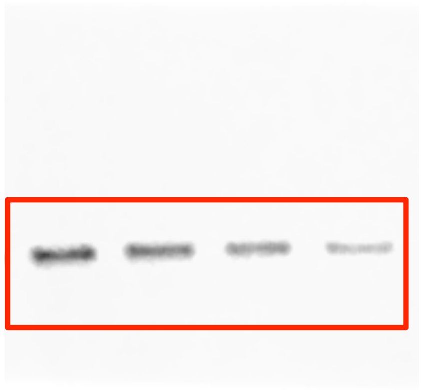

Red marked area was used for the figure.

Membrane was cut after transfer, and before probing by Ab

Source blot for Figure supplement 4B

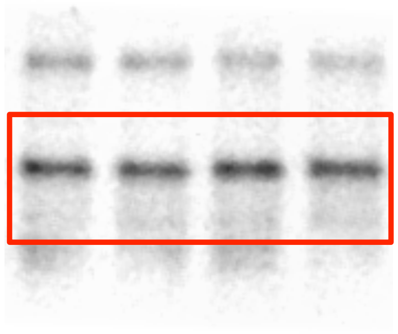

Red marked area was used for the figure.

Membrane was cut after transfer, and before probing by Ab

Source blot for Figure supplement 4B

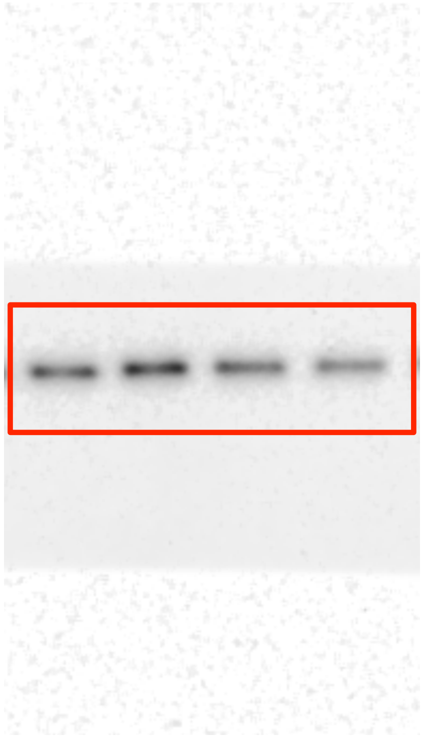

Red marked area was used for the figure.

Membrane was cut after transfer, and before probing by Ab

Fig. S4. Chronic activation in primary cells decreases levels of endogenous CycT1 protein

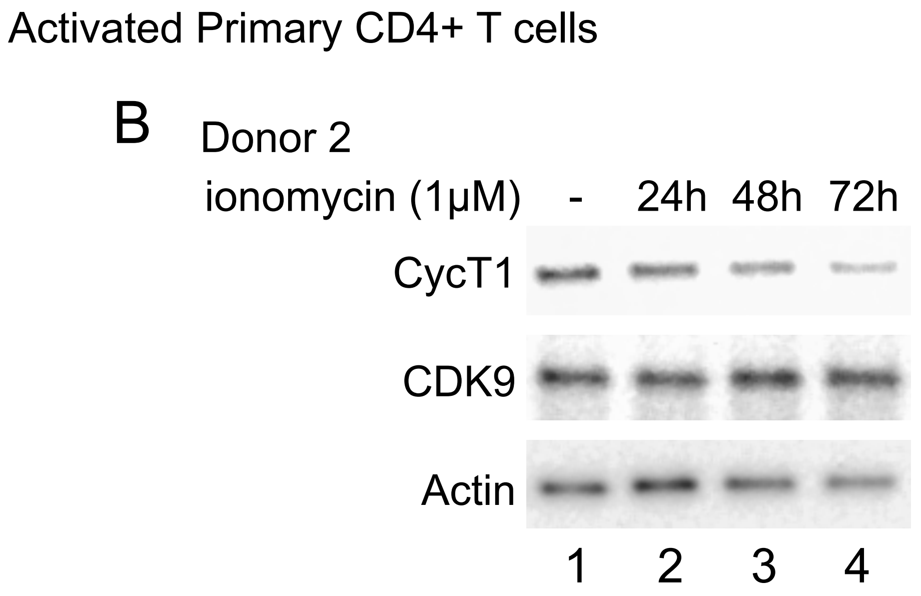

Supplement: Source data 1. [file elife-68473-supp1.pdf]
